# Generation of hydroxyl radical-activatable ratiometric near-infrared bimodal probes for early monitoring of tumor response to therapy

**DOI:** 10.1038/s41467-021-26380-y

**Published:** 2021-10-22

**Authors:** Luyan Wu, Yusuke Ishigaki, Wenhui Zeng, Takashi Harimoto, Baoli Yin, Yinghan Chen, Shiyi Liao, Yongchun Liu, Yidan Sun, Xiaobo Zhang, Ying Liu, Yong Liang, Pengfei Sun, Takanori Suzuki, Guosheng Song, Quli Fan, Deju Ye

**Affiliations:** 1 State Key Laboratory of Analytical Chemistry for Life Science, Chemistry and Biomedicine Innovation Center (ChemBIC), School of Chemistry and Chemical Engineering, Nanjing University, Nanjing, 210023 China; 2 Department of Chemistry, Faculty of Science, Hokkaido University, N10 W8, North-ward, Sapporo, 060-0810 Japan; 3 State Key Laboratory for Chemo/Bio-Sensing and Chemometrics, College of Chemistry and Chemical Engineering, Hunan University, Changsha, 410082 China; 4 Jiangsu Key Laboratory of Advanced Organic Materials, School of Chemistry and Chemical Engineering, Nanjing University, Nanjing, 210023 China; 5 State Key Laboratory of Organic Electronics and Information Displays & Institute of Advanced Materials (IAM), Nanjing University of Posts & Telecommunications, 9 Wenyuan Road, Nanjing, 210023 China

**Keywords:** Cancer imaging, Biomedical materials

## Abstract

Tumor response to radiotherapy or ferroptosis is closely related to hydroxyl radical (•OH) production. Noninvasive imaging of •OH fluctuation in tumors can allow early monitoring of response to therapy, but is challenging. Here, we report the optimization of a diene electrochromic material (1-Br-Et) as a •OH-responsive chromophore, and use it to develop a near-infrared ratiometric fluorescent and photoacoustic (FL/PA) bimodal probe for in vivo imaging of •OH. The probe displays a large FL ratio between 780 and 1113 nm (FL_780_/FL_1113_), but a small PA ratio between 755 and 905 nm (PA_755_/PA_905_). Oxidation of 1-Br-Et by •OH decreases the FL_780_/FL_1113_ while concurrently increasing the PA_755_/PA_905_, allowing the reliable monitoring of •OH production in tumors undergoing erastin-induced ferroptosis or radiotherapy.

## Abstract (web summary)

The hydroxyl radical is generated during radiotherapy and ferroptosis and accurate imaging of this reactive oxygen species may permit the monitoring of response to therapy. Here, the authors develop a ratiometric probe for accurate imaging of hydroxyl radical generation in vivo.

## Introduction

Hydroxyl radical (•OH) is regarded as the most reactive and toxic reactive oxygen species (ROS) in biology, participating in many pathological processes^1,2^. As •OH can react with nearly all endogenous biomolecules, the upregulation of its levels can cause oxidative stress and induce cell death^3,4^. Accordingly, people have proposed several cancer treatment approaches involving the selective generation of •OH in cancer cells to inhibit cancer growth and progression^5–7^. For example, radiation therapy (RT) is a clinically widely used cancer treatment strategy referring to radiation of tumor tissues by ionizing beams (e.g., X-ray)^8–11^; there is mounting evident that the •OH production via radiolysis of water molecules plays an important role in killing cancer cells, as more than 50% of DNA damage in a standard radiotherapy is caused by •OH^3,12^. Ferroptosis, a recently discovered iron-dependent non-apoptotic cell death, is mainly occurred through lipid peroxidation by the intracellular excess of iron-mediated generation of •OH^13–15^; the selective accumulation of iron in tumor cells to elicit ferroptosis has emerged as an effective approach for cancer treatment^16^. More recently, chemodynamic therapy that employs the Fenton-like reaction to produce •OH selectively in tumor cells has also been proposed as an alternative for cancer treatment^17–19^. While these •OH-related cancer treatment approaches have shown promise to combat cancer, the •OH production and therapeutic efficacy generally vary in different types of cancer, owing to the heterogeneity of cancer that can cause treatment resistant^20,21^. Methods that allow for real-time monitoring of •OH production in tumors during RT or other •OH-related therapy may provide invaluable information for early evaluating the therapeutic efficacy and optimizing therapeutic intervention.

In the past few decades, several analytical methods, including electron spin resonance (ESR) spectroscopy^22,23^, ultraviolet–visible (UV–vis) chromatography^24^, and fluorescence (FL) imaging^25–29^, have been devoted for the detection of •OH. Among them, FL imaging of •OH has attracted tremendous attention due to the advantage of high sensitivity, easy operation, rapid acquirement, and minimum invasiveness^30–32^. To date, a number of FL probes have been developed to offer sensitive signals for the measurement of •OH^25–29^. However, most of them emit FL in visible regions; the severe absorption and scattering of visible light by biological tissues have precluded their ability for noninvasive in vivo imaging of •OH. While a few FL probes capable of emitting their FL in near-infrared (NIR) regions have been reported, none of them has been applied for in vivo imaging of tumor •OH^28,33–36^. Moreover, they were all designed based on the change of FL intensity at a single wavelength, which could be largely influenced by the fluctuation of probe’s concentration in the dynamic and complex in vivo environments, potentially leading to false-positive results. People have recently reported several ratiometric FL imaging probes based on a FL intensity ratio at two different wavelengths, which might improve accuracy for the detection of intracellular •OH as the built-in self-calibration effect could allow to minimize false-positive errors resulting from environmental factors^28,37–39^. However, most of them also emitted their FL in the visible regions and exhibited relatively slow reaction kinetics toward •OH, impeding their ability for in vivo imaging of •OH. A refined •OH-responsive ratiometric FL probe with their FL emissions both in the NIR regions and a fast reaction kinetics toward •OH may benefit to improve in vivo imaging of tumor •OH, but is remaining elusive.

In addition to FL imaging, the recently emerging photoacoustic (PA) imaging technology has been of particular interest for in vivo imaging as it can produce three-dimensional images with high spatial-resolution (up to micrometers) and deep tissue-penetration (up to centimeters)^40,41^. There have been a number of PA probes developed for in vivo imaging of biomolecules and biological environments, such as nitric oxide^42^, hypoxia^43^, acidic pH^44,45^, ROS^46,47^, and enzymes^48,49^. In addition, a few ratiometric PA probes have been also reported for the detection of ROS like H_2_O_2_ and ONOO^−^, showing improved penetration depth and spatial-resolution over traditional NIR FL imaging^50–52^. However, till now, there is still lack of a PA probe capable of selectively in vivo imaging of tumor •OH, let alone a ratiometric PA probe for •OH. Considering that NIR FL imaging holds high sensitivity while PA imaging holds good spatial resolution and deep penetration^53–55^, we envision that a ratiometric NIR FL/PA bimodal probe with the combination of FL and PA imaging may offer complementary advantages to enable refined in vivo imaging of tumor •OH. Moreover, such a ratiometric bimodal probe may permit self-calibration of •OH-independent factors based on the built-in two ratiometric signals, potentially avoiding the occurrence of false-positive signals and consequently improving accuracy for noninvasive imaging of •OH in tumors response to RT or ferroptosis.

**Fig. 1:**
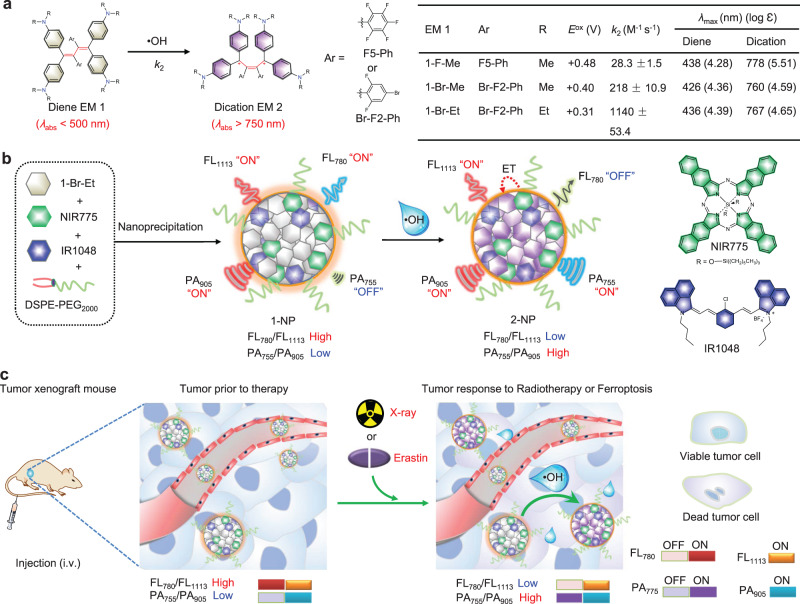
Schematic illustration of the design of 1-NP. **a** The chemical structures of diene EM 1-F-Me, 1-Br-Me, and 1-Br-Et, and proposed conversion into dication EMs 2-F-Me, 2-Br-Me, and 2-Br-Et upon oxidation by •OH. Right: summary of redox potentials (*E*^ox^), second-order reaction rate (*k*_2_), maximum absorption (*λ*_amx_) and extinction coefficient (log ε) of EMs. **b** Schematic illustration of preparation of 1-NP via DSPE-PEG_2000_-assisted encapsulation of 1-Br-Et, NIR775 and IR1048, and proposed conversion of 1-NP into 2-NP in response to •OH, accompanying by a reduced fluorescence (FL_780_/FL_1113_) ratio but a concurrently increased photoacoustic (PA_755_/PA_905_) ratio. Right: chemical structures of NIR775 and IR1048 used for the preparation of 1-NP. **c** Illustration of the mechanism of 1-NP for ratiometric near-infrared (NIR) FL/PA bimodality imaging of •OH production in tumors undergoing X-ray radiotherapy (RT) or erastin-induced ferroptosis. Following intravenous (i.v.) injection, 1-NP accumulates in tumor tissues via the enhanced permeability and retention (EPR) effect. In tumor cells prior to therapy with X-ray irradiation or erastin, 1-NP dominants in the tumor tissues due to the low endogenous •OH level, and the tumors show a high FL_780_/FL_1113_ but a low PA_755_/PA_905_; in tumor cells response to the applied therapy, the •OH level is elevated, which triggers the conversion of 1-NP into 2-NP, resulting in a low FL_780_/FL_1113_ but a high PA_755_/PA_905_.

In this study, we report a •OH-responsive ratiometric NIR FL/PA bimodal imaging nanoprobe (1-NP) for noninvasive monitoring of tumor response to RT or erastin-induced ferroptosis (Fig. 1). This nanoprobe is designed on the basis of our works on synthesis and engineering of organic π-electron electrochromic materials (EMs), which distinctly switch color upon electron transfer (i.e., a redox process), to build activatable optical probes for in vivo imaging^56–58^. To achieve fast reaction kinetics toward •OH, three EM 1 (e.g., 1-F-Me, 1-Br-Me and 1-Br-Et) sharing the same diene scaffold are synthesized and screened (Fig. 1a). EM 1-Br-Et with the fastest reaction kinetics is chosen as the •OH-specific chromophore, which can be rapidly converted into dication 2-Br-Et upon reaction with •OH, resulting in UV–vis absorption shifted distinctly from 436 to 767 nm. We envision that the appearance of NIR absorption of 2-Br-Et at 767 nm can potentially quench the fluorescence of a NIR fluorophore and concurrently augment the PA signal. By encapsulating 1-Br-Et, a NIR-I fluorophore of silicon 2,3-naphthalocyanine bis(trihexylsilyloxide (NIR775) and a NIR-II fluorophore of 1-butyl-2-[2-[3-[(1-butyl-6-chlorobenz[cd]indol-2(1H)-ylidene)ethylidene]-2-chloro-1-cyclohexen-1yl] ethenyl]-6-chlorobenz[cd]indolium tetrafluoroborate (IR1048) in a micellar nanoparticle, 1-NP is developed (Fig. 1b). As the UV–vis absorption of 1-Br-Et is below 500 nm, 1-NP initially displays high NIR-I FL of NIR775 at 780 nm and NIR-II FL of IR1048 at 1113 nm; the FL ratio between 780 and 1113 nm (FL_780_/FL_1113_) is relatively high. Meanwhile, 1-NP displays weak PA signal at 755 nm while high PA signal of IR1048 at 905 nm, which gives rise a low PA ratio between 755 and 905 nm (PA_755_/PA_905_). Upon reaction with •OH, the 1-Br-Et within 1-NP is oxidized into 2-Br-Et, which possesses a strong absorption at 767 nm; the NIR-I FL of NIR775 in the resulting 2-NP is quenched, presumably owing to an energy-transfer (ET) process from NIR775 to 2-Br-Et, while the NIR-II FL of IR1048 at 1113 nm is little changed. Thus, the FL_780_/FL_1113_ significantly decreased. Concurrently, the PA signal of 2-NP at 755 nm is switched on while the PA signal at 905 nm remained constant, and consequently the PA_755_/PA_905_ increased. Therefore, a significant decline in FL_780_/FL_1113_ but obvious increment in PA_755_/PA_905_ can be simultaneously achieved upon incubation of 1-NP with •OH. Fig. 1c illustrates the mechanism for ratiometric NIR FL/PA bimodality imaging of •OH levels in tumors following radiotherapy or erastin-induced ferroptosis in vivo. After intravenous (i.v.) injection, 1-NP can extravasate and enter tumor tissues owing to the enhanced permeability and retention (EPR) effect. In tumor cells prior to applied therapy, the •OH level is low and 1-NP dominants in the tumor tissues, which show a high FL_780_/FL_1113_ but a low PA_755_/PA_905_. However, in tumor cells receiving therapy with X-ray radiation or erastin, the •OH level is elevated, which permits oxidation of 1-NP into 2-NP, resulting in a low FL_780_/FL_1113_ but a high PA_755_/PA_905_. The distinct switch in FL_780_/FL_1113_ and PA_755_/PA_905_ can provide two mutual correlation signals to allow 1-NP to reliably detect •OH levels in tumors during RT or ferroptosis, which could be useful for the early monitoring of treatment response. Moreover, the ‘always on’ NIR-II FL at 1113 nm and PA signal at 905 nm of IR1048 within 1-NP can also allow to track in vivo delivery, amenable to visualize tumor tissues and guide applied radiotherapy.

## Results

### Engineering of 1-Br-Et into 1-NP

To enable fast response to •OH, we synthesized three reduced EMs (1-F-Me, 1-Br-Me, and 1-Br-Et), which share the same butadiene scaffold but contain different substituents (Fig. 1a). All these three dienes display light yellow color in aqueous solutions, with UV–vis absorption below 500 nm (Supplementary Fig. 1). After incubation with •OH (generated from Fenton reaction between Fe^2+^ and H_2_O_2_)^59^, they could all be oxidized into purple dications (2-F-Me, 2-Br-Me, and 2-Br-Et), with their maximum UV–vis absorption noticeably bathochromic shifting to above 750 nm. We then employed the distinct change of UV–vis absorption to measure the reaction kinetics of 1-F-Me, 1-Br-Me, and 1-Br-Et toward •OH. The apparent second-order reaction rate (*k*_2_) between EM 1 and •OH was found to be 28.3 ± 1.5 M^−1^ s^−1^ for 1-F-Me, 218 ± 10.9 M^−1^ s^−1^ for 1-Br-Me, and 1140 ± 53.4 M^−1^ s^−1^ for 1-Br-Et (Supplementary Fig. 2). These results revealed that 1-Br-Et holds the fastest reaction kinetics toward •OH among these three dienes, which could be owing to the lower *E*^ox^ of 1-Br-Et (+0.31 V vs. SCE) than that of 1-F-Me (+0.48 V vs. SCE) and 1-Br-Me (+0.40 V vs. SCE) (Supplementary Fig. 3 and Fig. 1a). The subsequent selectivity test showed that 1-Br-Et was inert to other reactive oxygen species (ROS, e.g., H_2_O_2_, O_2_^•-^, ONOO^−^, ClO^−^, ^1^O_2_, *t*-BuOOH, CuOOH), and only •OH could oxidize it to form 2-Br-Et (Supplementary Fig. 4). Such a high selectivity could be presumably owing to that •OH hold a larger redox potential (Supplementary Table 1) while a smaller molecular size relative to other ROS, which could be easier to overcome the steric hindrance in 1-Br-Et and oxidize it into 2-Br-Et; the subsequent density functional theory (DFT) calculation further predicted that the reaction between 1-Br-Et and •OH was more energetically favorable (Supplementary Fig. 5 and Dataset 1). We further took the PA images of 1-Br-Et with and without incubation with •OH. As shown in Supplementary Fig. 6, the PA signal for 1-Br-Et alone was very weak at 755 nm, which remarkably increased by ~22-fold after incubation with •OH. Taking together, these results demonstrate that 1-Br-Et was an efficient •OH-specific chromophore, affording dramatically enhanced NIR absorption and PA signals in response to •OH.

**Fig. 2:**
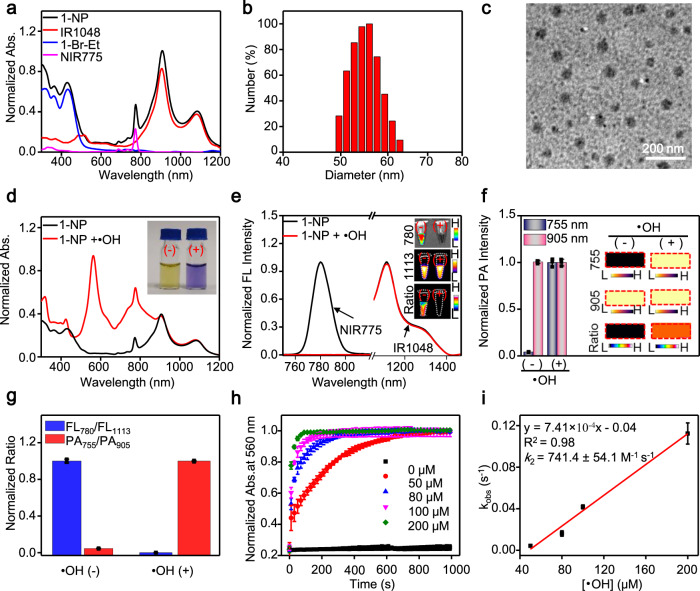
Characterization of 1-NP in vitro. **a** Comparison of the UV–vis-NIR absorption (abs.) spectra of 1-NP, IR1048, 1-Br-Et, and NIR775. **b** Dynamic light scattering (DLS), and **c** Transmission electron microscopy (TEM) image of 1-NP. The experiments in (**c**) were repeated independently three times with similar results. **d** Normalized UV–vis-NIR absorption and **e** normalized fluorescence (FL) spectra of 1-NP (56/1.65/20 μM 1-Br-Et/NIR775/IR1048) before and after incubation with •OH (200 µM Fe^2+^ + 1 mM H_2_O_2_) at r.t. for 3 min. Inset: FL images and ratiometric FL_780_/FL_1113_ images of 1-NP before (-) and after (+) incubation with •OH. FL spectra of NIR775 within 1-NP was acquired by synchronous FL scanning (*λ*_ex_ = 700–900 nm, offset **=** 10 nm). FL spectra of IR1048 within 1-NP was acquired upon excitation at 808 nm, and emission with bandpass between 900-1500 nm. **f** Normalized photoacoustic (PA) intensity and PA images along with ratiometric PA_755_/PA_905_ images (inset) of 1-NP (56/1.65/20 μM 1-Br-Et/NIR775/IR1048) before and after incubation with •OH (200 µM Fe^2+^ + 1 mM H_2_O_2_) at r.t. for 3 min. The PA images were acquired at 755 and 905 nm, respectively. **g** Normalized FL_780_/FL_1113_ and PA_755_ /PA_905_ of 1-NP upon incubation without and with •OH. **h** Normalized time-dependent increment of UV–vis absorption (560 nm) of 1-NP (8/0.24/2.86 μM 1-Br-Et/NIR775/IR1048) following incubation with varying concentrations of •OH (0, 50, 80, 100, 200 μM Fe^2+^ + 1 mM H_2_O_2_) at r.t.. **i** Plot of the pseudo-first-order rate (*k*_obs_) versus •OH concentration (50–200 µM) affords the second-order reaction rate (*k*_2_) between 1-NP and •OH at r.t. The *k*_obs_ was determined by fitting the absorption intensity with single exponential function of $y={y}_{0}\,+\,A\,\times {{{{{\rm{exp }}}}}}\,({R}_{0}\,\times \,t)$, where ${k}_{{{{{{\rm{obs}}}}}}}=-{R}_{0}$. The *k*_2_ value was obtained from the slope of the linear plot between *k*_obs_ and •OH concentration (*R*^2^ = 0.98). Data are presented as mean ± s.d. (*n* = 3 independent samples). Source data are provided as a Source Data file.

With 1-Br-Et, we next prepared the ratiometric NIR FL/PA bimodal imaging nanoprobe (1-NP) via DSPE-PEG_2000_-assisted encapsulation of 1-Br-Et, NIR775, and IR1048. NIR775 was selected as the NIR fluorophore due to its bright and stable FL emission at 780 nm, which can offer reliable signals for in vivo imaging^60,61^. Moreover, a good spectral overlap between its emission and 2-Br-Et’s absorption can elicit an efficient ET process, facilitating to quench its FL when 1-NP was converted into 2-NP (Supplementary Fig. 7a). IR1048 was chosen as the second NIR fluorophore due to (1) its FL emission at the NIR-II windows (*λ*_em_ = 1113 nm, Supplementary Fig. 7b), potentially improving resolution and sensitivity for in vivo imaging^33,62^; (2) little overlap of its 1113 nm FL with the absorption of 2-Br-Et at 767 nm (Supplementary Fig. 7b), avoiding to quench the NIR-II FL in the presence of •OH; (3) its high absorption at 905 and 1080 nm, allowing to offer strong PA signals (Supplementary Fig. 7c, d). More importantly, IR1048 was found to be inert to •OH and highly resistant to continuous light irradiation at 808 nm (Supplementary Fig. 8). These results suggest that IR1048 can not only offer sensitive “always on” NIR-II FL and PA signals for in vivo imaging, but also act as an efficient internal standard for self-calibration of FL and PA signals. To prepare 1-NP, the ratio of DSPE-PEG_2000_, 1-Br-Et, NIR775 and IR1048 was optimized to be 10/0.52/0.02/0.13 by mass (Supplementary Fig. 9), with the loading efficiency found to be nearly 100% (Supplementary Table 2). 1-NP displayed the characteristic absorption bands of 1-Br-Et, NIR775, and IR1048 (Fig. 2a). Dynamic light scattering (DLS) analysis showed that 1-NP could be well dispersed in an aqueous solution, with a mean hydrodynamic size of ~57.5 ± 3.5 nm and a polydispersity index of 0.229 ± 0.030 (Fig. 2b and Supplementary Table 2). The Zeta potential of 1-NP was measured to be −27.8 ± 2.8 mV, and transmission electron microscope (TEM) analysis revealed the appearance of spherical morphology (Fig. 2c).

### Response of 1-NP toward •OH

After adding •OH, the absorption of 1-NP at 560 and 767 nm increased rapidly, with a distinct color change from yellow to blue-violet (Fig. 2d). 1-NP initially displayed strong FL at both 780 nm and 1113 nm, yielding a relatively high FL_780_/FL_1113_ value and an obvious ratiometric FL image; after incubation with •OH, the FL of NIR775 at 780 nm decreased significantly, while the FL of IR1048 at 1113 nm was kept, resulting in a remarkable ~1026-fold decrement in FL_780_/FL_1113_ and a dark ratiometric FL image (Fig. 2e, g). To avoid the PA signal of NIR775 at 775 nm that may cause a high background prior to •OH treatment, the PA images of 1-NP were acquired at 755 nm (instead of 767 nm) and 905 nm, respectively. As shown in Fig. 2f, a dark PA image at 755 nm but a bright PA image at 905 nm appeared in 1-NP, resulting in a low PA_755_/PA_905_ value and a dark ratiometic PA image; after reaction with •OH, the PA signal at 755 nm was significantly elevated owing to the increments in absorption and photothermal effect (Supplementary Fig. 10), while the PA signal at 905 nm remained unchanged. Therefore, a bright ratiometic PA image appeared and the PA_755_/PA_905_ ratio significantly increased by ~22-fold (Fig. 2g), matching that of 1-Br-Et itself (Supplementary Fig. 6). These findings demonstrate the efficient response of 1-NP to •OH, affording a remarkable decrease in FL_780_/FL_1113_ but a concurrent increase in PA_755_/PA_905_. We subsequently measured the reaction kinetics between 1-NP and •OH, and *k*_2_ was found to be ~741.4 ± 54.1 M^−1^ s^−1^ (Fig. 2h, i), which was slightly lower than that of free 1-Br-Et, but comparable to or faster than other previously reported imaging probes for •OH (Supplementary Table 3). Considering the transient nature of endogenous •OH, such a large *k*_2_ could ensure an efficient reaction between 1-NP and •OH, facilitating to improve sensitivity for endogenous •OH.

**Fig. 3:**
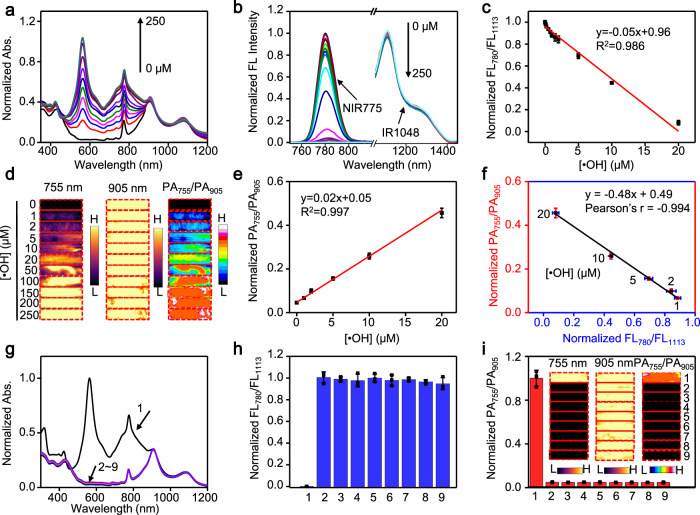
Measurement of •OH by 1-NP in vitro. **a** Normalized absorption and **b** nomalied fluorescence (FL) spectra of 1-NP upon incubation with different concentration •OH (0, 1, 2, 5, 10, 20, 50, 100, 150, 200, 250 μM Fe^2+^ + 1 mM H_2_O_2_) at r.t. for 3 min. **c** Plot of the normalized FL_780_/FL_1113_ values versus the concentration of •OH show a linear relationship ranging from 0.05-20 μM of •OH. The limit of detection (LOD) was determined to be ~3.69 nM by using the 3δ/k method. **d** Photoacoustic (PA) images at 755 and 905 nm, and ratiometric PA_755_/PA_905_ images of 1-NP (56/1.65/20 μΜ 1-Br-Et/NIR775/IR1048) upon incubation with the indicated concentration of •OH at r.t. for 3 min. **e** Plot of the normalized PA_755_/PA_905_ values versus the concentration of •OH show a linear relationship between 1 and 20 μM of •OH. The LOD was determined to be ~0.24 μM by using the 3δ/k method. **f** Plot of the normalized PA_755_/PA_905_ ratios versus the normalized FL_780_/FL_1113_ ratios shows a strong correlation (Pearson’s r = −0.994) between them upon incubation of 1-NP with the indicated concentration of •OH. **g** Normalized UV-Vis-NIR absorption spectra, **h** normalized FL_780_/FL_1113_ values and **i** normalized PA_755_/PA_905_ values of 1-NP after incubation with •OH and other representative reactive oxygen species (ROS) at r.t. for 24 h. Inset: PA images at 755 nm and 905 nm, and ratiometric PA_755_/PA_905_ images of 1-NP following indicated treatment. 1: •OH (200 µM Fe^2+^ + 1 mM H_2_O_2_); 2: ^1^O_2_ (1 mM H_2_O_2_ + 1 mM ClO^−^); 3: 300 µM t-BuOOH; 4: 1 mM ClO^−^; 5: 300 µM CuOOH; 6: 1 mM H_2_O_2_; 7: O_2_^·-^ (100 µM xanthine + 22 mU xanthine oxidase (XO)); 8: ONOO^−^ (1 mM NaNO_2_ + 1 mM H_2_O_2_); 9: Control. Data are presented as mean ± s.d. (*n* = 3 independent samples). Source data are provided as a Source Data file.

We then examined the sensitivity of 1-NP for the detection of •OH via ratiometric FL and PA imaging. Fig. 3a showed that the absorption of 1-NP between 500 and 900 nm increased with the concentration of •OH. The FL at 780 nm decreased accordingly, while the FL at 1113 nm was little changed (Fig. 3b). Plot of the resulting FL_780_/FL_1113_ versus the concentration of •OH showed a good linear correlation ranging from 0.05 to 20 µM, with a limit of detection (LOD) of ∼3.69 nM (3δ/k, Fig. 3c). The PA images of 1-NP at 755 nm, not at 905 nm, became brighter with the concentration of •OH (Fig. 3d and Supplementary Fig. 11), and the PA_755_/PA_905_ increased linearly versus •OH concentration from 1 to 20 μM, with a LOD of ∼0.24 μM (Fig. 3e). Notably, there is a good linearity between the normalized metrics of FL_780_/FL_1113_ and PA_755_/PA_905_ of 1-NP following incubation with •OH (1 to 20 μM), indicating a strong correlation between them (Pearson’s *r* = −0.994, Fig. 3f). Such a correlation suggests that both ratiometric FL and PA measurement are capable of reliably quantifying •OH, as they can verify the results each other. Owing to the high selectivity of 1-Br-Et toward •OH, 1-NP also displayed an excellent selectivity to detect •OH over other endogenous ROS (Fig. 3g–i and Supplementary Fig. 12). In addition, 1-NP appeared to be highly stable under physiological conditions (Supplementary Fig. 13). After reaction with •OH, the resulting 2-NP also showed high stability under physiologically relevant conditions (Supplementary Fig. 14), and the reaction between 1-NP and •OH was not reversible by intermittent addition and removal of •OH (Supplementary Fig. 15).

### Ratiometric FL/PA bimodality imaging of •OH in cells

**Fig. 4:**
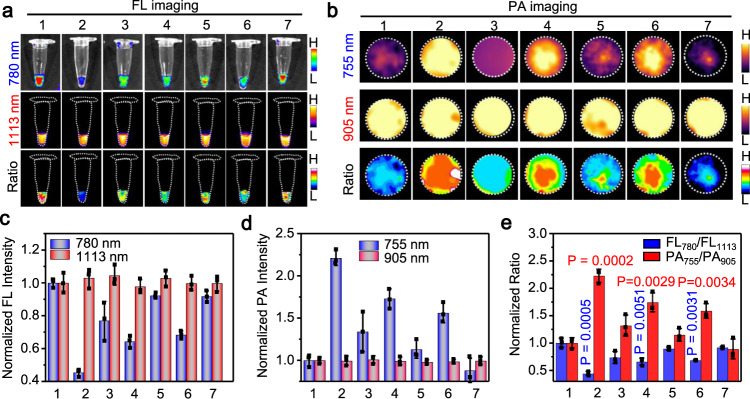
Ratiometric near-infrared fluorescence/photoacoustic imaging of intracellular •OH. **a** Fluorescence (FL) (780 and 1113 nm) and ratiometric FL_780_/FL_1113_ images, and **b** Photoacoustic (PA) images (755 and 905 nm) and ratiometric PA_755_/PA_905_ images of RAW264.7 cells (~5 × 10^5^ cells) upon treatment with 1-NP (56/1.65/20 μM 1-Br-Et/NIR775/IR1048) for 3 h plus different conditions. 1: Ctrl; 2: 200 μM Fe^2+^ + 1 mM H_2_O_2_; 3: 200 μM Fe^2+^ + 1 mM H_2_O_2_ + tempol (200 μM); 4: Lipopolysaccharide (LPS) (20 μg mL^−1^); 5: LPS (20 μg mL^−1^) + tempol (200 μM); 6: Phorbol 12-myristate 13-acetate (PMA) (20 μg mL^−1^); 7: PMA (20 μg mL^−1^) + tempol (200 μM). **c** Normalized FL and **d** PA intensities of RAW264.7 cells under indicated treatment. **e** Normalized FL_780_/FL_1113_ ratios and PA_755_/PA_905_ ratios of RAW264.7 cells under indicated treatment. Data are presented as mean ± s.d. (*n* = 3 independent cell pellets). Statistical differences were analyzed by Student’s two-sided *t*-test between Ctrl and indicated groups. Source data are provided as a Source Data file.

On account of the efficient response of 1-NP toward •OH in solution, we next investigated the ability for ratiometric FL/PA imaging of •OH in cells. Cell viability test revealed that 1-NP hold a good biocompatibility against RAW264.7 cells (Supplementary Fig. 16). We then employed 1-NP’s “always on” NIR-II fluorescence at 1113 nm and PA signal at 905 nm to optimize the incubation conditions. The FL and PA signals in RAW264.7 cell pellets both reached a plateau following incubation with 1-NP (containing 56/1.65/20 μM 1-Br-Et**/**NIR775/IR1048) for 3 h (Supplementary Figs. 17 and 18). Epifluorescence images showed that the nanoprobe was distributed mainly in the lysosomes after entry into RAW264.7 cells (Supplementary Fig. 19). With the optimized conditions, viable RAW264.7 cells incubated with 1-NP exhibited obvious NIR-II FL at 1113 nm and strong NIR-I FL at 780 nm (Fig. 4a). After incubation with the Fenton reagent (Fe^2+^ + H_2_O_2_) to produce •OH, the NIR-II FL at 1113 nm remained unchanged in the pellets, but the FL at 780 nm significantly decreased, consequently affording a dark ratiometric FL_780_/FL_1113_ image. The normalized FL_780_/FL_1113_ ratio (0.44 ± 0.04) was significantly ~2.3-fold lower than that of viable cells treated with 1-NP only (Fig. 4c, e). The subsequent addition of tempol, a reported •OH scavenger, to the Fenton reagent-pretreated cells could partially recover the 780 nm FL and increase FL_780_/FL_1113_ ratio to (0.74 ± 0.11). These results were consistent with the epifluorescence images of RAW264.7 cells following different treatments (Supplementary Fig. 20). Contrary to FL imaging, PA imaging of the same cell pellets showed a high PA signal at 905 nm but a low PA signal at 755 nm in the viable cells; upon treatment with the Fenton reagent, the PA signal remained constant at 905 nm while significantly increased at 755 nm, resulting in a brighter ratiometric PA_755_/PA_905_ image (Fig. 4b). The normalized PA_755_/PA_905_ ratio (2.23 ± 0.12) in Fenton reagent-treated cells was ~1.7-fold higher than that in tempol-treated  cells (1.32 ± 0.19) (Fig. 4d, e). These findings demonstrate that 1-NP was able to detect Fenton reagent-induced •OH production in RAW264.7 cells. 1-NP was further applied for the detection of endogenous •OH fluctuation in RAW264.7 cells upon stimulation with lipopolysaccharide (LPS)^63^ or phorbol 12-myristate 13-acetate (PMA)^64^, which showed obviously decreased FL at 780 nm but similarly strong NIR-II FL at 1113 nm in relative to unstimulated control cells (Fig. 4a, c). The resulting ratiometric FL_780_/FL_1113_ ratio in LPS- and PMA-stimulated cells were significantly ~1.52-fold (0.66 ± 0.06) and ~1.45-fold (0.69 ± 0.01) lower than that of control cells, respectively (Fig. 4e). PA images showed a significantly higher signal at 755 nm, not at 905 nm, in LPS- or PAM-stimulated cells compared to that of control cells (Fig. 4b, d). The PA_755_/PA_905_ ratio increased by ∼1.74-fold and ∼1.58-fold in LPS- and PAM-stimulated cells (Fig. 4e), respectively, which could be similarly prohibited by tempol. Therefore, 1-NP was also efficient for the detection of endogenous •OH production in RAW264.7 cells via ratiometric FL/PA imaging.

In addition to image •OH in cells, the ability of 1-NP for visualizing •OH in mice was then explored. Mice with subcutaneous (s.c.) injection of 1-NP displayed bright FL at both 780 nm and 1113 nm at the injection site, where bright PA image at 905 nm but not at 755 nm also appeared. In contrast, the FL at 780 nm, not at 1113 nm, was significantly reduced in mice with s.c. injection of 1-NP plus the Fenton reagent (Fe^2+^ + H_2_O_2_), with the FL_780_/FL_1113_ ratio decreased by ~5-fold. The PA signal at 755 nm, not at 905 nm, was enhanced accordingly, with the PA_755_/PA_905_ ratio increased by ~3.3-fold (Supplementary Figs. 21 and 22). These results imply that 1-NP could also be amenable for ratiometric FL/PA imaging of •OH production induced by Fenton reaction in vivo.

### Imaging of •OH in tumors during ferroptosis

It is recognized that the •OH levels are elevated in tumor cells undergoing ferroptosis, which induces lipid peroxidation and cell death^16^. Noninvasive imaging of •OH in tumors upon treatment with ferroptosis inducers could be beneficial to early report tumor ferroptosis and screen therapeutic outcomes in vivo. We employed the ratiometric FL/PA signals to noninvasively detect •OH in tumors during ferroptosis. To enhance targeted delivery and uptake into tumors, folic acid (FA) ligands were introduced onto the surface of 1-NP, yielding 1-NP-FA capable of binding to folate receptor (FR) overexpressed on tumor cells and eliciting FR-assisted active delivery (Supplementary Fig. 23). The mass ratio of FA, 1-Br-Et, NIR775, and IR1048 within 1-NP-FA was estimated to be 0.01: 0.52: 0.02: 0.13 (Supplementary Fig. 24 and Note 1). As with 1-NP, 1-NP-FA was also present as a mono-disperse NP, and showed excellent ratiometric FL/PA response toward •OH in vitro (Supplementary Fig. 25). Both 1-NP and 1-NP-FA displayed a good biocompatibility against murine breast 4T1 tumors (Supplementary Fig. 26). With the optimized conditions of 1-NP-FA for cellular study (Supplementary Fig. 27), 4T1 tumor cells incubated with 1-NP-FA exhibited significantly higher NIR-II FL than that with 1-NP, which could be inhibited when free FA was added prior to 1-NP-FA (Supplementary Fig. 28). When 1-NP-FA was i.v. injected into s.c. 4T1 tumor-bearing mice, whole-body in vivo FL imaging with the “always-on” NIR-II FL at 1113 nm showed that 1-NP-FA could gradually accumulate in 4T1 tumors (Supplementary Fig. 29). The tumor FL at 24 h was ~1.5-fold higher than that of 1-NP-treated tumor, indicating that 1-NP-FA was more preferentially to enter tumors via the FA-assisted active delivery. Biodistribution study revealed that 1-NP-FA was mainly accumulated in liver (%ID/g ≈ 28.5%) and tumor (%ID/g ≈ 17.1%) at 24 h post i.v. injection into s.c. 4T1 tumor-bearing mice (Supplementary Fig. 30), which was in agreement with ex vivo ratiometric FL/PA imaging of dissected tumors and main organs (e.g., lung, heart, kidneys, intestines, stomach, spleen) (Supplementary Fig. 31).

**Fig. 5:**
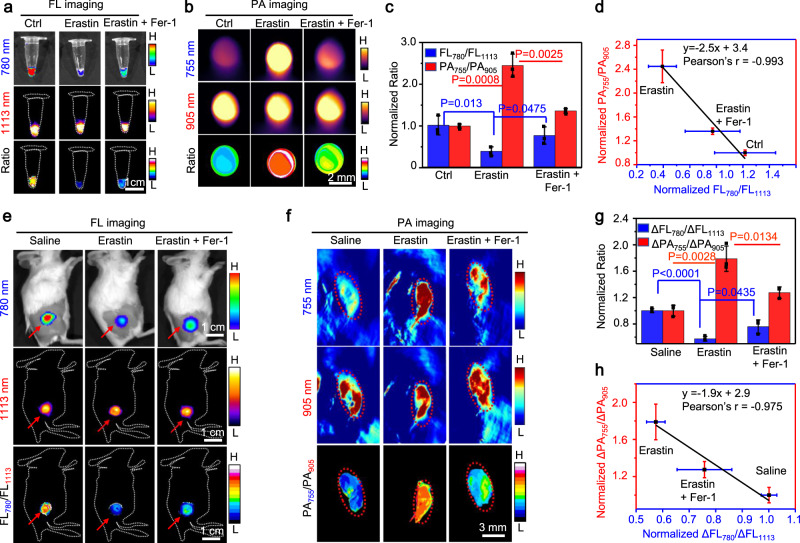
Ratiometric bimodal imaging of •OH in 4T1 tumors undergoing ferroptosis. **a** Fluorescence (FL) (780 and 1113 nm) and ratiometric FL_780_/FL_1113_ images, and **b** Photoacoustic (PA) images (755 and 905 nm) and ratiometric PA_755_/PA_905_ images of 4T1 cells upon incubation with 1-NP-FA (Ctrl), 1-NP-FA + erastin or 1-NP-FA + erastin + Fer-1. Cells were untreated (Ctrl), or pretreated with 10 μM erastin to induce ferroptosis, or 10 μM erastin plus 10 μM Fer-1 for 6 h, and then incubated with 1-NP-FA (56/1.65/20 μM 1-Br-Et/NIR775/IR1048) for another 2 h. **c** Normalized FL_780_/FL_1113_ and PA_755_/PA_905_ ratios of 4T1 cells treated with indicated conditions in a and b**. d** Plot of the FL_780_/FL_1113_ ratios versus PA_755_/PA_905_ ratios shows a strong correlation (*r* = −0.993) between them in 4T1 cells after indicated treatment. **e** FL and ratiometric FL_780_/FL_1113_ images, and **f** PA images and ratiometric PA_755_/PA_905_ images of s.c. 4T1 tumors upon treatment with saline, erastin or erastin plus Fer-1, followed by intravenous (i.v.) injection of 1-NP-FA. Mice with subcutaneous (s.c.) 4T1 tumors were intraperitoneal (i.p.) injected with saline, erastin (20 mg kg^−1^) or erastin (20 mg kg^−1^) plus Fer-1 (20 mg kg^−1^), and 12 h later, 1-NP-FA (1.68/0.05/0.6 mM 1-Br-Et/NIR775/IR1048, 200 μL) were i.v. injected into mice. After 24 h, the FL and PA images were acquired. Red arrows in (**e**) and red circles in (**f**) indicate the tumor locations. **g** Normalized ΔFL_780_/ΔFL_1113_ and ΔPA_755_/ΔPA_905_ ratios in 4T1 tumors following indicated treatment. **h** Plot of the ΔFL_780_/ΔFL_1113_ ratios versus ΔPA_755_/ΔPA_905_ ratios show a strong correlation (*r* = −0.975) between them in 4T1 tumors following indicated treatment. Data are presented as mean ± s.d. (*n* = 3 independent cell pellets or mice). Statistical differences were analyzed by Student’s two-sided *t*-test. Source data are provided as a Source Data file.

To apply 1-NP-FA for the monitoring of endogenous •OH, 4T1 cells were firstly induced ferroptosis with erastin, a membrane-permeable antitumor agent capable of inducing ferroptosis and increasing •OH production in tumor cells^13^ (Supplementary Figs. 32 and 33). The subsequent incubation of erastin-treated 4T1 cells with 1-NP-FA showed that the NIR-II FL at 1113 nm remained as strong as that of erastin-untreated viable cells, but the NIR-I fluorescence at 780 nm decreased noticeably owing to the oxidation of 1-Br-Et by elevated •OH. As such, the ratiometric FL_780_/FL_1113_ FL image became much darker in the erastin-treated ferroptotic cells; the FL_780_/FL_1113_ ratio (0.40 ± 0.11) was significantly ~2.5-fold lower than that of erastin-free cells (1 ± 0.23). When ferrostatin-1 (Fer-1), a ferroptosis inhibitor^65^, was added into the erastin-treated 4T1 cells to suppress ferroptosis and attenuate •OH production, the FL at 780 nm was recovered, yielding a brighter ratiometric FL_780_/FL_1113_ FL image and increased FL_780_/FL_1113_ ratio to (0.77 ± 0.21) (Fig. 5a, c and Supplementary Fig. 34a). Contrary to ratiomatric FL signal, the PA signal at 755 nm was low in viable cells, which was significantly enhanced in 4T1 cells after induction of ferroptosis by erastin. The ratiometric PA_755_/PA_905_ image became brighter, and the ratio (2.45 ± 0.27) was significantly ~2.45-fold higher than that of viable 4T1 cells (1 ± 0.05); the addition of Fer-1 to the erastin-treated cells could dramatically lower the PA_755_/PA_905_ ratio (1.36 ± 0.05) (Fig. 5b, c and Supplementary Fig. 34b). Note that a good correlation (*r* = −0.993) between the normalized FL_780_/FL_1113_ and PA_755_/PA_905_ in 4T1 cells under different treatment was observed (Fig. 5d). These findings indicate that 1-NP-FA was an efficient ratiometric FL/PA bimodal probe capable of feasibly reporting endogneous •OH production in tumor cells during ferroptosis.

We next applied 1-NP-FA for noninvasive imaging of •OH production in tumors following erastin-induced ferroptosis in vivo. Mice bearing s.c. 4T1 tumors were induced ferroptosis with i.p. injection of erastin, where the elevated •OH levels were firstly validated by FL staining of resected tumor tissue slices with hydroxyphenyl fluorescein (Supplementary Fig. 35). 1-NP-FA was then i.v. injected into saline-treated, erastin-treated or erastin plus Fer-1-treated mice, and the resulting FL and PA images were acquired at 0, 4, 12, 24, and 36 h (Supplementary Fig. 36a). As the background signals (at 0 h) were low while 1-NP-FA possessed strong “always-on” NIR-II FL and PA signals, the delivery of 1-NP-FA into tumors in living mice could be monitored via NIR-II FL and PA imaging (Supplementary Figs. 36b and 37). Both the NIR-II FL at 1113 nm and PA signals at 905 nm were similarly strong in these three groups of tumors following different treatment, indicating that the delivery and accumulation of 1-NP-FA among these tumors was little different (Fig. 5e, f and Supplementary Fig. 38). However, owing to the upregulated •OH levels in erastin-induced ferroptotic tumors, the NIR-I FL at 780 nm was obviously reduced, while the PA signal at 755 nm was significantly enhanced in the erastin-treated tumors. By dividing the signal enhancement in FL_780_ (ΔFL_780_) to that in FL_1113_ (ΔFL_1113_) and in PA_755_ (ΔPA_755_) to that in PA_905_ (ΔPA_905_), respectively, the normalized ΔFL_780_/ΔFL_1113_ declined from 1 to 0.57 ± 0.04, whereas the normalized ΔPA_755_/ΔPA_905_ increased from 1 to 1.79 ± 0.19 (Fig. 5g). On the basis of computed FL and PA metrics, erastin-treated tumors display a much darker ratiometric FL image but a brighter ratiometric PA image compared to that of saline-treated tumors (Fig. 5e, f). The subsequent i.p. injection of Fer-1 to inhibit tumor ferroptosis could recover the NIR-I FL and lower the PA signals in erastin-treated tumors, with the normalized ΔFL_780_/ΔFL_1113_ increased to 0.76 ± 0.10 and the normalized ΔPA_755_/ΔPA_905_ declined to 1.27 ± 0.09, implying that Fer-1 could suppress •OH production in erastin-treated tumors (Fig. 5g). By virtue of the deep tissue penetration ability offered by ratiometric PA imaging in NIR regions (Supplementary Fig. 39), the concentrations of •OH in tumors were then quantified (Supplementary Figs. 38d, e). The •OH concentration in saline-treated 4T1 tumors was estimated to be 14.5 ± 1.51 μM, which significantly increased to 28.8 ± 3.49 μM in the erastin-treated ferroptotic tumors; the addition of Fer-1 could significantly lower the tumor •OH concentration to 19.4 ± 1.59 μM. These results demonstrate that the •OH concentration was significantly upregulated in erastin-induced ferroptotic tumor, which could be prohibited by Fer-1. As with cellular imaging, there was also a strong correlation between the normalized ΔFL_780_/ΔFL_1113_ and ΔPA_755_/ΔPA_905_ in these three groups of 4T1 tumors (Fig. 5h), indicating that ratiometric FL and PA signals are capable of verifying each other in imaging of •OH production in tumors during ferroptosis. Therefore, 1-NP-FA could provide reliable ratiometric FL/PA signals for the detection of •OH levels, facilitating to monitor tumor ferroptosis.

### Imaging of tumor •OH production upon radiotherapy

We further applied 1-NP-FA for ratiometic FL/PA imaging of •OH production in 4T1 tumors during RT, as mounting evident demonstrates that effective RT generally involves the production of endogenous •OH to kill tumor cells^66^. 4T1 cells were incubated with 1-NP-FA for 2 h, and then treated with X-ray radiation (5 Gy). 4T1 cells upon RT showed that the intracellular NIR775’s FL at 780 nm was significantly lower than that of nonradiated cells, while the IR1048’s FL at 1113 nm remained bright in the cells (Supplementary Fig. 40a, c). The FL_780_/FL_1113_ ratio (0.40 ± 0.08) was reduced by ~2.5-fold relative to that of nonradiated cells (1 ± 0.15, Supplementary Fig. 40e). The PA signal at 755 nm in the same RT cells was switched on (Supplementary Fig. 40b, d), and the resulting PA_755_/PA_905_ ratio (2.72 ± 0.22) remarkably increased by ~2.7-fold (Supplementary Fig. 40e), matching that of reduced FL_780_/FL_1113_ metric. The addition of tempol into the 4T1 cells prior to X-ray irradiation could scavenge the endogenous •OH, yielding a high FL_780_/FL_1113_ ratio (0.65 ± 0.13) but a low PA_755_/PA_905_ ratio (1.61 ± 0.24). The subsequent examination of cell viability showed obvious cell death in 4T1 cells receiving 5 Gy X-ray irradiation at 48 and 72 h as compared with the non-irradiated cells (Ctrl), and 1-NP-FA did not cause obvious interference to X-ray (5 Gy) irradiation-induced cell death (Supplementary Fig. 41). These results suggest that 1-NP-FA could efficiently tell the increased •OH level in 4T1 cells after X-ray irradiation, potentially allowing to predict the RT effect against tumor cells.

**Fig. 6:**
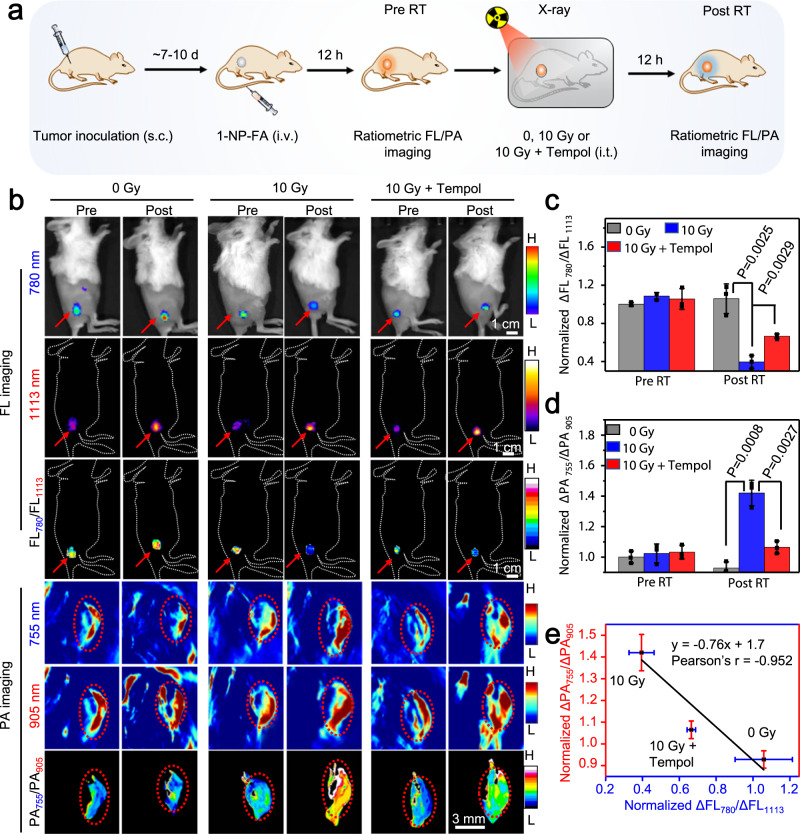
Ratiometric bimodal imaging of •OH production in 4T1 tumors undergoing X-ray RT. **a** Schematic for noninvasive ratiometric fluorescence/photoacoustic (FL/PA) imaging of •OH in 4T1 tumor-bearing mice upon radiotherapy (RT) with X-ray. 4T1 tumor-bearing mice were intravenous (i.v.) injected with 1-NP-FA (1.68/0.05/0.6 mM 1-Br-Et/NIR775/IR1048, 200 μL). After 12 h, the FL and PA images were acquired (Pre RT). The tumors were then unirradiated (0 Gy) or irradiated with X-ray (10 Gy, 1.0 Gy min^−1^ for 10 min); to inhibit tumor •OH, the mice were intratumorly (i.t.) injected with tempol (50 mg kg^−1^), and then irradiated with X-ray (10 Gy). After another 12 h, the FL and PA images were acquired (Post RT). **b** FL, PA, and corresponding ratiometric FL or PA images of 4T1 tumors following indicated treatment. Red arrows and circles indicate the tumor locations. **c** Normalized ΔFL_780_/ΔFL_1113_ ratios and **d** normalized ΔPA_755_/ΔPA_905_ ratios in 4T1 tumors following indicated treatment**. e** Plot of the normalized ΔFL_780_/ΔFL_1113_ ratios versus normalized ΔPA_755_/ΔPA_905_ ratios shows a good correlation (*r* = −0.952) between them in 4T1 tumors upon indicated treatment. Data are presented as mean ± s.d. (*n* = 3 independent mice). Statistical differences were analyzed by Student’s two-sided *t*-test. Source data are provided as a Source Data file.

To noninvasively image •OH production in tumors response to radiotherapy, s.c. 4T1 tumor-bearing mice were i.v. injected with 1-NP-FA and after 12 h, the tumors were unirradiated (0 Gy), irradiated with X-ray (10 Gy) or irradiated with X-ray (10 Gy) plus intratumoral (i.t.) injection of tempol. The raitometric FL and PA images were acquired before (Pre RT) and 12 h after irradiation (Post RT) (Fig. 6a). Prior to RT, the tumor FL at 780 and 1113 nm, and PA signals at 755 and 905 nm were acquired, showing nearly identical among all three groups of mice; thereby the tumors displayed a similarly high ΔFL_780_/ΔFL_1113_ but a low ΔPA_755_/ΔPA_905_ value (Fig. 6b–d and Supplementary Fig. 42). After RT, owing to the continuous uptake of 1-NP-FA, the ΔFL at 780 and 1113 nm both increased in the unirradiated tumors receiving 0 Gy X-ray. However, as the tumor ratiometric FL signal is independent on 1-NP-FA’s concentration, the normalized ΔFL_780_/ΔFL_1113_ ratio (1.06 ± 0.16) was little different from that in the Pre RT tumors (1.0 ± 0.02), which was also observed in the ratiometric ΔPA_755_/ΔPA_905_ signal. By contrast, in the tumors receiving 10 Gy X-ray RT, though the tumor ΔFL at 1113 nm was nearly the same as that of 0 Gy X-ray-treated tumors, the ΔFL at 780 nm was significantly reduced, and the normalized ΔFL_780_/ΔFL_1113_ (0.40 ± 0.07) declined by ~2.7-fold, which was prohibited when tempol was i.t. injected to scavenge •OH. These results indicate the elevated •OH levels in the 4T1 tumor response to 10 Gy X-ray RT, which was further confirmed by ratiometric PA imaging of the same mice. It was found that the ΔPA signal at 755 nm in the 10 Gy X-ray RT tumors was much higher than that in tumors Pre RT, nonirradiated tumors (0 Gy), or tumors receiving 10 Gy X-ray RT plus tempol. The normalized ΔPA_755_/ΔPA_905_ value was significantly ~1.4-fold higher in the 10 Gy X-ray RT tumors (1.42 ± 0.08) compared to that in the same tumors prior to RT (1.02 ± 0.06) or in the nonirradiated tumors (0.93 ± 0.04). Notably, plot of the normalized ΔFL_780_/ΔFL_1113_ versus the normalized ΔPA_755_/ΔPA_905_ in these three groups of tumors following different treatment showed a strong correlation between them (Fig. 6e). Such a strong correlation together with the probe’s concentration-independent feature of ratiometric FL/PA imaging could facilitate to overcome potential false-positive signals in tumors, allowing to improve accuracy for in vivo imaging of tumor •OH. We then quantified the •OH concentration in s.c. tumors prior to and RT with 0 Gy, 10 Gy X-ray or 10 Gy X-ray plus tempol according to the working curve of ratiometric PA imaging (Supplementary Fig. 38d). In the tumors receiving 0 Gy X-ray RT, the •OH concentration was estimated to be 16.8 ± 0.88 μM (Supplementary Fig. 42f), close to that in the same tumors Pre RT. However, in the tumors receiving 10 Gy X-ray RT, the •OH concentration obviously increased to 27.7 ± 1.84 μM, which dropped to 19.8 ± 0.89 μM when tempol was i.t. in tumors prior to 10 Gy X-ray RT. These results revealed that •OH concentration was also significantly upregulated in tumors following RT with 10 Gy X-ray. After imaging, we further monitored the tumor size and body weight of each group of mice (Supplementary Fig. 43). Mice treated with saline plus X-ray or 1-NP-FA plus X-ray showed similarly slow tumor growth rates, and the average tumor sizes at day 13 were significantly smaller compared to that of the control mice treated with saline alone, suggesting that (1) 10 Gy X-ray was effective in inhibiting tumor growth and (2) 1-NP-FA did not perturb the RT effectiveness in inhibiting tumor growth. 1-NP-FA hold the potential to quantify •OH concentration in RT tumors, which might be amenable for the early prediction of tumors response to RT.

The in vivo biosafety of 1-NP-FA was then evaluated. First, hemolysis examination showed that the hemolytic ratio of 1-NP-FA against mouse red blood cells was <2 %, indicating that 1-NP-FA showed no obvious hemolysis (Supplementary Figs. 44a, b). Second, we found that the average body weights were little different between saline-treated and 1-NP-FA-treated mice over the time course of 28 days (Supplementary Fig. 44c). Third, the blood biochemistry and blood count tests showed that the levels of the biochemistry and hematological markers were all within the reference ranges, suggesting that 1-NP-FA did not elicit obvious inflammation or immune response to mice (Supplementary Figs. 44d, e). Finally, histological analysis of resected major organs showed normal morphologies in both 1-NP-FA-treated and saline-treated groups (Supplementary Fig. 45). In all, these results suggest that 1-NP-FA hold high biocompatibility, which was a safe molecular probe for in vivo imaging.

## Discussion

Ratiometric imaging probes that allow built-in self-calibration for the correction of analyte-independent factors have shown promise for in vivo imaging. Particularly, ratiometric NIR FL/PA bimodal probes leverage the high sensitivity offered by NIR FL along with the high spatial-resolution (up to micrometers) and deep tissue-penetration (up to centimeters) offered by PA imaging, which can offer complementary advantages to enable refined in vivo imaging of biomolecules. Recently, a few ratiometric FL/PA bimodal imaging probes have been reported for in vivo imaging of H_2_S, nitroreductase, granzyme B, pH or ATP/pH (Supplementary Table 4). However, there is still lack of •OH-responsive ratiometric NIR FL/PA probe for in vivo imaging •OH. Our work started from structural modification of reduced organic π-electron diene EMs, and optimized EM 1-Br-Et to be a unique •OH-responsive NIR chromophore. EM 1-Br-Et hold fastest reaction kinetics (1140 ± 53.4 M^−1^ s^−1^) toward •OH, which could be rapidly and specifically oxidized into dication (2-Br-Et) by •OH, accompanying by a distinct UV-vis-NIR absorption shift from 436 to 767 nm. Such a unique response chemistry could allow us to design the first example of •OH-responsive ratiomertic NIR FL/PA bimodal imaging probe (1-NP-FA) for sensitive imaging of tumor •OH in living mice. We have demonstrated that 1-NP-FA could predominantly accumulate in s.c. tumor following intravenous injection, and produce complementary ratiometric FL_780_/FL_1113_ and PA_755_/PA_905_ imaging signals to monitor tumor •OH production in response to radiotherapy or ferroptosis.

In comparison with existing optical imaging probes for •OH (Supplementary Table 3), 1-NP-FA offers several advantages: (1) 1-NP-FA holds fast reaction kinetics toward •OH, rendering it suitable to measure the endogenous •OH that generally shows short lifetime and small diffusion distance in biology; (2) 1-NP-FA displays large FL_780_/FL_1113_ (~1026-fold) and PA_755_/PA_905_ (~22-fold) turn-on ratios in response to •OH, ensuring high sensitivity to detect •OH; (3) 1-NP-FA shows good selectivity to •OH over other ROS; (4) 1-NP-FA uses not only ratiometric NIR FL imaging that possesses high sensitivity, but also ratiometric PA imaging that features with improved spatial-resolution and penetration depth over FL imaging for in vivo imaging of •OH; (5) 1-NP-FA holds high biocompatibility and accumulates predominantly in tumors after systemic administration, which is suited to imaging tumor •OH; (6) 1-NP-FA does not perturb the radiotherapy effectiveness in tumors, particularly suitable to imaging tumor •OH production during radiotherapy; (7) 1-NP-FA allows to quantitatively measure tumor •OH levels in vivo via complementary ratiometric FL_780_/FL_1113_ and PA_755_/PA_905_ imaging, revealing significantly upregulated •OH levels in s.c. 4T1 tumors upon erastin-induced ferroptosis (Supplementary Fig. 38) or 10 Gy X-ray RT (Supplementary Fig. 42).

RT has been applied for treating over 50% cancer patients in clinics. It is conceived that the •OH production via radiolysis of water molecules plays key roles in breaking DNA and killing cancer cells in a standard RT. Accurate detection of •OH levels in tumors during RT can offer useful information to enable early evaluation of RT response, but has not yet been achieved, likely owing to the lack of reliable molecular probes toward tumor •OH in vivo. Via noninvasive ratiometric NIR FL/PA bimodal imaging offered by 1-NP-FA, we provided direct and quantitative measurement of •OH levels in s.c. 4T1 tumors during RT, revealing that the •OH levels are significantly upregulated in the s.c. 4T1 tumors at 12 h post 10 Gy X-ray RT (Fig. 6 and Supplementary Fig. 42). Note that the increased tumor •OH concentrations upon RT (10 Gy) could efficiently inhibit tumor growth in living mice (Supplementary Fig. 43), protruding the potential of 1-NP-FA for early prediction of tumors response to RT, which may benefit to improve treatment planning and prognosis. Considering that tumor heterogeneity and microenvironment (e.g., hypoxia) can cause heterogeneous RT response^9^, further investigation of 1-NP-FA in large sample sizes and different tumor models are needed. In addition, 1-NP-FA is designed to have a layer of FA ligands on the surface, which is particularly effective in imaging of •OH in the FR-positive tumors. In the future, surface medication with other ligands to allow active delivery of nanoprobe into other type of tumors for improving tumor •OH detection are needed as well.

In conclusion, we developed a ratiometric NIR FL/PA bimodality imaging probe (1-NP) via doping an optimized •OH-specific chromophore (1-Br-Et) into NIR775 and IR1048 containing micellar NPs. As the absorption of 1-Br-Et is below 500 nm, 1-NP initially showed an ‘on’ NIR-I FL of NIR775 at 780 nm and an ‘always on’ NIR-II FL of IR1048 at 1113 nm, while an ‘off’ PA signal of 1-Br-Et at 755 nm and an ‘always on’ PA signal of IR1048 at 905 nm, thus displaying a relatively high FL_780_/FL_1113_ but a low PA_755_/PA_905_ ratio. Upon reaction with •OH, the light yellow 1-Br-Et within 1-NP could be rapidly oxidized into purple 2-Br-Et, resulting in a noticeable bathochromic shift of UV-vis absorption to above 750 nm; the PA signal at 755 nm was switched on but the NIR-I FL at 780 nm was dramatically quenched likely due to the ET from NIR775 to the formed 2-Br-Et, thereby resulting in a significant ~22-fold increment in PA_755_/PA_905_ ratio and concurrently a remarkable ~1026-fold decrement in FL_780_/FL_1113_ ratio. We demonstrated that 1-NP hold high sensitivity and specificity toward •OH, which could readily detect •OH fluctuation in LPS- or PMA-stimulated RAW264.7 cells via the ratiometric FL/PA bimodality imaging. We further prepared a tumor-targeting nanoprobe (1-NP-FA) that showed enhanced accumulation in the s.c. 4T1 tumors over 1-NP after i.v. injection, which could be unambiguously monitored by FL/PA imaging with the ‘always on’ NIR-II FL at 1113 nm and PA signal at 905 nm. Using the distinct switch in FL_780_/FL_1113_ and PA_755_/PA_905_ ratios in response to •OH, 1-NP-FA could permit noninvasive and real-time bimodality imaging of •OH in vivo, validating an elevated •OH levels in 4T1 tumors following erastin-induced ferroptosis or X-ray-triggered RT. It was notable that a strong correlation between the resulting FL_780_/FL_1113_ ratio and PA_755_/PA_905_ ratio was observed in tumors with different •OH levels. This could allow 1-NP-FA to mutually verify the obtained imaging results, facilitating to differentiate the •OH-induced signal change from other false-positive signals in vivo. Moreover, as ratiometric imaging could provide a built-in self-calibration effect capable of correcting analyte-independent factors in dynamic biological environments^67,68^, 1-NP-FA might pose improved accuracy over traditional activatable fluorescent probes for the detection of •OH in vivo. By virtue of complementary advantages offered by ratiometric NIR FL and PA imaging, 1-NP-FA could enable the in vivo imaging of tumor •OH with high sensitivity, good spatial-resolutions and improved penetration depth. This may provide a useful tool for the early monitoring of tumor response to RT or other •OH-related therapeutic approaches (e.g., chemodynamic therapy, ferroptosis), allowing for the prediction of therapeutic efficacy of an applied therapy and optimization of therapeutic intervention.

## Methods

The synthesis of 1-Br-Et was depicted in Supplementary Fig. 46 and Supplementary methods. The NMR spectra and single crystal analysis of 1-Br-Et were shown in Supplementary Figs. 47–53.

### Preparation of 1-NP and 1-NP-FA

All the nanoprobes were prepared using amphiphilic polymers-assisted nanoprecipitation method. Typically, to prepare 1-NP, 1-Br-Et (0.52 mg) was dissolved in dimethyl sulfoxide (DMSO, 0.25 mL), and NIR775 (0.02 mg), DSPE-PEG_2000_ (10 mg) and IR1048 (0.13 mg) were dissolved in tetrahydrofuran (THF, 0.5 mL) to form a homogeneous solution. These two solutions were rapidly injected into deionized (D.I.) water (9 mL) under continuous sonication. After addition, the mixture was kept sonication in an ice bath for 10 min. Then, THF was removed under vacuum. The resulting aqueous solution was transferred to a centrifugal filter (Millipore, 10 KDa), washed with D.I. water under centrifugation (2040 × *g*) to remove DMSO and free compounds. The stock solution of 1-NP in D.I. water was obtained after centrifugation, and the amount of 1-Br-Et, IR1048, and NIR775 within 1-NP were determined by measuring the UV-vis-NIR absorption spectra. To prepare 1-NP-FA, a mixture of DSPE-PEG_2000_/DSPE-PEG_2000_-FA (9.9/0.1 mg) were used instead of DSPE-PEG_2000_.

### Cell culture

RAW264.7 macrophages and mouse breast cancer cell line (4T1 cells) were purchased from Stem Cell Bank, Chinese Academy of Sciences (Shanghai, China). All cell lines were routinely tested for mycoplasma contamination, and cells were authenticated by Short Tandem Repeat test. RAW264.7 macrophages and 4T1 cells were cultured in DMEM (Dulbecco’s Modified Eagle Medium) medium supplemented with fetal bovine serum (FBS, 10%) and antibiotics (100 units mL^−1^ penicillin and 100 units mL^−1^ streptomycin). All cells were cultured in a humidified environment containing 5% CO_2_ and 95% air at 37 °C.

### Ratiometric FL/PA bimodal imaging of •OH in cells

RAW264.7 or 4T1 cells (5 × 10^5^ cells well^−1^) were plated at culture dish. Cells were then incubated with various concentration of 1-NP or 1-NP-FA (1.25, 2.5, 5, 10, 20, and 30 μM based on concentration of IR1048) at 37 °C for different time (0.5, 1, 2, 3 and 6 h). To image the baseline of •OH levels in RAW264.7 cells, cells were incubated with 1-NP (56/1.65/20 μM 1-Br-Et/NIR775/IR1048) for 3 h (Ctrl); To image the exogenous •OH levels in RAW264.7 cells, cells were incubated with 1-NP (56/1.65/20 μM 1-Br-Et/NIR775/IR1048) for 3 h, and the medium was discarded, washed with cold PBS (1×, pH 7.4) twice, following by incubation in the DMEM medium containing 200 μM Fe^2+^ together with 1 mM H_2_O_2_ for 30 min. To image the •OH levels in RAW264.7 cells treated with •OH scavenger tempol, cells were incubated with 1-NP (56/1.65/20 μM 1-Br-Et /NIR775/IR1048) for 3 h, and the medium was discarded, washed with cold PBS (1×, pH 7.4) twice, and then cells were incubated with 200 μM Fe^2+^ and 1 mM H_2_O_2_ in the presence of 200 μM tempol for 30 min. To elevate the endogenous •OH production, RAW264.7 cells were incubated with LPS (20 μg mL^−1^) or PMA (20 μg mL^−1^) for 3 h, and then incubated with 1-NP (56/1.65/20 μM 1-Br-Et/NIR775/IR1048) for another 3 h. To inhibit the •OH levels in LPS- or PMA-stimulated RAW264.7 cells, cells were pretreated with LPS (20 μg mL^−1^) or PMA (20 μg mL^−1^) plus tempol (200 μM) for 3 h. Then, cells were incubated with 1-NP (56/1.65/20 μM 1-Br-Et/NIR775/IR1048) for another 3 h.

To image •OH levels of 4T1 cells, cells were incubated with 1-NP-FA (56/1.65/20 μM 1-Br-Et/NIR775/IR1048) (Ctrl) for 2 h. To image •OH levels in 4T1 cells undergoing induced ferroptosis, cells were pretreated with erastin (10 μM) for 6 h, and then incubated with 1-NP-FA (56/1.65/20 μM 1-Br-Et/NIR775/IR1048) for another 2 h. To suppress •OH levels in the erastin-induced ferroptotic 4T1 cells, cells were pretreated with erastin (10 μM) plus ferroptosis inhibitor Fer-1 (10 μM) for 6 h, and then incubated with 1-NP-FA (56/1.65/20 μM 1-Br-Et/NIR775/IR1048) for another 2 h. To image the •OH levels in 4T1 cells after irradiation with X-ray, 4T1 cells were incubated with 1-NP-FA (56/1.65/20 μM 1-Br-Et/NIR775/IR1048) for 2 h, and then the medium was discarded, washed with PBS (1×, pH 7.4), following by irradiation with X-ray (0 or 5 Gy, 1.0 Gy min^−1^). To inhibit the •OH levels in 4T1 cells upon irradiation with X-ray, cells were incubated with 1-NP-FA (56/1.65/20 μM 1-Br-Et/NIR775/IR1048) for 2 h, and then the medium was discarded, washed with PBS (1×, pH 7.4). Tempol (200 μM) was added, and the cells were irradiated with X-ray (5 Gy, 1.0 Gy min^−1^).

Prior to FL and PA imaging, the above-mentioned cells were washed twice with PBS (1×, pH 7.4), and trypsin was added to detach the cells. The cell pellets were collected and centrifuged at 4 °C (161 × *g*, 4 min). FL images of NIR775 and IR1048 in the cell pellets were measured on the IVIS Lumina XR III system and NIR-OPTICS Series III 900/1700 whole animal imaging system (NIR775: *λ*_ex _= 740 nm; IR1048: *λ*_ex _= 808 nm). The FL intensities in the cell pellets were quantified using region of the interest (ROI) over the images in the Living Image Software (4.5.2, PerkinElmer, MA, U.S.A) and the ImageJ software (NIH). PA images and intensities at 755 and 905 nm were collected and analyzed on the Endra Nexus128 PA tomography system for RAW264.7 cells, and the multispectral optoacoustic tomography (MSOT) system for 4T1 cells. The corresponding FL_780_/FL_1113_ and PA_755_/PA_905_ values in the pellets were calculated, and applied for the preparation of ratiometric images using the ImageJ software according to the procedure described in the Supplementary Methods.

### Animals and tumor models

BALB/c female mice (~6–8-weeks old, body weight: ~20 g) were purchased from the Model Animal Research Center (MARC) of Nanjing University. Mice were housed with free access to food and water in an ambient temperature-controlled (23 ± 3 °C) room with 12 h dark-light cycles and 40–70% humidity. All animal experiments were approved by the Institutional Animal Care and Use Committee (IACUC) of Nanjing University. To establish mouse model with s.c. tumors, 4T1 cells (2.0 × 10^6^ cells, 50 μL) suspended in PBS were injected subcutaneously onto the right rear flanks of each mouse. FL and PA imaging were conducted when the tumor volume reached about 60–130 mm^3^.

### Ratiometric FL/PA imaging of tumor •OH in vivo

To noninvasively monitor •OH production in erastin-induced ferroptotic 4T1 tumors, 4T1 tumor-bearing mice were i.p. injected with saline (0.9%), erastin (20 mg kg^−1^) or erastin (20 mg kg^−1^) together with Fer-1 (20 mg kg^−1^). After 12 h, mice were i.v. injected with 1-NP-FA (1.68/0.05/0.6 mM 1-Br-Et/NIR775/IR1048, 200 μL) for 0, 4, 12, 24, 36 h. To noninvasively monitor •OH generation in tumors receiving X-ray irradiation, 4T1 tumor-bearing mice were i.v. injected with 1-NP-FA (1.68/0.05/0.6 mM 1-Br-Et/NIR775/IR1048, 200 μL). After 12 h, the FL and PA images were acquired (Pre RT), and then the mice were irradiated by X-ray (0 or 10 Gy, 1.0 Gy min^−1^) for 10 min. In order to inhibit •OH generation in the RT tumors, tempol (50 mg kg^−1^) was directly injected into tumors, and the tumors were then irradiated for 10 min by X-ray (10 Gy, 1.0 Gy min^−1^). After12 h, the FL and PA images were recorded. FL images of NIR775 and IR1048 of mice were acquired on the IVIS Lumina XR III system (*λ*_ex _= 740 nm) and a home-built imaging set-up (CDD: NIRvana TE 640) (IR1048: *λ*_ex _= 808 nm). The FL intensities were quantified using region of the interest (ROI) over the images in the Living Image Software for NIR775 and the ImageJ software (NIH) for IR1048. PA images and intensities of tumors at 755 and 905 nm were collected and analyzed on the multispectral optoacoustic tomography (MSOT) system. Ratiometric FL_780_/FL_1113_ and PA_755_/PA_905_ tumor images were generated using the ImageJ software according to the procedure described in the Supplementary Methods.

### Statistical analysis

Results are expressed as mean ± s.d. unless stated otherwise. Student’s *t* tests were used to evaluate statistical significance between groups using GraphPad Prism 6 including assumptions of tests used (GraphPad Software Inc., CA, USA). *P* < 0.05 was statistically significant.

### Reporting summary

Further information on research design is available in the Nature Research Reporting Summary linked to this article.

## Supplementary information

Supplementary Information

Description of Additional Supplementary Files

Supplementary Data 1

Reporting Summary

## Data Availability

The experimental data supporting the findings of this study are available within the article and Supplementary Information. The data for all graphs generated in this study are provided in the Source Data file. A reporting summary for this article is available as an Additional Information file. Source data are provided with this paper.

## Source data

Source Data
