# Supplementary material for: Generation of hydroxyl radical-activatable ratiometric near-infrared bimodal probes for early monitoring of tumor response to therapy
Source: Nat Commun. 2021 Oct 22;12:6145. doi: 10.1038/s41467-021-26380-y (PMC8536768; doi:10.1038/s41467-021-26380-y)
Supplement: Supplementary file 1 — Supplementary Information [file 41467_2021_26380_MOESM1_ESM.pdf]

Supplementary Information

Generation of Hydroxyl Radical-activatable Ratiometric Near-infrared Bimodal Probes for Early Monitoring of Tumor Response to Therapy

Luyan Wu[1,#], Yusuke Ishigaki[2,#], Wenhui Zeng[1,#], Takashi Harimoto[2], Baoli Yin[3], Yinghan Chen[4], Shiyi Liao[3], Yongchun Liu[3], Yidan Sun[1], Xiaobo Zhang[1], Ying Liu[1], Yong Liang[4], Pengfei Sun[5], Takanori Suzuki[2,*], Guosheng Song[3,*], Quli Fan[5,*], and Deju Ye[1,*]

[1] State Key Laboratory of Analytical Chemistry for Life Science, Chemistry and Biomedicine Innovation Center (ChemBIC), School of Chemistry and Chemical Engineering, Nanjing University, Nanjing, 210023, China

[2] Department of Chemistry, Faculty of Science, Hokkaido University, N10 W8, North-ward, Sapporo 060-0810, Japan

[3] State Key Laboratory for Chemo/Bio-Sensing and Chemometrics, College of Chemistry and Chemical Engineering, Hunan University, Changsha, 410082, China

[4] Jiangsu Key Laboratory of Advanced Organic Materials, School of Chemistry and Chemical Engineering, Nanjing University, Nanjing, 210023, China

[5] State Key Laboratory of Organic Electronics and Information Displays & Institute of Advanced Materials (IAM), Nanjing University of Posts & Telecommunications, 9 Wenyuan Road, Nanjing 210023, China.

[#] These authors contributed equally to this work.

*e-mail: tak@sci.hokudai.ac.jp; songgs@hnu.edu.cn; iamqlfan@njupt.edu.cn; dejuye@nju.edu.cn

Table of Contents of Supplementary Information

		Page
1	Supplementary Materials and Methods	3-13
2	Supplementary Figs. 1-45	14-60
3	Supplementary Figs. 46-52: NMR, MS spectra and Crystal data	61-65
4	Supplementary Note 1	37
5	Supplementary Tables 1-4	66
6	Supplementary Dataset 1	69
7	References	69

1. Supplementary Materials and Methods

1.1 Materials

All chemicals were purchased from Sigma-Aldrich unless otherwise stated and used without further purification. 1,2-distearoyl-sn-glycero-3-phosphoethanolamine-N-[methoxy (polyethylene glycol)-2000] (DSPE-PEG$_{2000}$) and 1,2-distearoyl-sn-glycero-3-phosphoethanolamine-N-[folate (polyethylene glycol)-2000] (DSPE-PEG$_{2000}$-FA) were purchased from Avanti (Alabaster, AL, USA). Silicon 2,3-naphthalocyanine bis(trihexylsilyloxide) (NIR775), 1-Butyl-2-[2-[3-[(1-butyl-6-chlorobenz[cd]indol-2(1H)-ylidene)ethylidene]-2-chloro-1-cyclohexen-1-yl]ethenyl]-6-chlorobenz[cd]indolium tetrafluoroborate (IR1048), Lipopolysaccharides (LPS), Phorbol 12-myristate 13-acetate (PMA) and 4-Hydroxy-2,2,6,6-tetramethylpiperidine 1-oxyl (tempol) were purchased from Sigma-Aldrich. Erastin and Ferrostatin-1 (Fer-1) were obtained from MedchemExpress. 3-(4,5-dimethylthiazol-2-yl)-2,5-diphenyltetrazolium bromide (MTT) kit were obtained from KeyGen Biotech. Co. Ltd. (Nanjing, China).

1.2 Instrumentation

Transmission electron microscopic (TEM) images were acquired with a JEM-2800 transmission electron microscope (JEOL, Ltd., Japan) with an accelerating voltage of 200 kV. Column chromatography was performed on silica gel 60N (KANTO KAGAKU, spherical neutral) of particle size 40-50 μm or Wakogel® 60N (neutral) of particle size 38-100 μm, or aluminium oxide 90 standardized (Merck 63–200 μm). ^{1}H and ^{13}C NMR spectra were recorded on a BRUKER Ascend™ 400 (^{1}H/400 MHz and ^{13}C/100 MHz) spectrometer. IR spectra were measured as a KBr pellet on a JEOL JIR-WINSPEC100 FT/IR spectrophotometer. Mass spectra were recorded on a JMS-T100GCV spectrometer in FD mode (GC-MS & NMR Laboratory, Research Faculty of Agriculture, Hokkaido University). Melting points were measured on a Yamato MP-21 and are uncorrected. Elemental analyses were performed on an EXETER ANALYTICAL CE440 at the Center for Instrumental Analysis of Hokkaido University. UV/Vis/NIR spectra were recorded on a Hitachi U-2910 spectrophotometer. Redox

potentials (E^{ox} and E^{red}) were measured on a BAS ALS-600A by cyclic voltammetry in dry CH_2Cl_2 containing 0.1 M Bu_4NBF_4 as a supporting electrolyte. All of the values shown in the text are in E (V) vs. SCE measured at the scan rate of 100 mV s^{-1}. Pt electrodes were used as the working and counter electrodes. The working disk electrode was polished using a water suspension of aluminium oxide (0.05 μmol) before use. Crystal Data were collected with a Rigaku XtaLAB Synergy (Cu-Kα radiation, λ = 1.54184 Å). The structure was solved by ShelXT (Sheldrick) and refined by the full-matrix least-squares method on $F2$ with anisotropic temperature factors for non-hydrogen atoms. All the hydrogen atoms were located at the calculated positions and refined with riding. Olex2 was used for all calculations. The FL spectra of NIR775 were measured with a HORIBA Jobin Yvon Fluomax-4 fluorescence spectrometer. The FL spectra of IR1048 were carried out on HORIBA Fluolog3 spectrophotometer. Dynamic light scattering (DLS) and zeta potential analysis were measured by 90 Plus/BI-MAS equipment (Brookhaven, America). The UV-vis-NIR spectra were measured with UV-3600 UV-VIS-NIR Spectrophotometer (Shimadzu Corporation). FL images of NIR775 were acquired using an IVIS Lumina XR III system. FL images of IR1048 were conducted by using NIR-II imaging instruments (NIR-OPTICS Series III 900/1700 whole animal imaging system) (Suzhou NIR-OPTICS Co., Ltd., China) and home-built imaging set-up (CDD: NIRvana TE 640)). PA images were collected and analyzed on Endra Nexus128 PA tomography system and the multispectral optoacoustic tomography (MSOT) system (iThera Medical GmbH, Neuherberg, Germany). Radiation therapy was conducted on PXi X-RAD 225.

1.3 Methods

Synthesis of 1,1-bis(4-diethylaminophenyl)-2-(4-bromo-2,6-difluorophenyl)ethene (A-2)

To a solution of diethyl bis(4-diethylaminophenyl)methyl phosphonate [A-1] (1.48 g, 3.31 mmol) in dry THF (70 mL) was added ^{n}-BuLi (1.57 M in hexane, 2.14 mL, 3.36 mmol) dropwise over 10 min at –20 °C, and the mixture was stirred for 30 min. Then, 4-bromo-2,6-difluorobenzaldehyde (663 mg, 3.00 mmol) was added to the solution at –20 °C. After stirring at 25 °C for 3 h, the mixture was diluted with saturated NH_4Cl aq. The whole mixture was extracted with EtOAc for five times. The combined organic layers were washed with water and

brine, and dried over anhydrous Na_2SO_4. After filtration, the solvent was concentrated under reduced pressure. The residue was purified by column chromatography on silica gel (hexane/EtOAc = 40) to give A-2 (785 mg) as a yellow solid in 51% yield. m.p. 132-138 °C (decomp.); ^{1}H NMR (400 MHz, $CDCl_3$): δ = 7.24 (d, J = 9.2 Hz, 2H), 6.93 (d, J = 8.9 Hz, 2H), 6.92 (s, 1H), 6.90 (s, 1H), 6.61 (d, J = 9.0 Hz, 2H), 6.50 (d, J = 8.9 Hz, 2H), 6.24 (s, 1H), 3.42-3.29 (m, 8H), 1.21-1.12 (m, 12H) ppm; ^{13}C NMR (100 MHz, $CDCl_3$): δ = 160.39 (dd, J_{C-F} = 8.9, 250.9 Hz), 149.33, 147.74, 147.33, 130.91, 130.05, 129.79, 127.33, 118.44 (t, J_{C-F} = 12.2 Hz), 116.68 (t, J_{C-F} = 19.2 Hz), 114.91 (dd, J_{C-F} = 8.1, 21.0 Hz), 110.92, 110.86, 107.06, 44.37, 44.20, 12.68, 12.65 ppm; IR (KBr): ν = 3091, 3071, 3035, 2971, 2931, 2882, 1606, 1597, 1562, 1556, 1519, 1469, 1410, 1401, 1374, 1352, 1301, 1266, 1213, 1192, 1188, 1152, 1141, 1093, 1076, 1030, 1007, 943, 872, 858, 846, 819, 811, 781, 765, 738 cm^{-1}; LR-MS (FD): m/z (%): 516.15 (5), 515.15 (32), 514.14 (bp), 513.15 (33), 512.14 (M^+, 99); HR-MS (FD) calcd. for $C_{28}H_{31}BrF_2N_2O_2$: 512.16387; found: 512.16234.

Synthesis 1,1,4,4-tetrakis(4-diethylaminophenyl)-2,3-bis(4-bromo-2,6-difluorophenyl)-1,3-butadiene (1-Br-Et)

To a solution of A-2 (419 mg, 816 μmol) in dry CH_2Cl_2 (14 mL) was added tris(4-bromophenyl)aminium hexacholoroantimonate (667 mg, 816 μmol) at 0 °C, and the mixture was stirred for 1.5 h. The solvent was concentrated under reduced pressure and dried under vacuum to give a crude dicationic salt as a dark blue powder. The crude product was used in the next reaction without further purification. A solution of Bu_4NF trihydrate (1.93 g, 6.12 mmol) in MeCN (5 mL) was added to a solution of the dicationic salt in MeCN (6 mL) at –20 °C. After the solution was stirred at –20 °C for 30 min, Zn powder (5.34 g, 81.6 mmol) was added to the purple solution. The reaction mixture was stirred at –20 °C for an additional 1 h. After the addition of saturated $NaHCO_3$ aq., the whole mixture was extracted with ether for five times. The combined organic layers were dried over anhydrous Na_2SO_4. After filtration, the solvent was concentrated under reduced pressure. The crude product was purified by column chromatography on aluminium oxide (hexane/EtOAc = 40) to give 1-Br-Et (398 mg) as a yellow solid in 95% yield. m.p. 223-231 °C (decomp.); ^{1}H NMR (400 MHz, $CDCl_3$): δ = 7.69

(br, 2H), 6.82 (br-d, J = 6.9 Hz, 4H), 6.72 (br, 2H), 6.52 (br, 2H), 6.32 (br-d, J = 7.7 Hz, 4H), 6.41-5.98 (br, 6H), 3.40-3.15 (m, 16H), 1.17-1.02 (m, 24H) ppm; ^{13}C NMR (100 MHz, $CDCl_3$): δ = 148.52, 146.74, 146.68, 131.88, 131.37, 131.05, 125.59, 123.74, 122.11, 117.83, 114.50, 114.29, 111.11, 110.29, 44.46, 44.09, 12.72, 12.63 ppm; IR (KBr): ν = 3088, 3074, 3038, 2971, 2930, 2894, 1605, 1577, 1558, 1518, 1468, 1410, 1399, 1375, 1354, 1266, 1199, 1154, 1142, 1091, 1078, 1057, 1026, 1015, 959, 870, 835, 817, 785, 750 cm^{-1}; UV/Vis (CH_2Cl_2): λ_{max} (ε) 430 (34800), 361 (29600), 308 (34200), 240 nm (45900 mol^{-1}dm^{3}cm^{-1}); LR-MS (FD): m/z (%): 1028.31 (11), 1027.30 (34), 1026.30 (64), 1025.30 (61), 1024.30 (bp), 1023.30 (31), 1022.30 (M^+, 47) ; elemental analysis calcd (%) for $C_{56}H_{60}Br_2F_4N_4$: C 65.13, H 5.90, N 5.47; found: C 65.41, H 5.90, N 5.25.

Synthesis of (*Z*)-1,1,4,4-tetrakis(4-diethylaminophenyl)-2,3-bis(4-bromo-2,6-difluorophenyl)-2-butene-1,4-diylium bis(tetrafluoroborate) [2-Br-Et · (BF_4)$_2$]

To a solution of 1-Br-Et (155 mg, 150 μmol) in dry CH_2Cl_2 (5 mL) was added tris(4-bromophenyl)aminium tetrafluoroborate (172 mg, 300 μmol) at 0 °C, and the mixture was stirred at 25 °C for 1 h. The addition of dry ether led to precipitation of the dication salt. The precipitates were collected, washed with dry ether and dry hexane three times, and dried in vacuo to give 2-Br-Et (BF_4)$_2$ (131 mg) as a dark green powder in 72% yield. m.p. 198-200 °C (decomp.); ^{1}H NMR (400 MHz, CD_3CN): δ = 7.96 (br, 4H), 7.28 (br-d, J = 8.5 Hz, 8H), 6.80 (br-d, J = 9.1 Hz, 8H), 3.70-3.53 (m, 16H), 1.28-1.07 (m, 24H) ppm; ^{13}C NMR (100 MHz, acetone-d_6): δ = 168.58, 162.06, 159.58, 157.15, 155.17, 154.80, 147.76, 140.03, 130.54, 125.41, 124.73, 119.28, 116.71, 116.46, 116.04, 115.77, 114.54, 114.08, 45.91, 12.52, 12.13 ppm; IR (KBr): ν = 3082, 2973, 2928, 2872, 1610, 1578, 1415, 1388, 1343, 1275, 1189, 1157, 1125, 1084, 1071, 1030, 1004, 944, 916, 889, 864, 834, 787, 745, 707, 689 cm^{-1}; UV/Vis/NIR (CH_2Cl_2): λ_{max} (ε) 759 (50200), 555 (93300), 425 (23000), 372 (17700), 317 nm (28400 mol^{-1}dm^{3}cm^{-1}); LR-MS (FD): m/z (%): 1115.27 (5), 1114.27 (16), 1113.27 (33), 1112.27 (52), 1110.28 (26), 1109.28 (25), 1108.29 (6), 1028.32 (33), 1026.32 (63), 1025.32 (62), 1024.32 (bp), 1023.33 (32), 1022.32 ([M^{2+}+e^-]$^+$, 48), 513.64 (11), 513.14 (20), 512.64 (20), 512.14 (29), 511.64 (10), 511.14 (M^{2+}, 15); HR-MS (FD): m/z calcd for $C_{56}H_{52}Br_2F_4N_4O_4^{2+}$: 1022.31208;

found: 1022.31112.

Crystal data

Method

Data collection was conducted with a Rigaku Mercury 70 diffractometer (Mo-K radiation, = 0.71075 Å). The structure was solved by the direct method (SIR2004) and refined by the full-matrix least-squares method on F^2 with anisotropic temperature factors for non-hydrogen atoms. All the hydrogen atoms were located at the calculated positions and refined with riding.

Crystal data for 1-Br-Et: CCDC2063588

Crystals were obtained by recrystallization from $CHCl_3$/hexane. MF: $C_{56}H_{60}N_4F_4Br_2$ FW: 1024.90, yellow plate, 0.70 × 0.50 × 0.10 mm^3, monoclinic $P2_1/n$, a = 16.4166(3) Å, b = 20.2146(5) Å, c = 18.6730(4) Å, β = 105.388(2) °, V = 5974.6(2) $Å^3$, ρ (Z = 4) = 1.139 g cm^{-3}. A total 41018 reflections were measured at T = 150 K. Numerical absorption correction was applied (μ = 2.099 mm^{-1}). The final $R1$ and $wR2$ values are 0.1073 (I > 2σI) and 0.3161 (all data) for 12036 reflections and 603 parameters. Estimated standard deviations are 0.004-0.011 Å for bond lengths and 0.3-0.9° for bond angles.

Crystal data for 2-Br-Et $(BF_4)_2$: CCDC2063589

Crystals were obtained by recrystallization from $CHCl_3$/hexane. MF: $C_{56}H_{60}B_2N_4F_{12}Br_2$ FW: 1198.52, violet needle, 0.70 × 0.10 × 0.05 mm^3, monoclinic $P2_1/c$, a = 27.8469(4) Å, b = 10.61795(16) Å, c = 61.2719(10) Å, β = 96.7143(17) °, V = 17992.4(5) $Å^3$, ρ (Z = 12) = 1.327 g cm^{-3}. A total 127948 reflections were measured at T = 150 K. Numerical absorption correction was applied (μ = 2.363 mm^{-1}). The final $R1$ and $wR2$ values are 0.1157 (I > 2σI) and 0.3466 (all data) for 36284 reflections and 2084 parameters. Estimated standard deviations are 0.006-0.07 Å for bond lengths and 0.4-3.0° for bond angles.

EMs 1-F-Me and 1-Br-Me were synthesized and characterized according to previously reported methods [1].

Reduction of 2-Br-Et $(BF_4)_2$ to 1-Br-Et. A mixture of dication salt 2-Br-Et $(BF_4)_2$ (102 mg, 85.1 μmol) and Zn powder (559 mg, 8.55 mmol) in dry MeCN (4 mL) was stirred at 25 °C for

30 min. The resulting suspension was diluted with water. The whole mixture was extracted with EtOAc five times. The combined organic layers were washed with water and brine, and dried over anhydrous Na_2SO_4. After filtration through aluminium oxide, the solvent was concentrated under reduced pressure to give 1-Br-Et (86.3 mg) as a yellow solid in 99% yield.

Investigation of the reaction between 1-Br-Et and •OH. Briefly, 1-Br-Et (28 μM) in 1.0 mL D.I. water (10% DMF) was incubated with •OH (100 μM Fe^{2+} + 1 mM H_2O_2) at r.t. for 3 min. The reaction was then analyzed by HPLC, and the UV-vis absorption spectra before and after incubation with •OH were acquired.

Measurement of the sensitivity of 1-NP for •OH. To test the sensitivity of 1-NP for •OH based on the ratiometric FL imaging, 1-NP (56/1.65/20 μM 1-Br-Et/NIR775/IR1048) was incubated with varying concentrations of •OH (0, 2, 5, 10, 20, 30, 50, 100, 150, 200, 250 μM Fe^{2+} + 1 mM H_2O_2) in D.I. water at r.t. for 3 min. Then, the UV-vis-NIR absorption spectra were acquired. The FL spectra of NIR775 in the incubation solutions were recorded, with excitation from 700 to 900 nm and emission from 710 to 910 nm, by a synchronous scanning mode on a HORIBA Jobin Yvon Fluoromax-4 fluorometer, with an offset of 10 nm. The FL spectra of IR1048 in the incubation solutions were recorded with excitation at 808 nm and and emission with bandpass between 900-1500 nm on a HORIBA Fluolog3 spectrophotometer. The FL intensities at 780 and 1113 nm were recorded, and the FL_{780}/FL_{1113} value was computed by dividing the FL intensity at 780 nm to that at 1113 nm of each solution. The FL intensities at 780 nm and 1113 nm, and the FL_{780}/FL_{1113} values were all normalized to the values of 1-NP incubated with 0 μM •OH. Plot of the normalized FL_{780}/FL_{1113} ratios versus the concentrations of •OH yields a linear regression between 0.05–20 μM •OH. The slope (k) of the linear plot was obtained, and the standard deviations (δ) was obtained from 11 times' measurements of a blank solution. The LOD of based on the ratiometric FL detection was calculated by using the $3\delta/k$ method.

To test the sensitivity of 1-NP for •OH based on the ratiometric PA imaging, the resulting PA images of above-mentioned solutions were acquired at both 755 and 905 nm on the Endra Nexus128 PA tomography system. The corresponding PA intensities were recorded, and the PA_{755}/PA_{905} ratios were computed by dividing the PA intensity at 755 nm to that at 905 nm. The

PA intensities at 755 nm and 905 nm, and the PA_{755}/PA_{905} values were all normalized to the values of 1-NP incubated with 250 μM •OH. Plot of normalized PA_{755}/PA_{905} ratio versus the concentration of •OH afford a linear regression between 1-20 μM •OH. The slope (*k*) of the linear plot was obtained, and the standard deviations (δ) was obtained from 11 times' measurements of a blank solution. The LOD of based on the ratiometric PA detection was calculated by using the 3δ/*k* method.

Investigation of the selectivity of 1-NP toward •OH. To examine the specificity of 1-NP toward •OH, 1-NP (56/1.65/20 μM 1-Br-Et/NIR775/IR1048) in PBS buffer were treated with different ROS (•OH (200 μM Fe^{2+} + 1 mM H_2O_2), $^1O_2$ (1 mM H_2O_2 + 1 mM ClO^-), 300 μM t-BuOOH, 1 mM ClO^-, 300 μM CuOOH, 1 mM H_2O_2, $O_2^{\bullet-}$ (100 μM xanthine + 22 mU xanthine oxidase (XO)), $ONOO^-$ (1 mM $NaNO_2$ + 1 mM H_2O_2)) at r.t. for 24 h. The FL spectra of NIR775 were recorded with excitation from 700 to 900 nm and emission of 710 to 910 nm by a FL synchronous scanning mode on the HORIBA Jobin Yvon Fluoromax-4 fluorometer, with an offset of 10 nm. The FL spectra of IR1048 were recorded with excitation at 808 nm on the HORIBA Fluolog3 spectrophotometer. FL images were measured with an IVIS Lumina XR III system (NIR775: λ_{ex}=740 nm) and a NIR-OPTICS Series III 900/1700 whole animal imaging system (IR1048: λ_{ex}=808 nm), respectively. The corresponding FL_{780}/FL_{1113} values were calculated, and then normalized to that of 1-NP alone (control). PA images and intensities at 755 and 905 nm were collected and analyzed on the Endra Nexus128 PA tomography system. The corresponding PA_{755}/PA_{905} values were calculated, and then normalized to that of 1-NP incubated with •OH.

Computational methods. The DFT calculations were performed with Gaussian 09. Geometry optimizations of the ground-state and transition-state structures were carried out at the (U)M06-2X level of theory with the 6-31G(d) basis set in water using the SMD solvation model. Vibrational frequencies were computed at the same level to verify that optimized structures are local minimums or transition states. Then the energies of the optimized structures in water were computed at the more accurate (U) M06-2X/6-311+G(d,p) level with the SMD model.

Measurement of the reaction kinetics between diene EM and •OH, or 1-NP and •OH. To

measure the reaction kinetics between 1-F-Me and •OH, 1-F-Me (8 μM in D.I. water (containing 50% DMF) in a 96-well transparent plate was incubated with varying concentrations of •OH (0, 100, 150, 200, 250 μM Fe^{2+} + 1 mM H_2O_2) at r.t.. To measure the reaction kinetics between 1-Br-Me, or 1-Br-Et and •OH, 1-Br-Me or 1-Br-Et (8 μM) in D.I. water (containing 50% DMF) in a 96-well transparent plate was incubated with varying concentrations of •OH (0, 50, 80,100, 200 μM Fe^{2+} + 1 mM H_2O_2) at r.t.. To measure the reaction kinetics between 1-NP and •OH, 1-NP (8/0.2/2.9 μM 1-Br-Et/NIR775/IR1048 in D.I. water) in a 96-well transparent plate was incubated with varying concentrations of •OH (0, 50, 80, 100 and 200 μM Fe^{2+} + 1 mM H_2O_2) at r.t.. The UV-vis absorbance of each reaction solution at 560 nm was monitored every 20 s on the T-can microplate reader. The UV-vis absorbance at each time point was normalized to the maximum absorbance achieved. The pseudo-first-order rate (k_{obs}) of 1-F-Me, 1-Br-Me, 1-Br-Et or 1-NP incubated with indicated concentration of •OH was determined by fitting the normalized absorption intensity with single exponential function of:

$y = y_0 + A \times \exp(R_0 \times t)$, where $k_{obs} = -R_0$.

Plot of k_{obs} versus •OH concentration affords the second-order reaction rate, k_2.

Cytotoxicity assay (MTT). The RAW264.7 macrophages or 4T1 cells were seeded on 96-well plates (1× 10^4 cells/well) for 24 h. 1-NP or 1-NP-FA (0, 1, 2, 5, 10, 15, 20, 30, 40 and 50 μM based on the concentration of IR1048) in DMEM medium were then added. The cells were incubated for another 24 h, followed by the addition of MTT (50 μL, 1 mg mL^{-1}) and incubation for 4 h at 37 °C. Subsequently, the solution in each well was carefully removed, and 150 μL DMSO was added into each well to dissolve the purple formazan crystals. The absorbance (OD) at 550 nm in each well was acquired on the T-can microplate reader.

Imaging of exogenous •OH in vivo. To investigate the ability of 1-NP for ratiometric FL/PA bimodality imaging of Fenton reagent-induced production of •OH in vivo, PBS buffer containing 1-NP (112/3.3/40 μM 1-Br-Et/NIR775/IR1048, 50 μL) or 1-NP plus 200 μM Fe^{2+} and 1 mM H_2O_2 was subcutaneously injected into the tight of mice. After 10 min, FL images of NIR775 and IR1048 in mice were measured and analyzed with the IVIS Lumina XR III

system and NIR-OPTICS Series III 900/1700 whole animal imaging system (NIR775: λ_{ex} =740 nm; IR1048: λ_{ex}=808 nm), respectively. FL intensities were quantified using region of the interest (ROI) over the images in the Living Image Software for NIR775 and the ImageJ software (NIH) for IR1048. PA images and intensities at 755 and 905 nm were collected and analyzed on the multispectral optoacoustic tomography (MSOT) system. The corresponding FL_{780}/FL_{1113} and PA_{755}/PA_{905} values were calculated, and applied for the generation of ratiometric images.

The ratio calculation method. To calculate the FL ratio between 780 and 1113 nm in tumor, the tumor FL intensity at 780 nm in each image was quantified by drawing region of the interest (ROI) over tumor location using the Living Image Software, and the tumor FL intensity at 1113 nm in each image was quantified by drawing ROI over tumor location using the ImageJ software (NIH). By dividing the tumor FL intensity at 780 nm to that at 1113 nm, the FL ratio between 780 nm and 1113 nm in each tumor following different treatment could be calculated, which was then normalized to that of saline-treated control tumors. To obtain the ratiometric FL images of tumors in vivo, FL images of the same tumor at 780 and 1113 nm were adjusted to the same size, and then opened in the ImageJ simultaneously; the type of images was adjusted to 8 bit. By using the Calculator Plus Plugin provided by the ImageJ software, the corresponding ratiometric FL_{780}/FL_{1113} images of the tumor could be created.

To calculate the PA ratio between 755 and 905 nm, PA images of tumors were reconstructed by viewMSOT, the scanner equipped software for data post-processing. Since the PA signal of 1-NP-FA at 905 was 'always on', which could produce strong PA signals at 905 nm to locate the tumor region, ROI was draw over the tumor in the PA image at 905 nm to quantify the PA intensity in the reconstructed PA images using the viewMSOT software, according to the recommended protocol by MSOT inVision. The ROI in the reconstructed PA image at 905 nm was automatically draw over the tumor location in the reconstructed PA image at 755 nm in the viewMSOT software to give the tumor PA intensity at 755 nm. By dividing the tumor PA intensity at 755 nm to that at 905 nm, the PA ratio between 755 nm and 905 nm in each tumor following different treatment could be calculated, which was then normalized to that of saline-

treated control tumors. Ratiometric PA images of tumors could be created by using the Calculator Plus Plugin provided by the ImageJ software.

Western blot (WB) analysis of GPX4 expression. To conduct the WB analysis of GPX4 expression level in 4T1 cells, cells were incubated with or without erastin for 8 h. Then the cells were washed with PBS. Scrape the cells from the culture dish and collect in cell lysis buffer. After that, in the presence of the cell lysate buffer, the cells were lysed on ice for 30 min and centrifuged at 12000 ×g for 5 min to obtain cell lysates. The protein was separated by SDS-PAGE (×1) and transferred to the nitrocellulose membrane by electrophoretic blotting. The membrane was sealed in 5% skimmed milk powder (dispersed in TBST buffer with 0.1% Tween 20) at room temperature for 1 h, incubated with primary antibody against GPX4 (HuaBio, Catalog # ET1706-45, dilution 1:1000) for 30 min at room temperature and stored at 4 ºC overnight. The next day, the membrane was incubated with the primary antibody at room temperature for 30 min. After washing with TBST for four times, the membrane was incubated with horseradish peroxidase (HRP)-conjugated secondary antibody goat anti-rabbit immunoglobulin G (IgG) at room temperature for 1 h. Then, the membrane was washed with TBST 4 times. The secondary antibody on the membrane was visualized with ECL detection reagent using G:BOX chemiXR5. Image J was used to quantify the band intensity. The experiments were repeated for three times.

FL imaging •OH in tumor tissue slices. The •OH levels in the tumor tissue slices resected from mice were tested using the commercial •OH indicator, 3'-p-(hydroxyphenyl) fluorescein (HPF). 4T1 tumor-bearing mice were i.p. injected with saline (0.9%), erastin (20 mg/kg) or erastin (20 mg/kg) together with 20 mg/kg Fer-1. After 12 h later, mice were intratumoral (i.t.) injected with HPF (20 μM), and after another 30 min, mice were sacrificed and tumors were resected. The isolated tumors tissues were cut using a vibrating-blade microtome to obtain 4 μm-thickness slices. After staining with DAPI, the images of tumor tissue slices were acquired with the IX73 fluorescent inverted microscope equipped with DAPI and FITC filters.

FL and PA imaging of organs from mice. Mice with s.c. 4T1 tumors were i.p. injected with saline, erastin (20 mg/kg) or erastin (20 mg/kg) plus Fer-1 (20 mg/kg), and 12 h later, 1-NP-FA

(1.68/0.05/0.6 mM 1-Br-Et/NIR775/IR1048, 200 μL) were i.v. injected into mice. After 24 h, the mice were perfused at elevated perfusion pressure with physiological saline immediately before main organs including heart, liver, spleen, lung, kidneys, tumor excision to remove blood from circulation. Mice were subsequently sacrificed and main organs (heart, liver, spleen, lung, kidneys, tumor) were resected. The isolated organs were washed 3 times with PBS (1×, pH 7.4). FL and PA images of organs were acquired. Ratiometric images of organs were obtained by dividing region of the interest (ROI) in FL or PA images of organs at 780 nm or 755 nm with its corresponding ROI in FL or PA images of organs at 1113 nm or 905 nm using Calculator Plus in the ImageJ software (NIH).

2. Supplementary Figs. 1-45

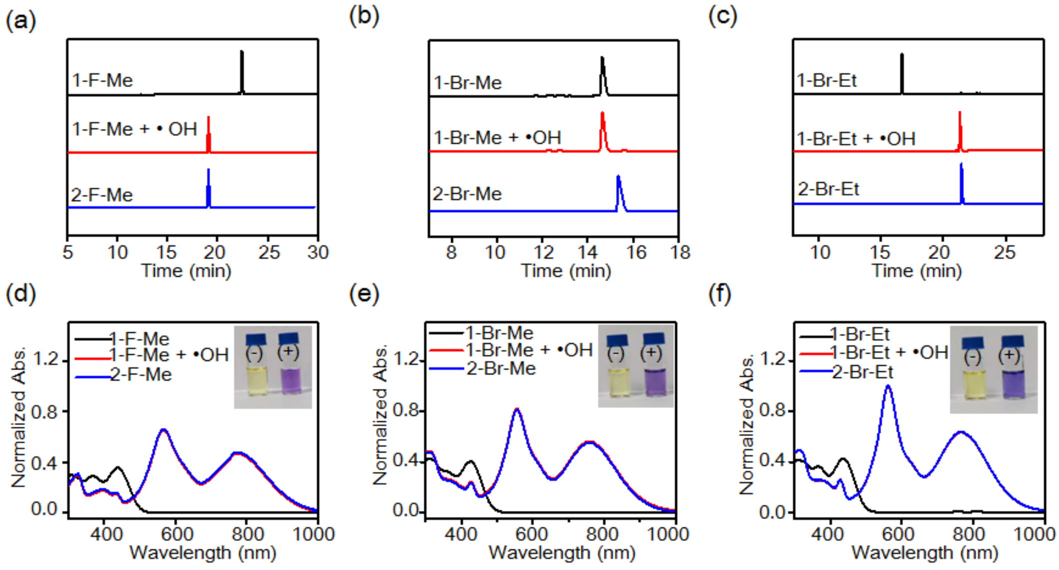

Supplementary Fig. 1 The reaction of diene electrochromic material toward •OH in vitro. (a) High performance liquid chromatography (HPLC) traces and (d) normalized UV-vis-NIR absorption spectra of 1-F-Me (black), 1-F-Me after reaction with •OH (red), and the synthetic 2-F-Me (blue). (b) HPLC traces and (e) normalized UV-vis-NIR absorption spectra of 1-Br-Me (black), 1-Br-Me after reaction with •OH (red), and the synthetic 2-Br-Me (blue). (c) HPLC traces and (f) normalized UV-vis-NIR absorption spectra of 1-Br-Et (black), 1-Br-Et after reaction with •OH (red), and the synthetic 2-Br-Et (blue).

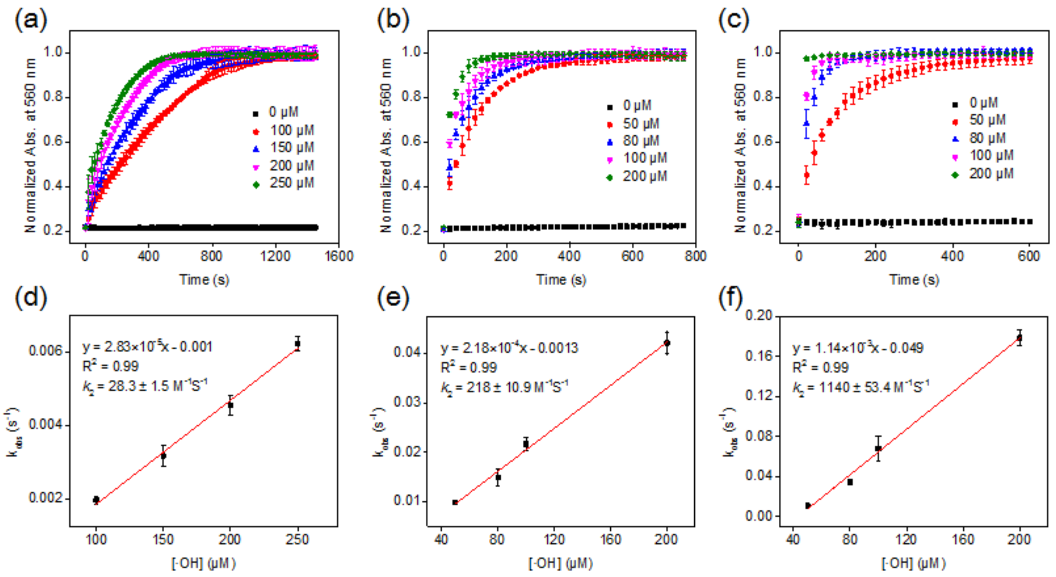

Supplementary Fig. 2 Kinetics studies of diene electrochromic material with •OH. Normalized time-dependent changes of the absorbance at 560 nm of (a) 1-F-Me, (b) 1-Br-Me and (c) 1-Br-Et (8 μM) upon incubation with •OH in D.I. water (containing 50% DMF). Plots of the pseudo-first-order rate (k_{obs}) versus •OH concentration afford the second-order reaction rate k_2 between 1-F-Me (d), 1-Br-Me (e) and 1-Br-Et (f) and •OH, respectively. The k_{obs} was determined by fitting the absorption intensity with single exponential function of $y = y_0 + A \times \exp(R_0 \times t)$, where $k_{obs} = -R_0$, and the k_2 value was obtained from the slope of the linear plot between k_{obs} and •OH concentration. Data are presented as mean ± s.d. (n = 3 independent samples). These findings revealed that 1-Br-Et hold the fast reaction kinetics ($k_2 =$ 1140 ± 53.4 M^{-1} s^{-1}) among the three synthesized dienes. Source data are provided as a Source Data file.

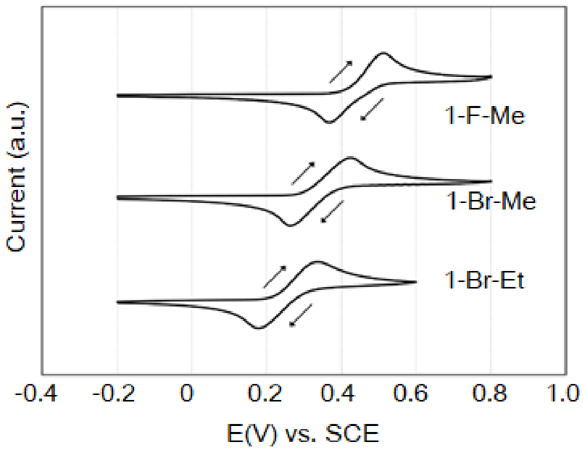

Supplementary Fig. 3 Cyclic voltammograms (CV) of 1-F-Me, 1-Br-Me and 1-Br-Et. The CV was measured in CH_2Cl_2 (1.0 mM) containing 0.1 M $Bu_4N^+BF_4^-$ as a supporting electrolyte (*E*/V vs. SCE, scan rate 100 mVs^{-1}, Pt electrode). The redox potentials (E^{ox}) of 1-F-Me, 1-Br-Me and 1-Br-Et was found to be +0.48 V, +0.40 V and +0.31 V, respectively. The lower E^{ox} of 1-Br-Et could make it easier oxidation by •OH.

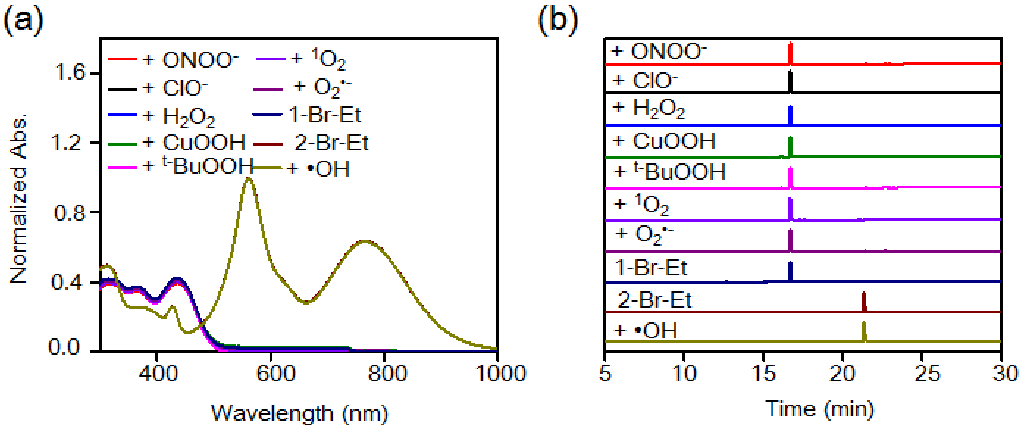

Supplementary Fig. 4 The specificity of 1-Br-Et toward •OH over other reactive oxygen species (ROS) in vitro. (a) UV-Vis-NIR absorption spectra and (b) high performance liquid chromatography (HPLC) traces of 2-Br-Et, 1-Br-Et or 1-Br-Et (28 μM) following incubation with •OH (100 μM Fe^{2+} + 1 mM H_2O_2) and other representative ROS, including $^1O_2$ (1 mM H_2O_2 + 1 mM ClO^-), 300 μM ^{t}BuOOH, 1 mM ClO^-, 300 μM CuOOH, 1 mM H_2O_2, $O_2^{\bullet-}$ (100 μM xanthine + 22 mU xanthine oxidase (XO)), $ONOO^-$ (1 mM $NaNO_2$ + 1 mM H_2O_2)) in D.I. water (containing 10% DMF) at r.t. for 3 min. These results show that only •OH could react with 1-Br-Et, yielding 2-Br-Et with distinct red-shift of UV-vis absorption from 436 nm to 560 and 767 nm.

(a)

ROS	·OH	HClO	H_2O_2	$^1O_2$	$O_2^{\bullet-}$
Reaction energy (kcal/mol)	-36.0	-16.7	-0.10	+9.0	+85.1

(b)

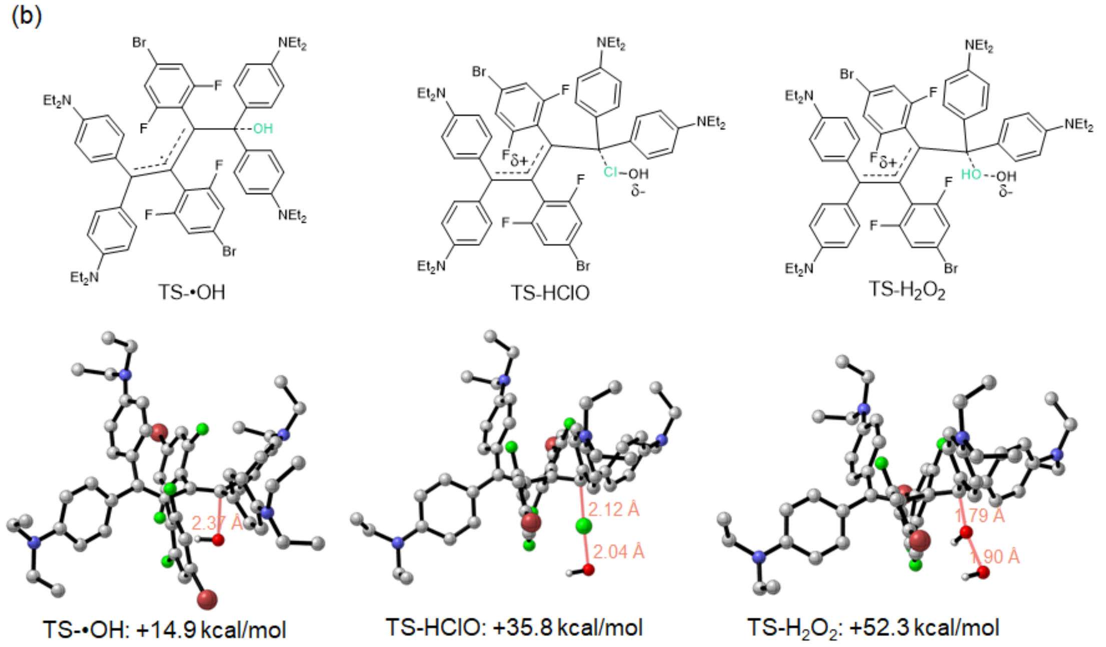

Supplementary Fig. 5 Density functional theory (DFT) calculations of the reactions between 1-Br-Et and representative reactive oxygen species (ROS). (a) The reaction energy of the redox reactions between 1-Br-Et and •OH, HClO, H_2O_2, $^1O_2$ or $O_2^{\bullet-}$. (b) DFT-computed transition-state structures and activation free energies of TS-•OH, TS-HClO and TS-H_2O_2. The coordinates and energies of stationary points regarding the DFT calculations were summarized in Supplementary Dataset 1. The overall reaction energies of 1-Br-Et and •OH or 1-Br-Et and HClO are negative, indicating an exergonic reaction, which is thermodynamically favorable. However, the reaction energy of 1-Br-Et and $^1O_2$ or 1-Br-Et and $O_2^{\bullet-}$ are positive, indicating that these reactions are endergonic. It was notable that the reaction energy (ΔG) was estimated to be about -36.0 kcal/mol between 1-Br-Et and •OH, much more favorable than that of HClO (ΔG = -16.7 kcal/mol) and H_2O_2 (ΔG = -0.10 kcal/mol). We further conducted the DFT calculations to compare the activation barrier between the reaction of 1-Br-Et with •OH, HClO and H_2O_2, respectively. We optimized the transition-state structures of 1-Br-Et with •OH, HClO and H_2O_2 (TS-•OH, TS-HClO and TS-H_2O_2) accordingly. The DFT-computed activation free energy for TS-•OH was estimated to be only +14.9 kcal/mol, suggesting that the reaction could rapidly

proceed between 1-Br-Et and •OH at ambient temperature. Whereas the energy barrier of TS-HClO or TS-H_2O_2 was estimated to be +35.8 kcal/mol and +52.3 kcal/mol, respectively, which was too high for the reactions under ambient conditions. All these computational results are consistent with the reactivity observed in our experiments. The DFT calculations support the specific and fast oxidation of 1-Br-Et by •OH rather than other ROS.

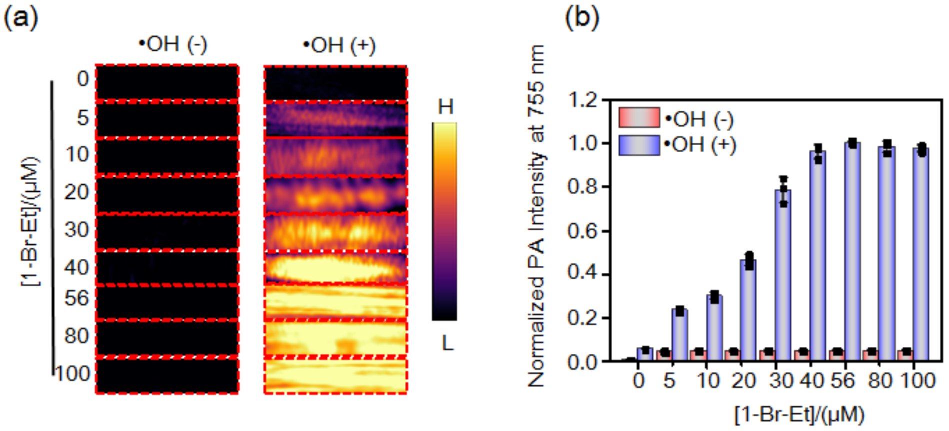

Supplementary Fig. 6 Investigation of the photoacoustic (PA) properties of 1-Br-Et before and after reaction with •OH. (a) PA images and (b) normalized PA intensities of different concentration of 1-Br-Et at 755 nm before and after incubation with •OH (350 μM Fe^{2+} + 1 mM H_2O_2) in D.I. water (10% DMF) at r.t. for 3 min. Data are presented as mean ± s.d. (n = 3 independent samples). These results demonstrate that 1-Br-Et itself showed weak PA signals, but after oxidation by •OH, probe's concentration dependent enhancement of PA signals occurred. The PA signals reach a plateau when 56 μM 1-Br-Et was converted into 2-Br-Et by •OH, with a significant ~22-fold increment in PA signal observed. Source data are provided as a Source Data file.

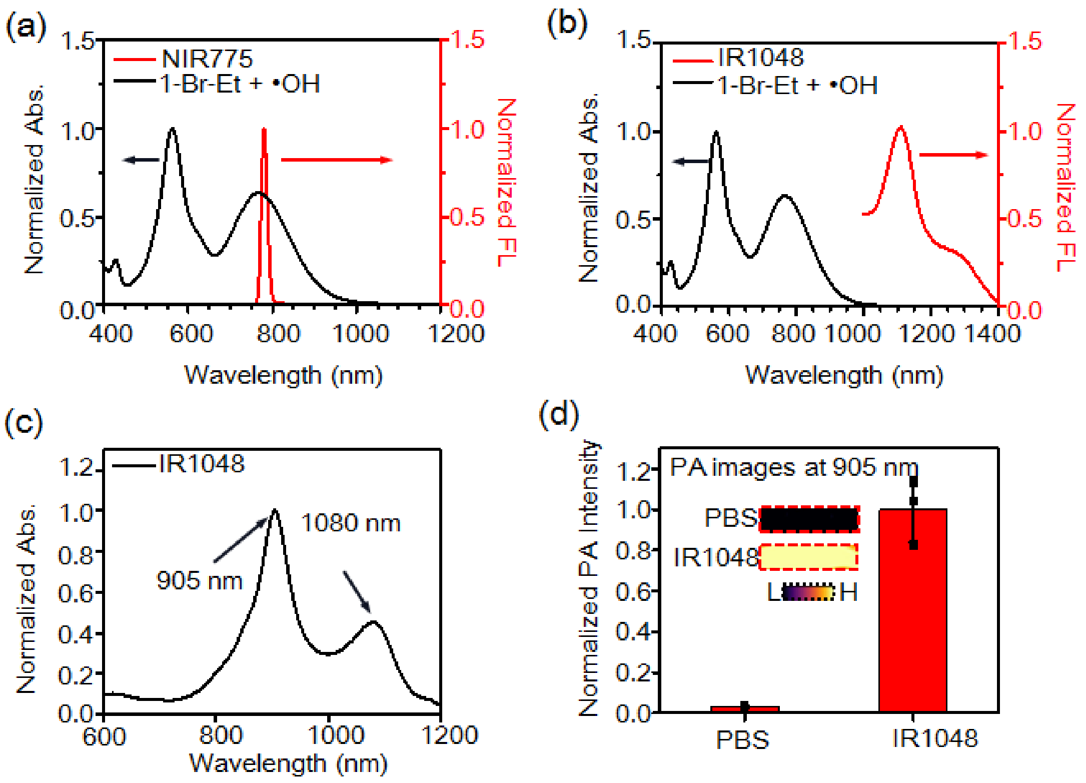

Supplementary Fig. 7 Comparison of the optical properties of NIR775, IR1048 and 1-Br-Et after reaction with •OH. (a) Normalized UV-Vis-NIR absorption spectra of 1-Br-Et after reaction with •OH (2-Br-Et) and fluorescence spectra of NIR775 show a good spectral overlap between them. (b) UV-Vis-NIR absorption spectra of 1-Br-Et after reaction with •OH (2-Br-Et) and fluorescence spectra of IR1048 show little spectral overlap between them. (c) Normalized absorption spectra of IR1048 show strong absorption at 905 and 1080 nm. (d) Photoacoustic (PA) intensities and images (inset) of a PBS buffer and a PBS buffer containing IR1048 (20 µM) at 905 nm show a strong PA signal of IR1048 at 905 nm. Data are presented as mean ± s.d. (n = 3 independent samples). To allow soluble in PBS buffer for the measurement, IR1048 was encapsulated in micellar NPs using a DSPE-PEG$_{2000}$-assisted nanoprecipitation approach. Source data are provided as a Source Data file.

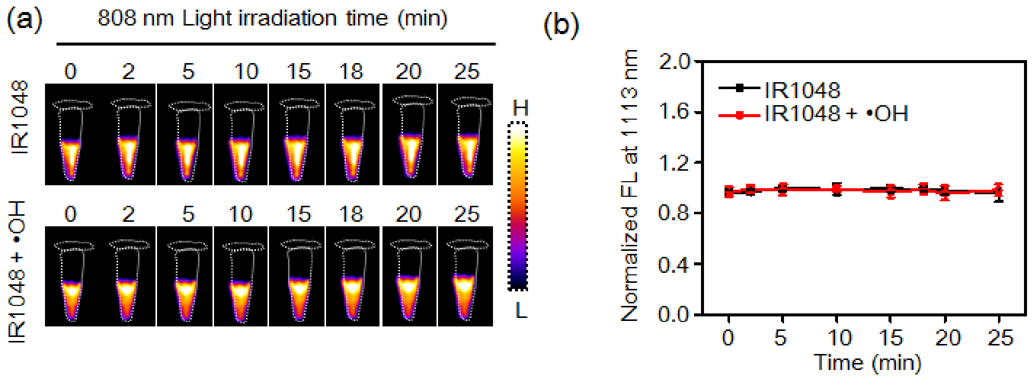

Supplementary Fig. 8 Investigation of the photostability of IR1048 and IR1048 in the presence of •OH. (a) NIR-II fluorescence (FL) images and (b) normalized FL intensities of IR1048 (20 μM) and IR1048 in the presence of •OH following continuous excitation at 808 nm (0.4 W cm^{-2}) for 25 min. Data are presented as mean ± s.d. (n = 3 independent samples). IR1048 was encapsulated in DSPE-PEG_{2000}-based micellar NPs to make it soluble in a PBS buffer for the measurement. The results show that IR1048 was inert to •OH and highly resistant to photobleaching by 808 light irradiation. Source data are provided as a Source Data file.

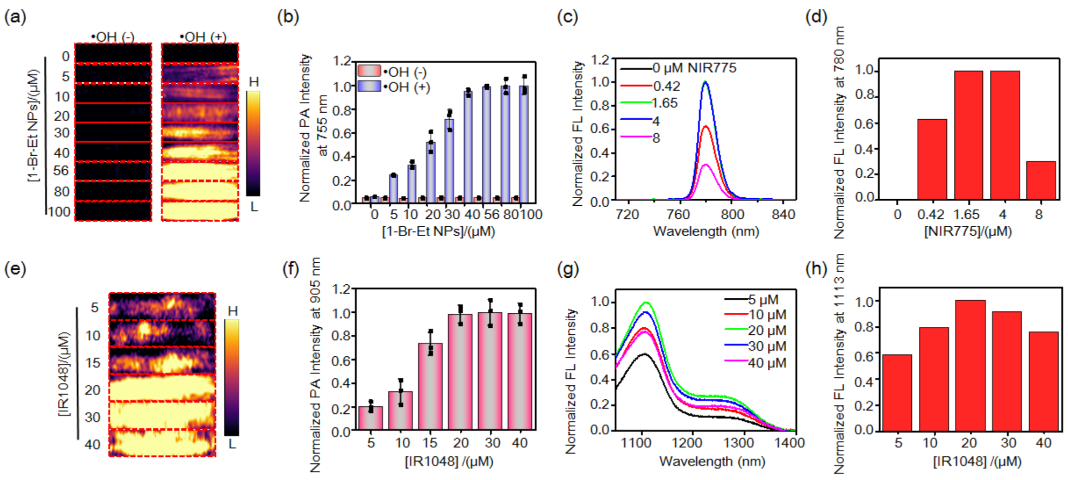

Supplementary Fig. 9 Optimization of the ratio of 1-Br-Et, NIR775 and IR1048 to prepare 1-NP. (a) Photoacoustic (PA) images and (b) PA intensities (at 755 nm) of different concentration of 1-Br-Et upon encapsulation in DSPE-PEG$_{2000}$-based micellar NPs after reaction without or with •OH (350 μM Fe^{2+} + 1 mM H_2O_2) in aqueous solution at r.t. for 3 min. (c) Normalized fluorescence (FL) spectra and (d) intensities at 780 nm of different concentration of NIR775 upon encapsulation in DSPE-PEG$_{2000}$-based micellar NPs. (e) PA images and (f) intensities (at 905 nm) of different concentration of IR1048 after being encapsulation in DSPE-PEG$_{2000}$-based micellar NPs. (g) Normalized FL spectra and (h) intensities at 1113 nm of different concentration of IR1048 upon encapsulation in DSPE-PEG$_{2000}$-based micellar NPs. Data are presented as mean ± s.d. (n = 3 independent samples). PA intensity at 755 nm reached the maximum when 56 μM 1-Br-Et (0.52 mg) was encapsulated with 10 mg DSPE-PEG in 9 mL D.I. water. The further increase of the amount of encapsulated 1-Br-Et to 80 and 100 μM did not increase the PA intensity, suggesting that 56 μM (0.52 mg) of 1-Br-Et might be the optimum amount to be encapsulated. Similarly, FL spectra of NIR775 NPs showed that the FL intensity of NIR775 at 780 nm reached the plateau when 1.65 μM NIR775 (0.02 mg) was encapsulated. NIR-II FL spectra showed that both the FL and PA intensities of IR1048 NPs could reach a plateau when 20 μM IR1048 (0.13 mg) was encapsulated. In all, these results suggest that the optimum mass of 1-Br-Et, NIR775 and IR1048 to be encapsulated by 10 mg DSPE-PEG was ~0.52 : 0.02 : 0.13 mg. Source data are provided as a Source Data file.

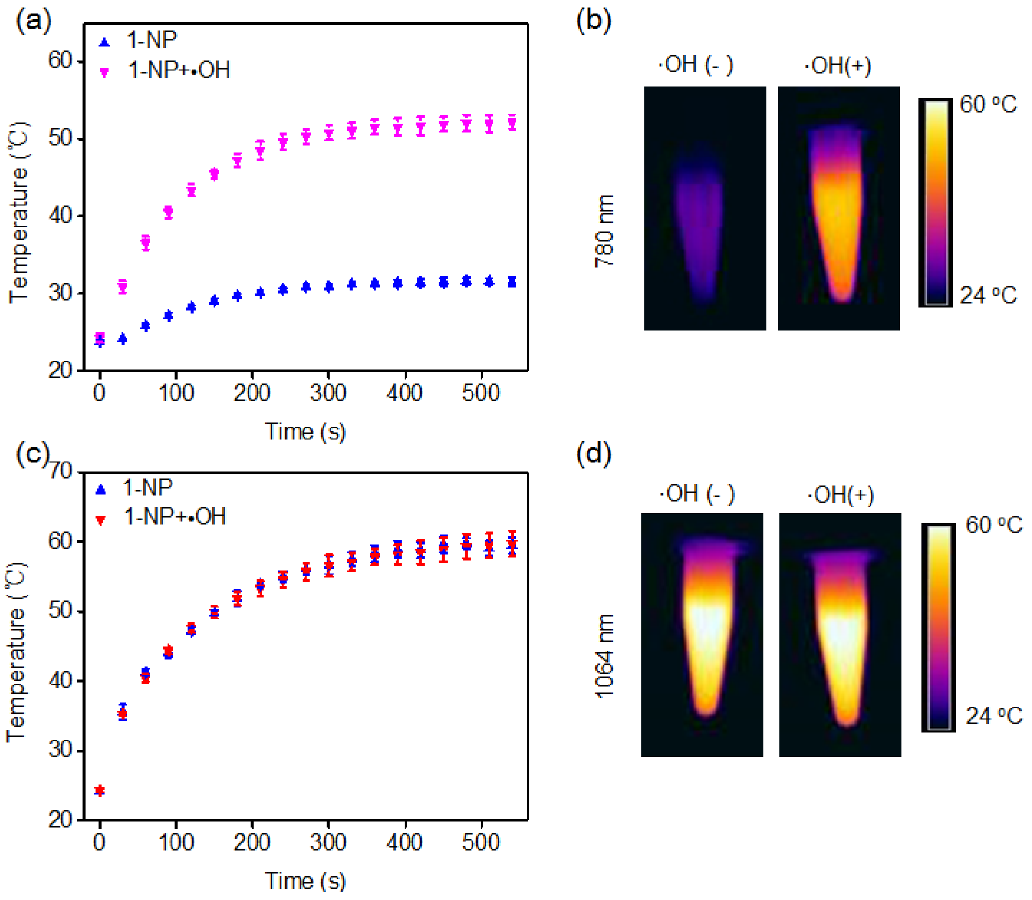

Supplementary Fig. 10 Investigation of the photothermal effect of 1-NP and 2-NP (1-NP + •OH). (a) Photothermal curves and (b) photothermal images of 1-NP and 2-NP under irradiation with a 780 nm laser (0.5 W cm^{-2}). (c) Photothermal curves and (d) photothermal images of 1-NP and 2-NP under irradiation with a 1064 nm laser (1.0 W cm^{-2}). Photothermal images were acquired at 5 min. Data are presented as mean ± s.d. (n = 3 independent samples). The photothermal images showed that the photothermal effect of 1-NP and 2-NP was similar at 1064 nm, while 2-NP exhibited much higher photothermal effect at 780 nm compared to 1-NP, according to the enhanced absorption and photoacoustic (PA) signal at ~767 nm, not at 905 nm. Source data are provided as a Source Data file.

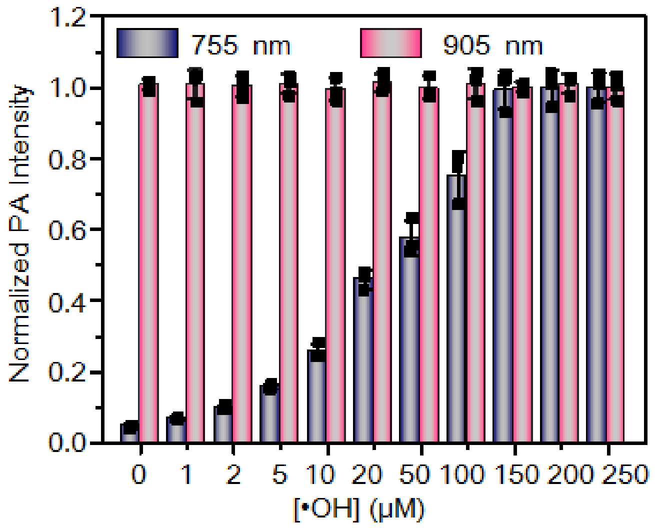

Supplementary Fig. 11 Normalized photoacoustic (PA) intensities of 1-NP (56/1.65/20 μM 1-Br-Et/NIR775/IR1048) at 755 and 905 nm after incubation with different concentration •OH in aqueous solution at r.t. for 3 min. Data are presented as mean ± s.d. (n = 3 independent samples). Source data are provided as a Source Data file.

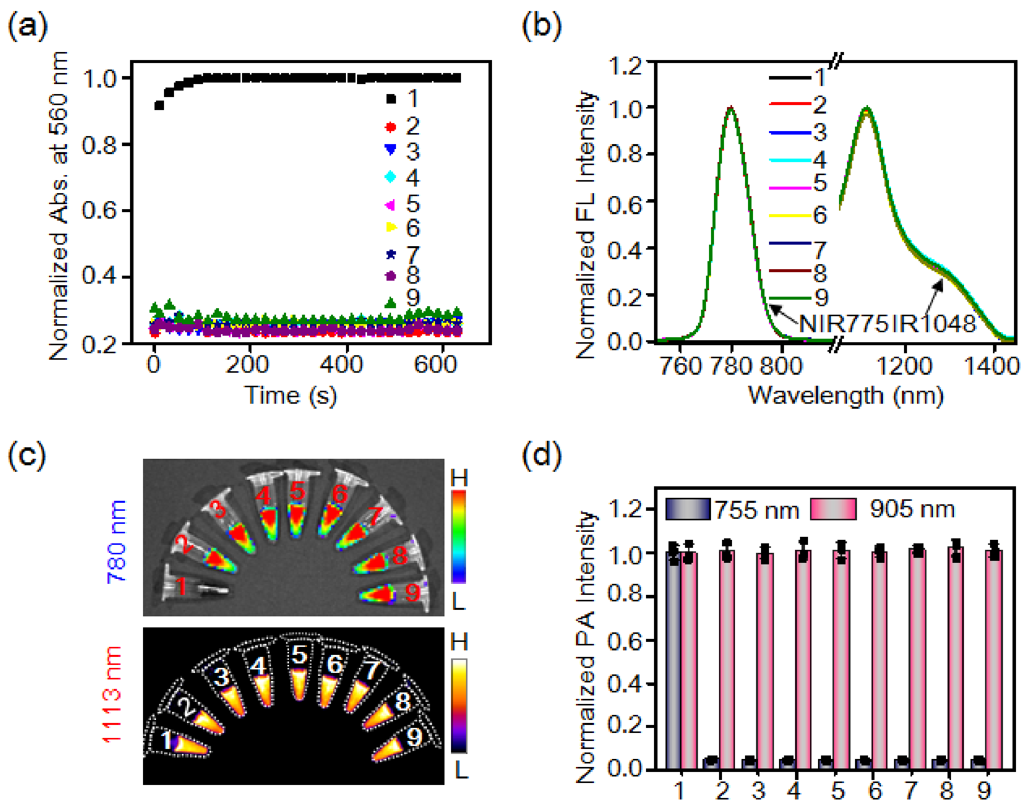

Supplementary Fig. 12 Investigation of the selectivity of the 1-NP toward •OH in vitro. (a) Time-dependent change of the absorbance of 1-NP at 560 nm upon incubation with •OH and other representative reactive oxygen species (ROS) at r.t. for over 600 s. (b) Fluorescence (FL) spectra and (c) images of 1-NP (56/1.65/20 μM 1-Br-Et/NIR775/IR1048) following incubation with •OH and other representative ROS. (d) Normalized photoacoustic (PA) intensities of 1-NP (56/1.65/20 μM 1-Br-Et/NIR775/IR1048) at 755 nm and 905 nm after incubation with •OH and other ROS for 24 h. 1: •OH (200 μM Fe^{2+} + 1 mM H_2O_2); 2: $^1O_2$ (1 mM H_2O_2 + 1 mM ClO^-); 3: 300 μM ^{t}BuOOH; 4: 1 mM ClO^-; 5: 300 μM CuOOH, 6: 1 mM H_2O_2; 7: $O_2^{\bullet-}$ (100 μM xanthine + 22 mU xanthine oxidase); 8: $ONOO^-$ (1 mM $NaNO_2$ + 1 mM H_2O_2); 9: 1-NP alone (Ctrl). Data are presented as mean ± s.d. (n = 3 independent samples). These results together with Fig. 3g-i suggest that 1-NP hold a high specificity against •OH. Source data are provided as a Source Data file.

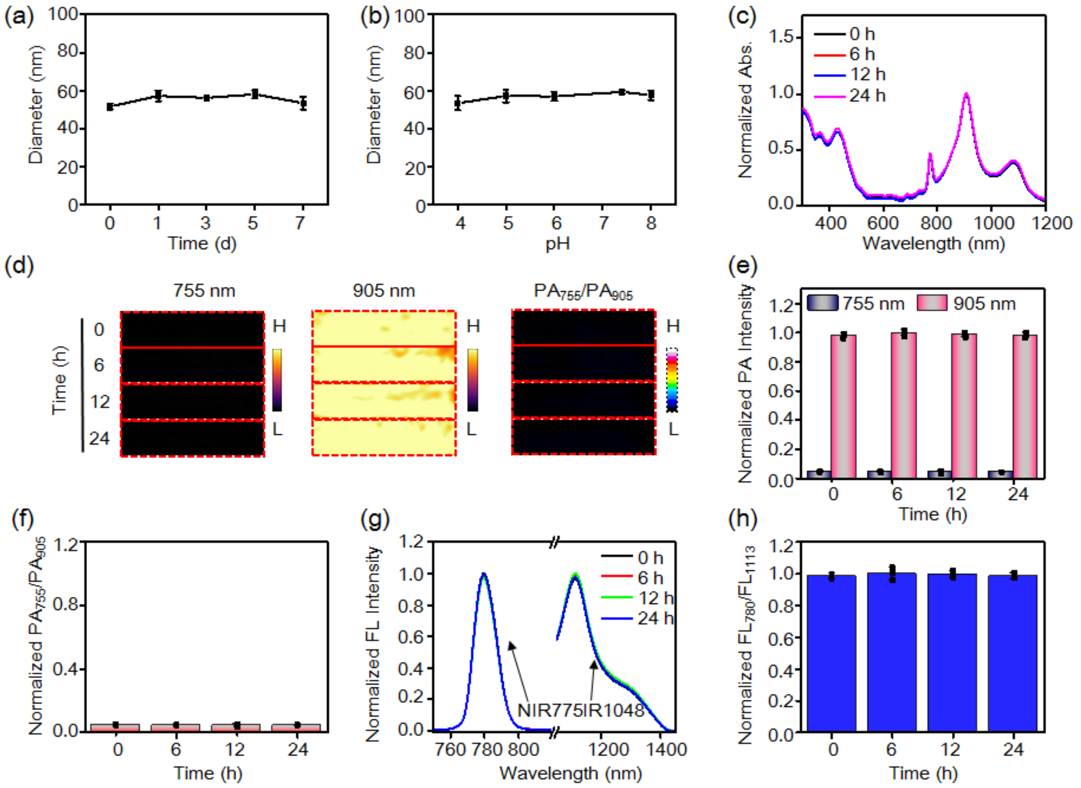

Supplementary Fig. 13 Investigation of the stability of 1-NP under physiological conditions. (a) The hydrodynamic size of 1-NP following incubation in aqueous solution at. r.t. for over 7 days. (b) The hydrodynamic size of 1-NP following incubation in aqueous solution under indicated pH for 24 h. (c) Normalized UV-vis-NIR absorption spectra, (d) Photoacoustic (PA) images, (e) PA intensities, (f) PA_{755}/PA_{905} ratios, (g) Fluorescence (FL) spectra and (h) FL_{780}/FL_{1113} ratios of 1-NP (56/1.65/20 μM 1-Br-Et/NIR775/IR1048) following incubation in aqueous solution for 0, 6, 12 and 24 h. Data are presented as mean ± s.d. (n = 3 independent samples). These results suggest that 1-NP hold a high stability under physiological conditions. Source data are provided as a Source Data file.

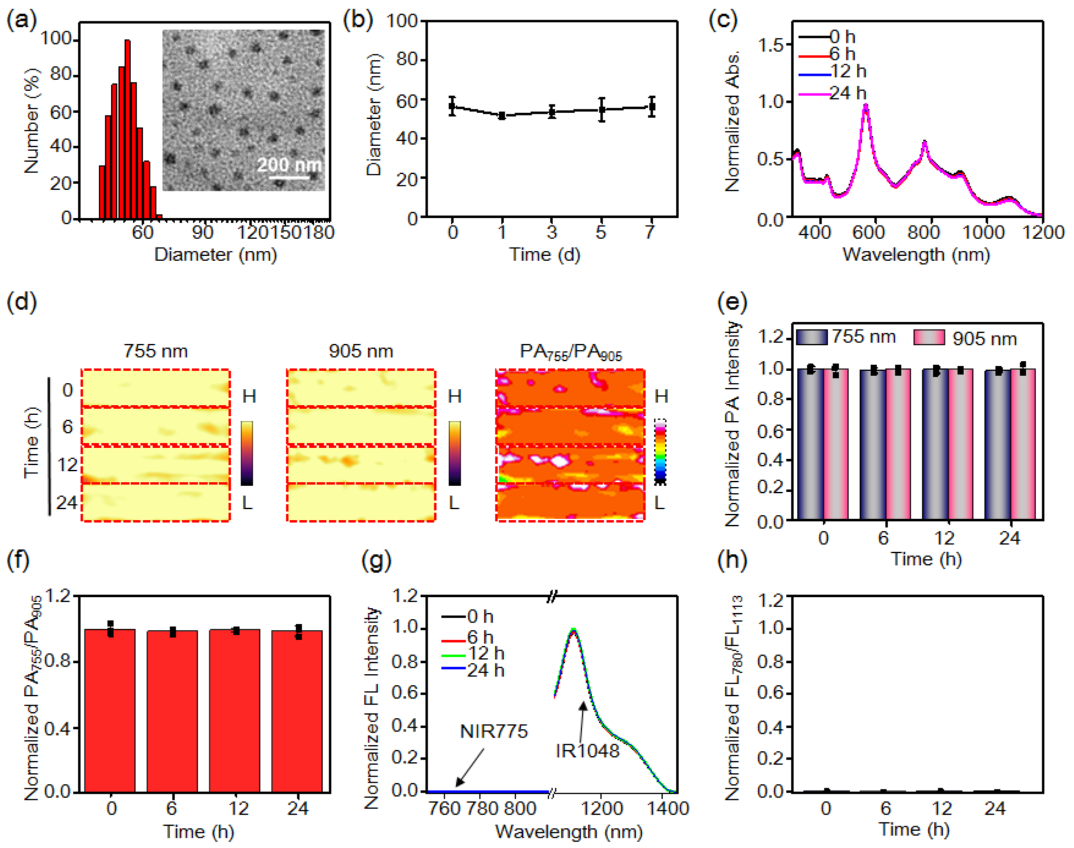

Supplementary Fig. 14 Investigation of the stability of 1-NP after being incubation with •OH under physiological conditions. (a) Dynamic light scattering (DLS) analysis and transmission electron microscopy (TEM) image (inset) of 1-NP after being incubation with •OH. The TEM analysis were repeated independently three times with similar results. (b) The hydrodynamic size of 1-NP in the presence of •OH following incubation in aqueous solution at. r.t. for over 7 days. (c) Normalized UV-vis-NIR absorption spectra, (d) Photoacoustic (PA) images, (e) PA intensities, (f) PA_{755}/PA_{905} ratios, (g) Fluorescence (FL) spectra and (h) FL_{780}/FL_{1113} ratios of 1-NP (56/1.65/20 μM 1-Br-Et/NIR775/IR1048) in the presence of •OH following incubation in aqueous solution for 0, 6, 12 and 24 h. Data are presented as mean ± s.d. (n = 3 independent samples). These results suggest that 1-NP after being incubation with •OH was also stable under physiological conditions. Source data are provided as a Source Data file.

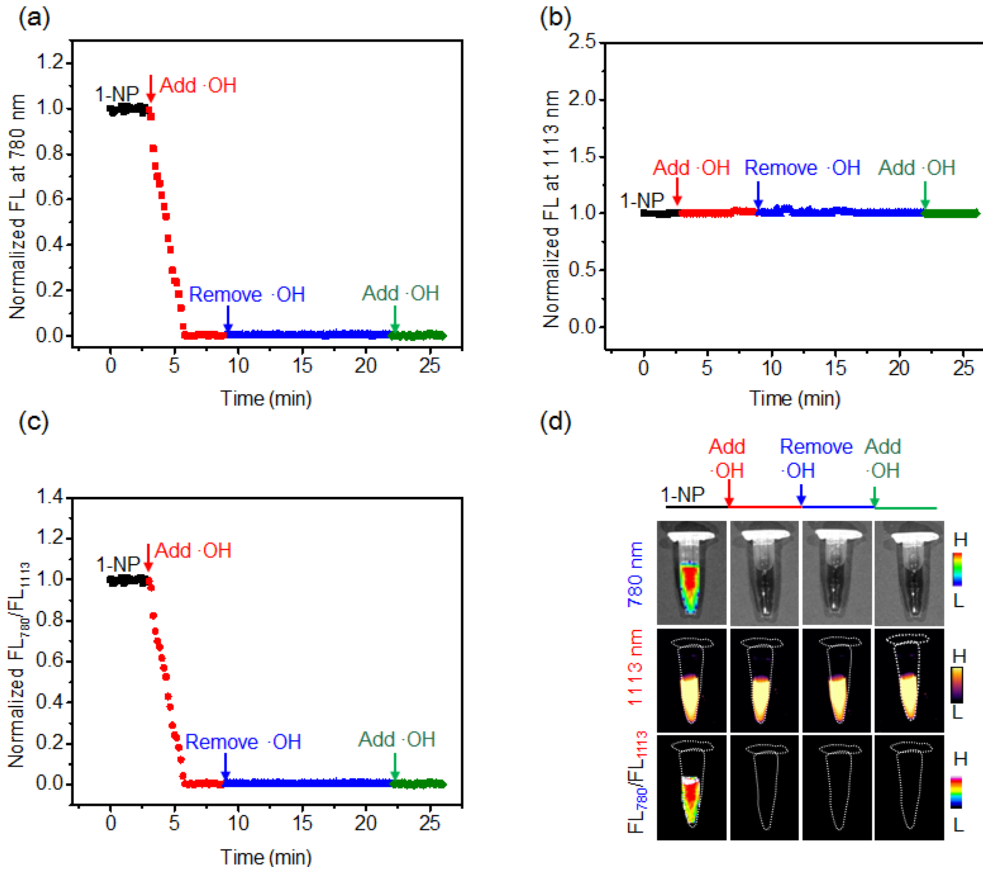

Supplementary Fig. 15 Investigation of reversibility of the reaction between 1-NP and •OH in vitro. Monitoring of the fluorescence (FL) intensities at (a) 780 nm, (b) 1113 nm, and (c) normalized ratiometricc FL_{780}/FL_{1113} ratios of 1-NP upon intermittent addition and removal of •OH. 1-NP in D.I. water was kept at r.t. for 3 min, followed by addition of •OH (200 μM Fe^{2+} + 1 mM H_2O_2); after another 6 min, tempol (2 mM) was added to scavenge the •OH and kept for another 13 min. •OH (200 μM Fe^{2+} + 1 mM H_2O_2) was again added. The fluorescence intensities at 780 and 1113 nm were acquired. (d) FL images and ratiometric FL_{780}/FL_{1113} images of indicated incubation solutions. The results demonstrate the reaction between 1-NP and •OH could not be reversible, matching that 1-NP after being incubation with •OH was stable under physiological conditions (Supplementary Fig. 14).

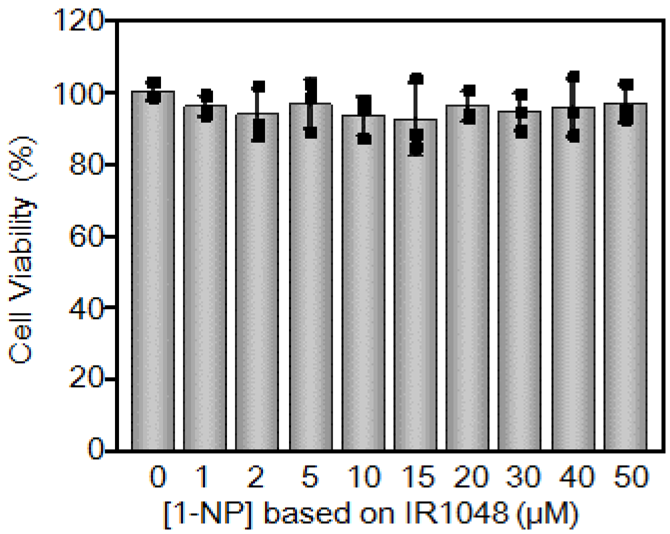

Supplementary Fig. 16 Evaluation of the cytotoxicity of 1-NP against RAW264.7 cells. Cells were incubated with indicated concentration of 1-NP (based on IR1048) for 24 h, and the cell viability was tested by MTT assay. Data are presented as mean ± s.d. (n = 3 independent cell experiment). These findings indicate that 1-NP hold high biocompatibility against RAW264.7 cells. Source data are provided as a Source Data file.

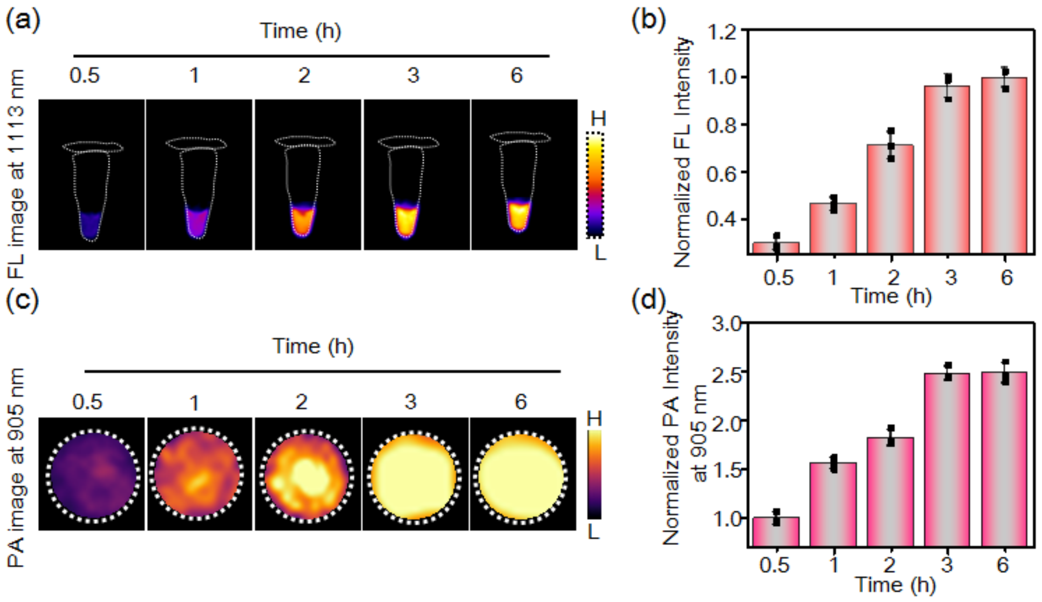

Supplementary Fig. 17 Optimization of incubation time of 1-NP in RAW264.7 cells. (a) NIR-II fluorescence (FL) images, (b) normalized NIR-II FL intensities (at 1113 nm), (c) Photoacoustic (PA) images and (d) normalized PA intensities (at 905 nm) of RAW264.7 cells (~5×10^5 cells) incubated with 1-NP (56/1.65/20 μM 1-Br-Et/NIR775/IR1048) for 0.5, 1, 2, 3 and 6 h. Data are presented as mean ± s.d. (n = 3 independent cell pellets). Source data are provided as a Source Data file.

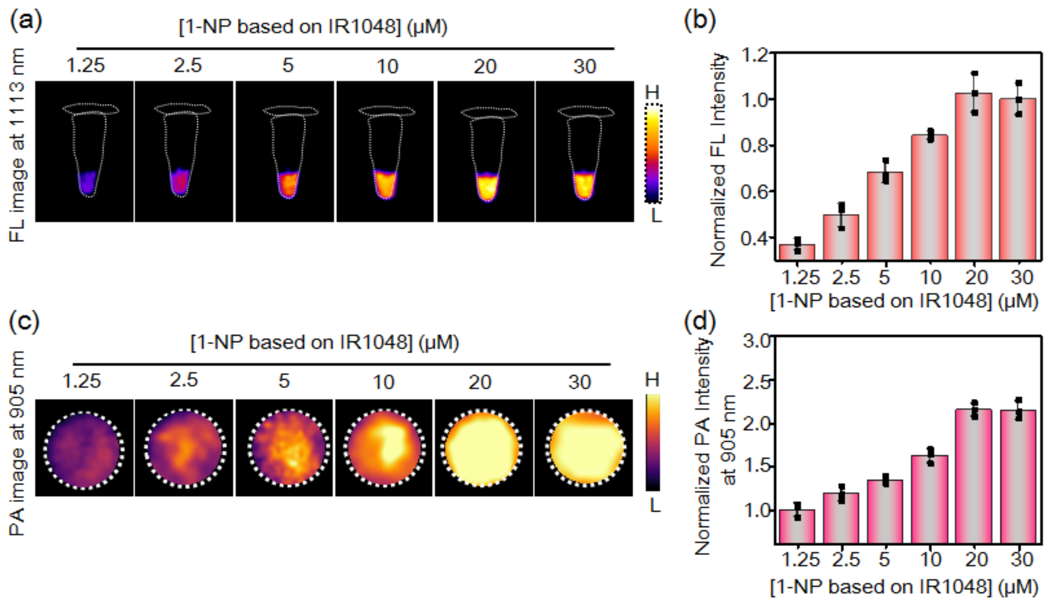

Supplementary Fig. 18 Optimization of incubation concentration of 1-NP in RAW264.7 cells. (a) NIR-II fluorescence (FL) images, (b) normalzied NIR-II FL intensities (at 1113 nm), (c) Photoacoustic (PA) images and (d) normalized PA intensities (at 905 nm) of RAW264.7 cells (~5×10^5 cells) incubated with different concentration of 1-NP (based on the concentration of IR1048) for 3 h. Data are presented as mean ± s.d. (n = 3 independent cell pellets). Source data are provided as a Source Data file.

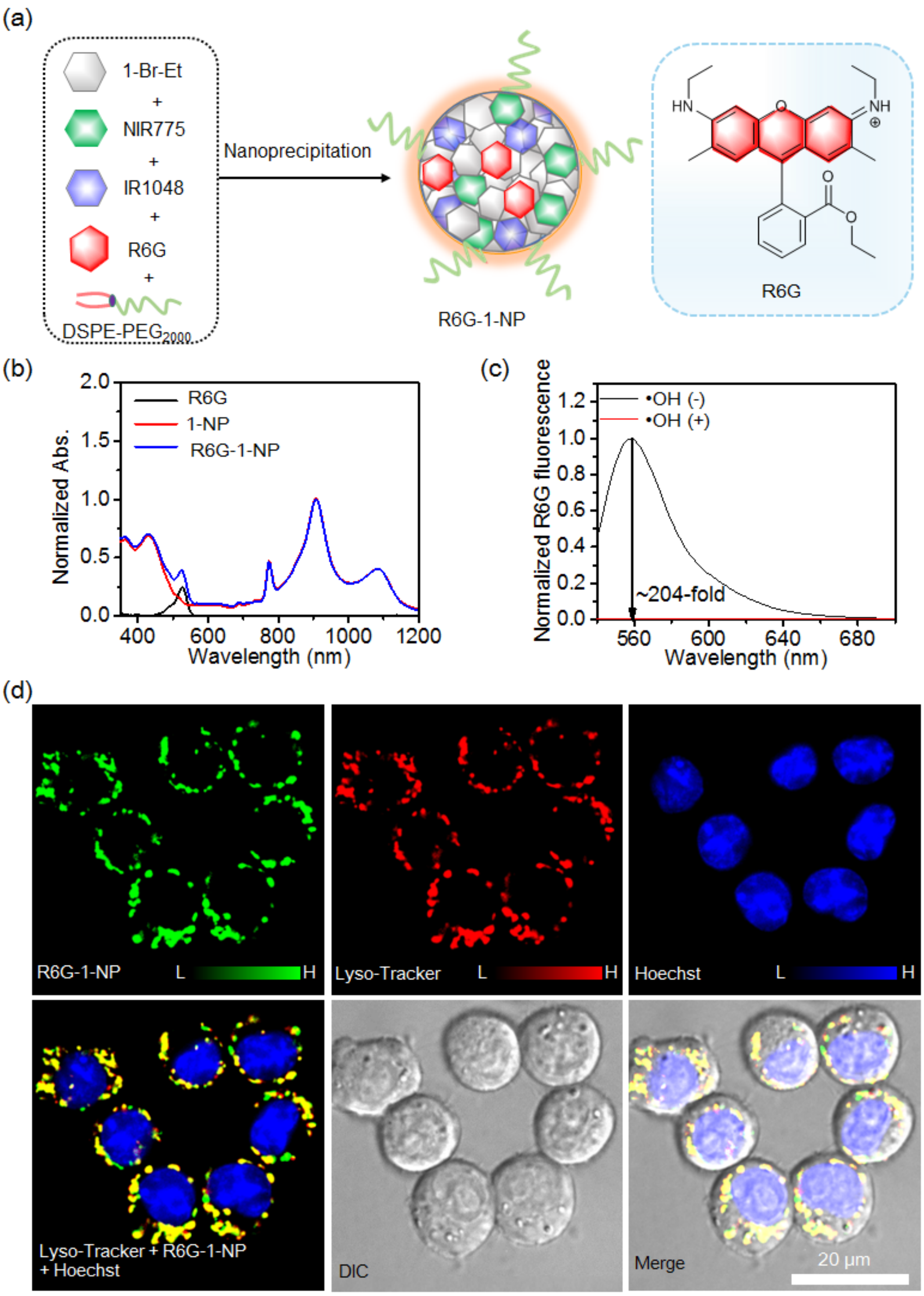

Supplementary Fig. 19 Investigation of the intracellular distribution of 1-NP. (a) To accommodate epifluorescence imaging for colocalization analysis, Rhodamine 6G (R6G)-1-NP was prepared by loading of R6G into 1-NP via the same nanoprecipitation approach. (b) UV-Vis-NIR absorption spectra of R6G-1-NP, R6G and 1-NP. (c) Fluorescence (FL) spectra of

R6G-1-NP (56/1.65/20/2 μM 1-Br-Et/NIR775/IR1048/R6G) following incubation with •OH (200 μM Fe^{2+} + 1 mM H_2O_2). (d) Colocalization study of macrophages incubating with R6G-1-NP (green) and Lyso-tracker (red). Macrophages were pretreated with R6G-1-NP (56/1.65/20/2 μM 1-Br-Et/NIR775/IR1048/R6G, 3 h) and co-stained with Lyso-tracker red (1 μM, 20 min). In order to study the colocalization of 1-NP in RAW264.7 cells, we encapsulated R6G (2 μM) in 1-NP to prepare R6G-1-NP. The colocalization studies with a lysosome staining dye (Lyso-tracker red) demonstrated that the intracellular fluorescence matched well with that of Lyso-tracker, indicating that R6G-1-NP localized primarily in lysosomes. The colocalization images shown in (d) are representative of three independent experiments.

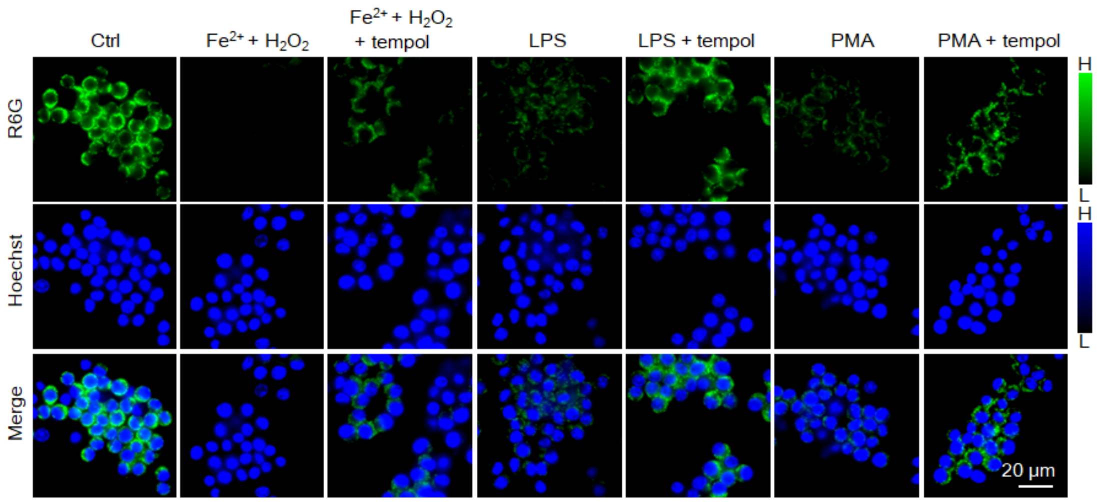

Supplementary Fig. 20 Fluorescence imaging of intracellular •OH using R6G-1-NP. Fluorescence images of R6G in RAW264.7 cells upon treatment with R6G-1-NP (56/1.65/20 μM 1-Br-Et/NIR775/IR1048) for 3 h plus different conditions. 1: Ctrl; 2: 200 μM Fe^{2+} + 1 mM H_2O_2; 3: 200 μM Fe^{2+} + 1 mM H_2O_2 + tempol (200 μM); 4: LPS (20 μg/mL); 5: LPS (20 μg/mL) + tempol (200 μM); 6: PMA (20 μg/mL); 7: PMA (20 μg/mL) + tempol (200 μM). The cell imaging experiments were repeated independently three times with similar results.

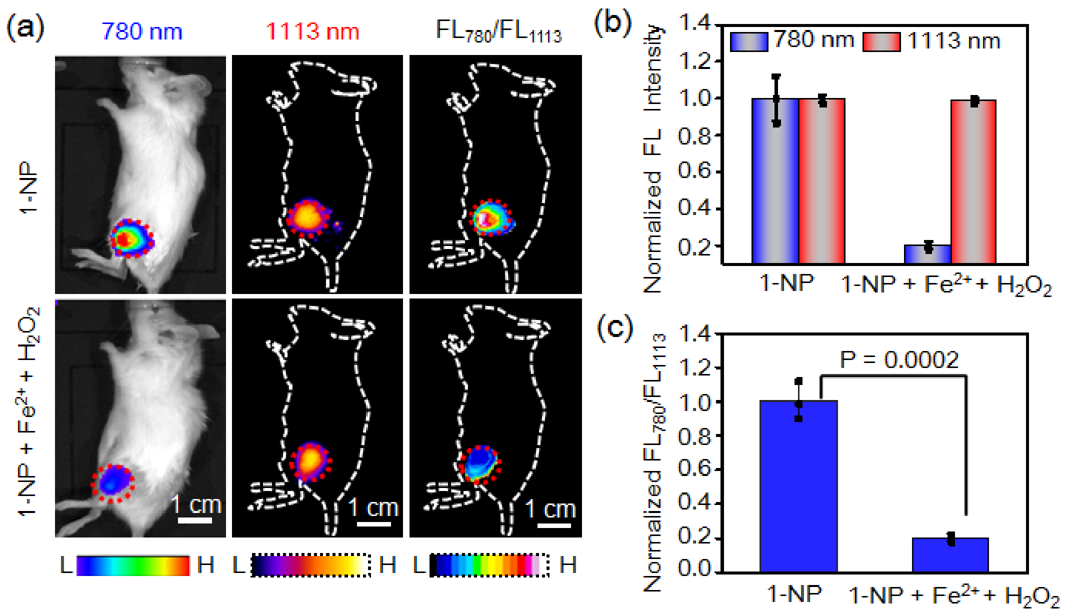

Supplementary Fig. 21 Fluorescence imaging of exogenous •OH in living mice. (a) Representative FL images and ratiometric FL_{780}/FL_{1113} images of mice with subcutaneous injection of 1-NP (112/3.3/40 μM 1-Br-Et/NIR775/IR1048) or 1-NP plus •OH (200 μM Fe^{2+} + 1 mM H_2O_2). Red circles indicate the injection sites in mice. (b) Quantitative analysis of the FL intensities of at both 780 nm and 1113 nm in the injection sites containing 1-NP with or without the Fenton reagents. (c) Normalized FL_{780}/FL_{1113} ratios in the injection sites of mice following indicated treatment. Data are presented as mean ± s.d. (n = 3 independent mice). Statistical differences were analyzed by Student's two-sided t-test. Source data are provided as a Source Data file.

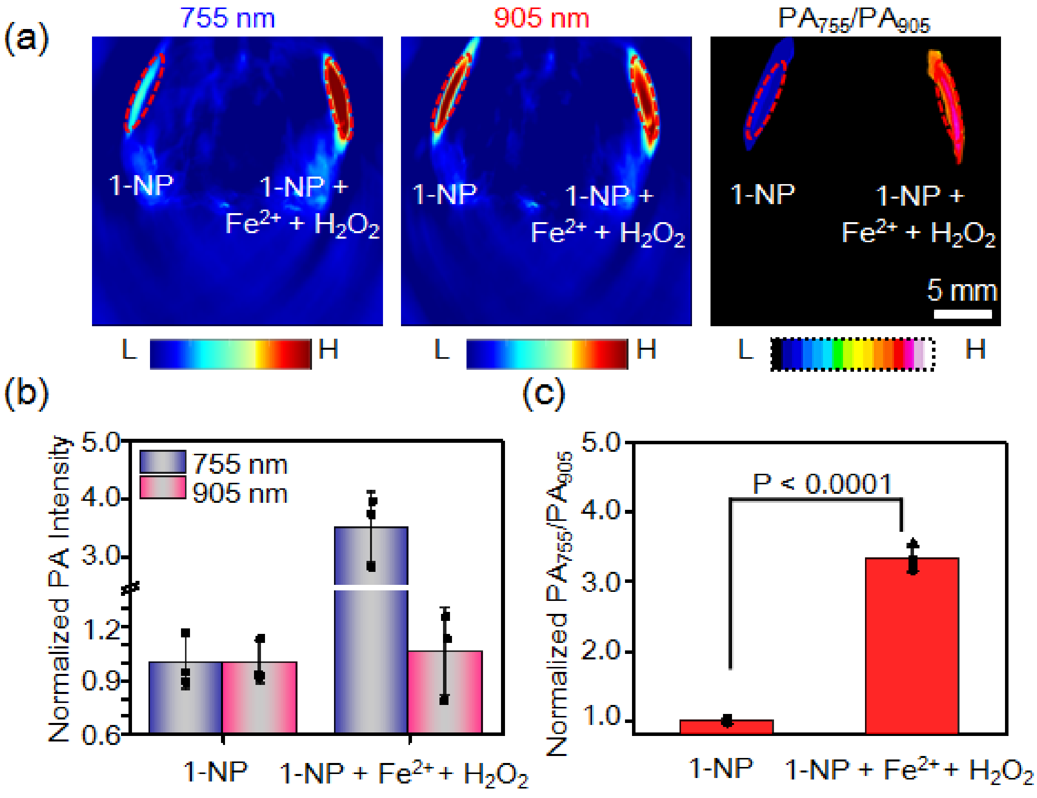

Supplementary Fig. 22 Photoacoustic (PA) imaging of exogenous •OH in vivo. (a) Representative PA and ratiometric PA_{755}/PA_{905} images of mice with subcutaneous injection of 1-NP (112/3.3/40 μM 1-Br-Et/NIR775/IR1048, Ctrl) and 1-NP plus •OH (200 μM Fe^{2+} + 1 mM H_2O_2). Red circles indicate the injection sites containing 1-NP with or without the Fenton reagents. Quantitative analysis of average PA intensities at 755 nm (b) and 905 nm (c) following indicated treatment. (d) Normalized PA_{755}/PA_{905} ratios in the injection sites of mice following indicated treatment. Data are presented as mean ± s.d. (n = 3 independent mice). Statistical differences were analyzed by Student's two-sided t-test. Source data are provided as a Source Data file.

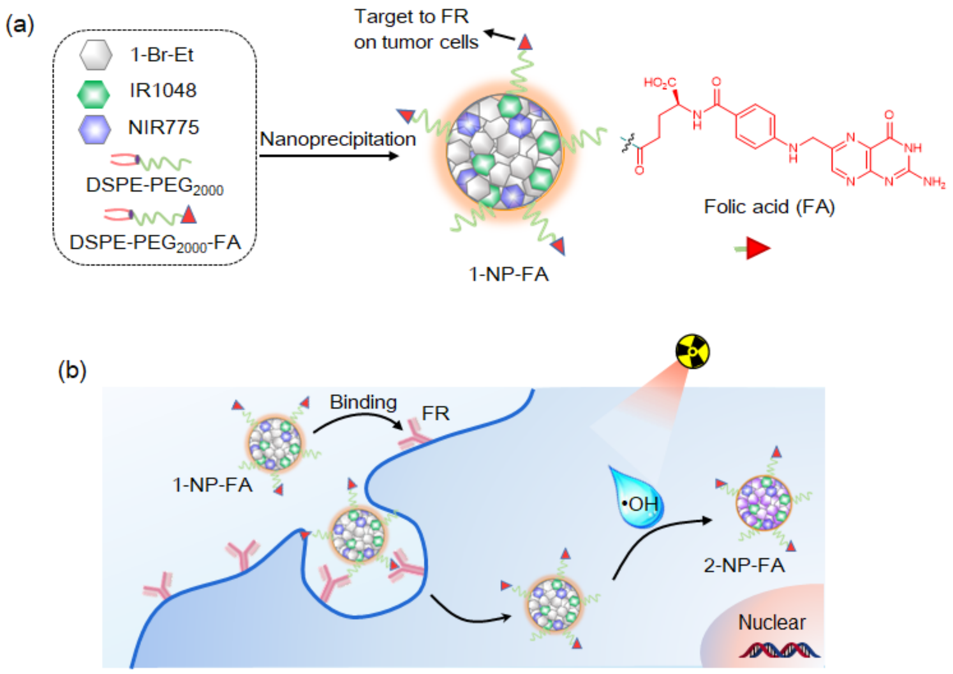

Supplementary Fig. 23 Schematic illustration of the design of 1-NP-FA. (a) Preparation of 1-NP-FA. (b) Schematic illustration of ratiometric fluorescence/photoacoustic (FL/PA) imaging of •OH in tumor cells using 1-NP-FA. 1-NP-FA enters into tumor cells via folate (FA)-mediated endocytosis; the elevated •OH levels in tumor cells following therapy (radiotherapy (RT) or erastin-induced ferroptosis) can subsequently react with it, resulting in a decreased FL_{780}/FL_{1113} ratio while an increased PA_{755}/PA_{905} ratio.

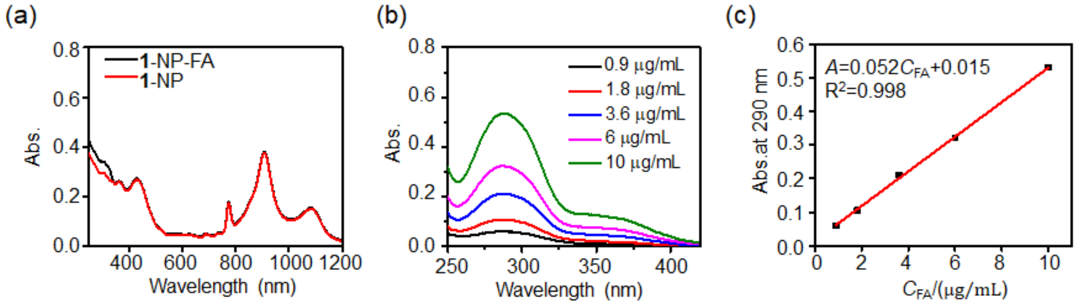

Supplementary Fig. 24 Calculation of the mass ratio of folate (FA), 1-Br-Et, NIR775 and IR1048 within 1-NP-FA. (a) UV-Vis-NIR absorption spectra of 1-NP-FA and 1-NP (28/0.825/10 μM 1-Br-Et/NIR775/IR1048) in water. (b) UV-Vis-NIR absorption spectra varying concentrations of FA in water. (c) Plot of the absorption intensities at 290 nm versus the concentration of FA.

Supplementary Note 1: Calculation of the mass ratio of folate (FA), 1-Br-Et, NIR775 and IR1048 within 1-NP-FA.

According to the UV-vis-NIR absorption spectra of 1-NP and 1-NP-FA (28/0.825/10 μM 1-Br-Et/NIR775/IR1048) in (a), the absorption of loading FA at 290 nm could be obtained by subtracting the UV absorption of 1-NP-FA to that of 1-NP at 290 nm:

$A_{1\text{-NP-FA}} - A_{1\text{-NP}} = 0.339 - 0.295 = 0.044$

According to the standard curve ($A = 0.052 \times C_{FA} + 0.015$) of the UV absorption of FA at 290 nm versus the concentration of free FA in water, where A is the UV absorption of FA at 290 nm and C_{FA} is the concentration of FA (μg/mL).

Accordingly, the concentration of FA within 1-NP-FA (28/0.825/10 μM 1-Br-Et/NIR775/IR1048) could be estimated using the follow equation:

$C_{FA} = (0.044 - 0.015)/0.052 = 0.56\ \mu g/mL$

Therefore, the concentration of FA to 1-Br-Et, IR1048 and NIR775 in the 1-NP-FA could be found to be: 0.56 μg/mL to 28 μM 1-Br-Et (29 μg/mL), 0.825 μM NIR775 (1.1 μg/mL) and 10 μM IR1048 (7.2 μg/mL), which was equal to the ratio of 0.01/0.52/0.02/0.13 (FA/1-Br-Et/NIR775/IR1048) by mass.

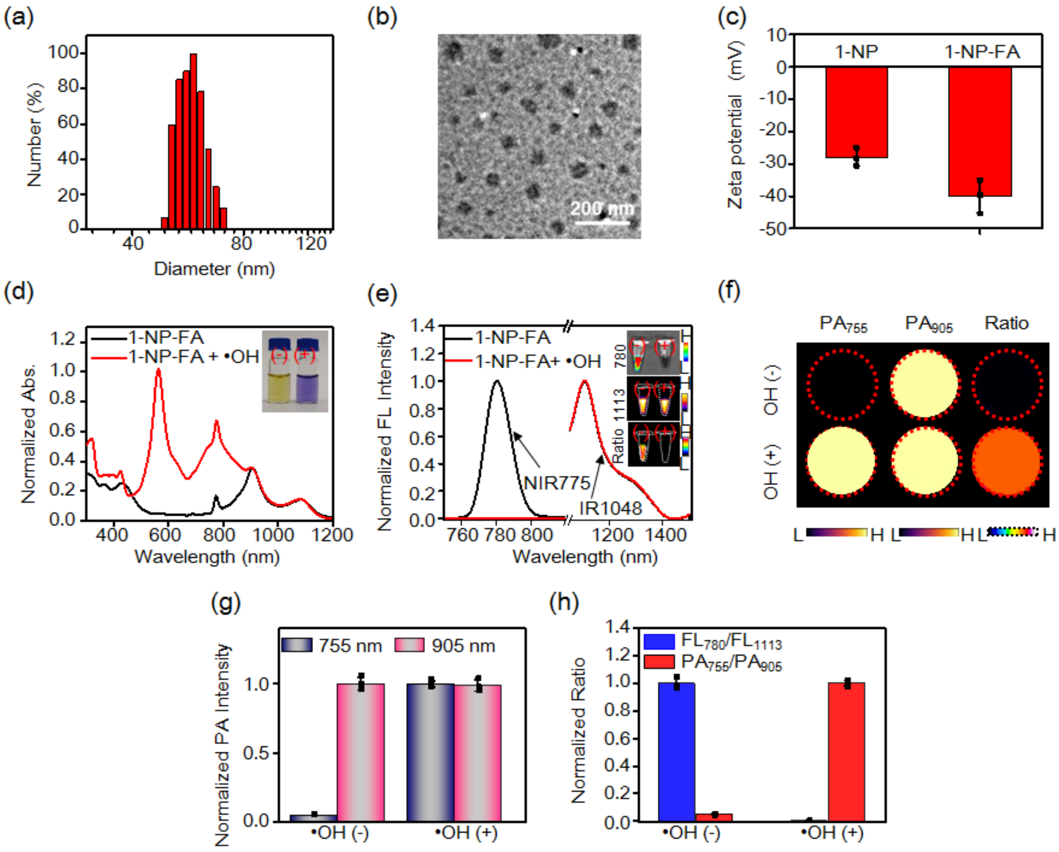

Supplementary Fig. 25 Characterization of 1-NP-FA in vitro. (a) Dynamic light scattering (DLS) analysis and (b) transmission electron microscopy (TEM) image of 1-NP-FA. The TEM analysis were repeated independently three times with similar results. (c) Zeta potential and (d) UV-Vis-NIR absorption spectra of 1-NP-FA and 1-NP in water. (e) Normalized UV-Vis-NIR absorption spectra and photograph (inset) of 1-NP-FA before and after incubation with •OH (200 μM Fe^{2+} + 1 mM H_2O_2) at r.t. for 3 min. (f) Fluorescence (FL) spectra, FL images and ratiometric FL_{780}/FL_{1113} images (inset) of 1-NP-FA (56/1.65/20 μM 1-Br-Et/NIR775/IR1048) before and after incubation with •OH (200 μM Fe^{2+} + 1 mM H_2O_2) at r.t. for 3 min. (g) Photoacoustic (PA) images and ratiometric PA_{755}/PA_{905} images, and (h) normalized PA intensities of 1-NP-FA (56/1.65/20 μM 1-Br-Et/NIR775/IR1048) at 755 and 905 nm before and after incubation with •OH (200 μM Fe^{2+} + 1 mM H_2O_2) at r.t. for 3 min. (i) Normalized FL_{780}/FL_{1113} and PA_{755}/PA_{905} ratios of 1-NP-FA (56/1.65/20 μM 1-Br-Et/NIR775/IR1048) before and after incubation with •OH (200 μM Fe^{2+} + 1 mM H_2O_2) at r.t. for 3 min. Data are presented as mean ± s.d. (n = 3 independent samples). Source data are provided as a Source Data

file.

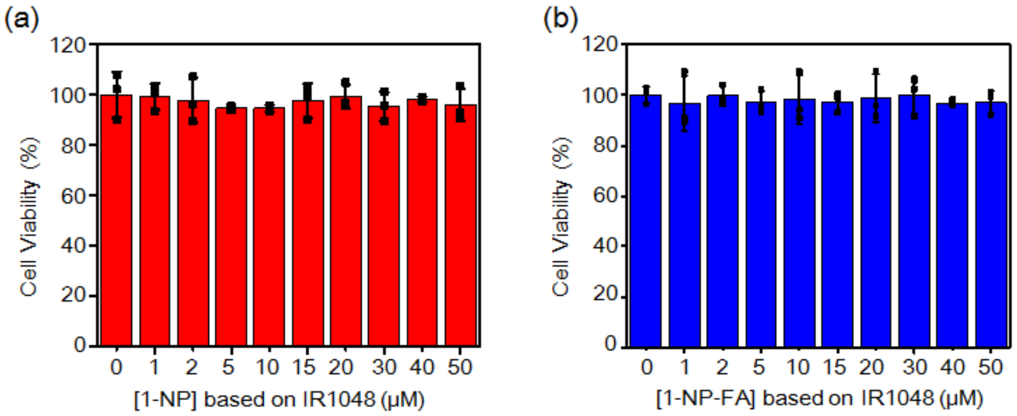

Supplementary Fig. 26 Evaluation of the biocompatibility of 1-NP or FA-1-NP in 4T1 cells. The cell viability of (a) 1-NP and (b) 1-NP-FA against 4T1 cells. 4T1 cells were incubated with indicated concentration of 1-NP or 1-NP-FA (based on IR1048) for 24 h, and the cytotoxicity was tested by MTT assay. Data are presented as mean ± s.d. (n = 3 independent cell experiment). These results suggest that both 1-NP and 1-NP-FA hold high biocompatibility against 4T1 tumor cells. Source data are provided as a Source Data file.

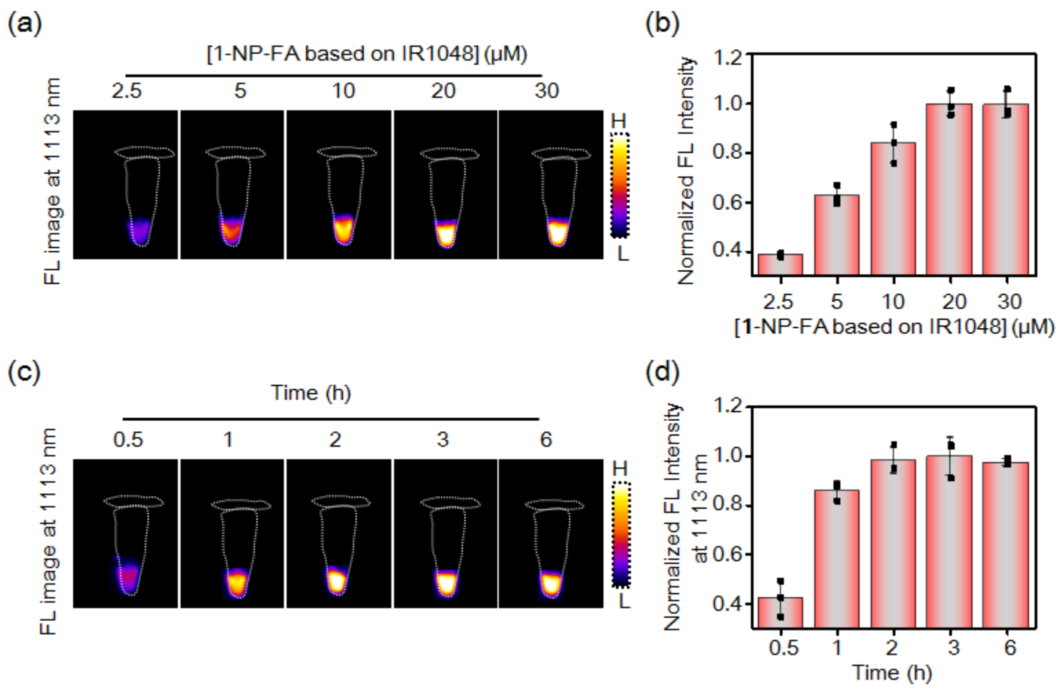

Supplementary Fig. 27 Optimization of the incubation concentration and time of 1-NP-FA in 4T1 cells. (a) NIR-II fluorescence (FL) images and (b) normalized NIR FL intensities of 4T1 cells incubated with different concentration of 1-NP-FA (based on the concentration of IR1048) for 2 h. (c) NIR-II FL images and (d) normalized NIR-II FL intensities of 4T1 cells incubated with 1-NP-FA (20 μM IR1048) for different time. Data are presented as mean ± s.d. (n = 3 independent cell pellets). Source data are provided as a Source Data file.

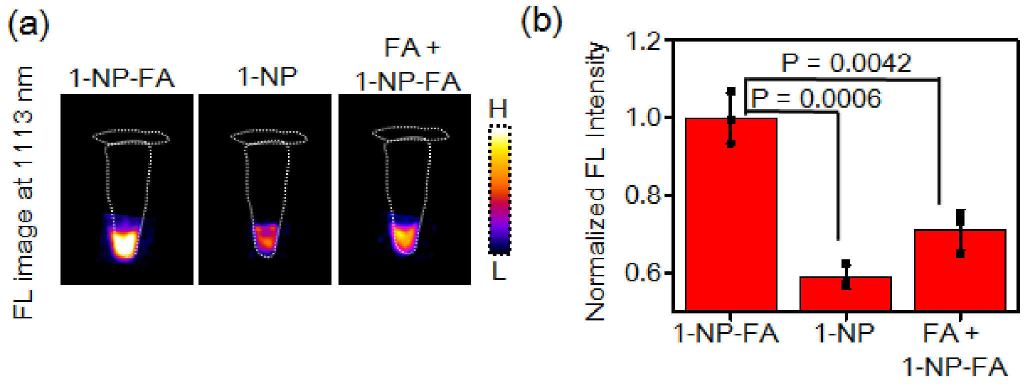

Supplementary Fig. 28 Investigation of the uptake of 1-NP-FA in 4T1 cells. (a) NIR-II fluorescence (FL) images and (b) normalized NIR-II FL intensities of 4T1 cells after being incubation with 1-NP-FA, 1-NP or 1-NP-FA together with 1 mM free folate (FA) for 2 h. The concentration of 1-NP-FA and 1-NP were 20 μM based on IR1048. Data are presented as mean ± s.d. (n = 3 independent cell pellets). Statistical differences were analyzed by Student's two-sided t-test. The results revealed that 1-NP-FA hold an enhanced ability to enter 4T1 cells, as compared with 1-NP, which could be prohibited by free FA, suggesting an important role of FA ligands on 1-NP-FA in enhancing cellular uptake. Source data are provided as a Source Data file.

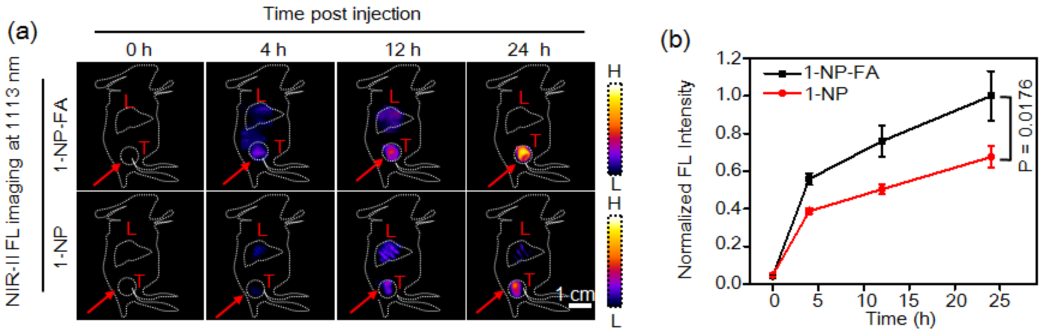

Supplementary Fig. 29 Time-dependent NIR-II fluorescence (FL) imaging of 4T1 tumor-bearing mice. (a) NIR-II FL images and (b) normalized NIR-II FL intensities of 4T1 tumors at 0, 4, 12 and 24 h post i.v. injection of 1-NP-FA or 1-NP (1.68/0.05/0.6 mM 1-Br-Et/NIR775/IR1048, 200 μL in saline). Red arrow indicates the location of tumor (T) and L represent the location of liver in the mouse. Data are presented as mean ± s.d. (n = 3 independent mice). Statistical differences were analyzed by Student's two-sided t-test. The in vivo NIR-II FL imaging results showed that both 1-NP-FA and 1-NP were preferentially accumulated in 4T1 tumors after 24 h, and 1-NP-FA was more efficient to enter into 4T1 tumors than 1-NP. Source data are provided as a Source Data file.

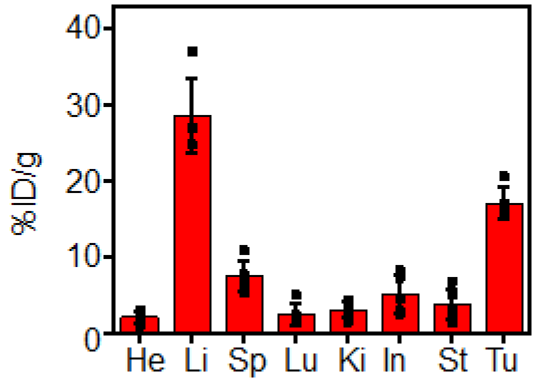

Supplementary Fig. 30 Biodistribution of 1-NP-FA (1.68/0.05/0.6 mM 1-Br-Et /NIR775/IR1048, 200 μL) at 24 h post i.v. injection into 4T1 tumor-bearing mice. Data are presented as mean ± s.d. (n = 5 independent mice). Source data are provided as a Source Data file.

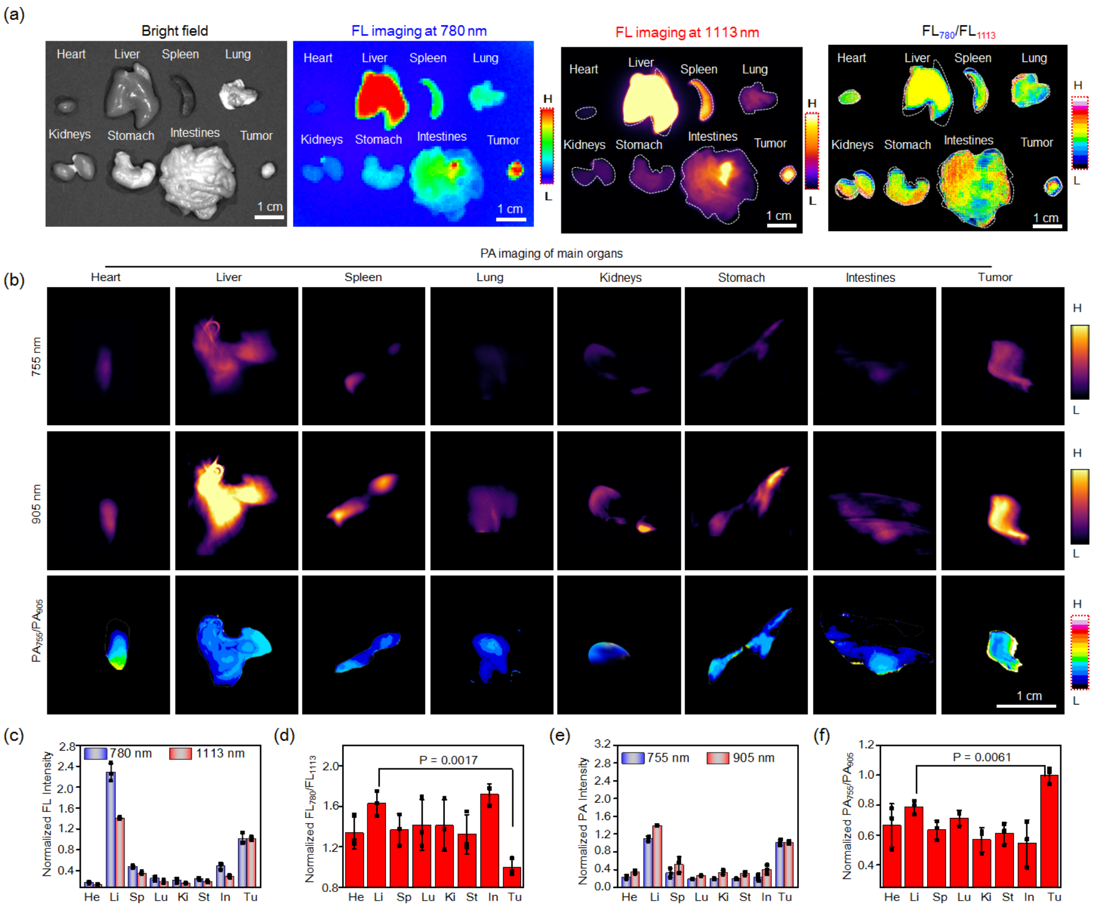

Supplementary Fig. 31 Ex vivo fluorescence and photoacoustic imaging of 4T1 tumors-bearing mice. Representative ex vivo fluorescence (FL) (780 and 1113 nm), ratiometric FL_{780}/FL_{1113} images, photoacoustic (PA) (755 and 905 nm) and ratiometric PA_{755}/PA_{905} images of main organs (e.g., liver (Li), lung (Lu), heart (He), kidneys (Ki), intestines (In), stomach (St), spleen (Sp) and tumor (Tu)) resected from 4T1 tumors-bearing mice with i.v. injection of 1-NP-FA. (a) Bright field, FL (780 and 1113 nm) and FL_{780}/FL_{1113} images of main organs. (b) PA (755 and 905 nm) and PA_{755}/PA_{905} images of main organs. (c) FL (780 and 1113 nm) and (d) FL_{780}/FL_{1113} intensities of main organs. (e) PA (755 and 905 nm) and (f) PA_{755}/PA_{905} intensities of main organs. Data are presented as mean ± s.d. (n = 3 independent mice). Statistical differences were analyzed by Student's two-sided t-test. Source data are provided as a Source Data file.

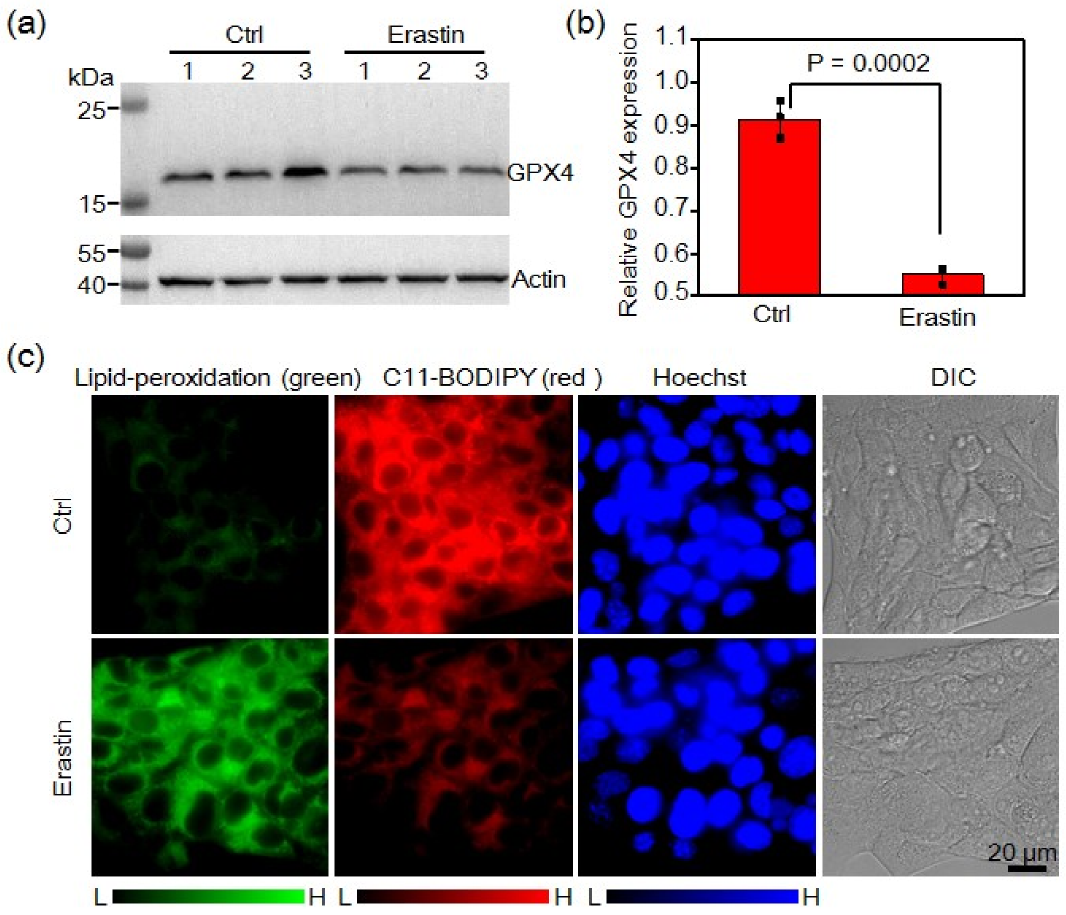

Supplementary Fig. 32 Examination of erastin-induced ferroptosis in 4T1 cells. (a) Western blotting (WB) analysis and (b) comparison of the relative GPX4 protein levels showed that GPX4 expression level in 4T1 cells upon treatment with erastin was downregulated compared to that of control cells. Data are presented as mean ± s.d. (n = 3 independent cells). Statistical differences were analyzed by Student's two-sided t-test. (c) Fluorescence imaging of lipid peroxides in control and erastin-treated 4T1 cells following staining with 10 μM C11-BODIPY for 30 min. For (a) and (c), the experiments were repeated independently three times with similar results. Source data are provided as a Source Data file.

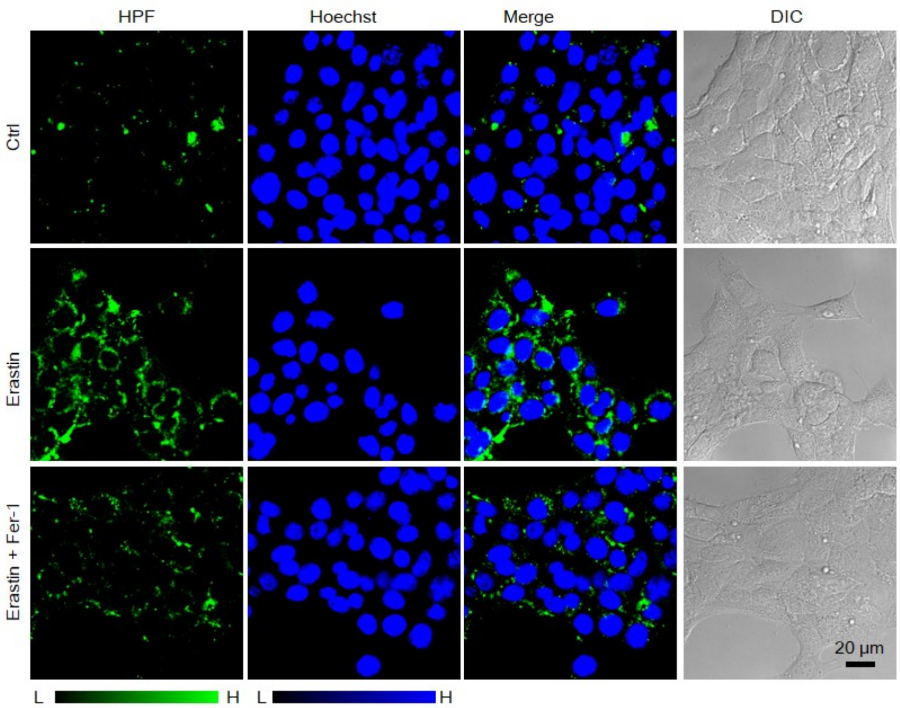

Supplementary Fig. 33 Fluorescence images of •OH by hydroxyphenyl fluorescein (HPF) in control 4T1 cells (Ctrl) or 4T1 cells upon incubation with erastin or erastin plus Fer-1. Cells were untreated (Ctrl), or pretreated with 10 μM erastin to induce ferroptosis, or 10 μM erastin plus 10 μM Fer-1 for 7.5 h, and then incubated with HPF (20 μM) for another 0.5 h. Fluorescence of HPF was obviously increased in erastin-induced ferroptotic cells compared with that of untreated with cells, indicating efficient intracellular •OH generation in erastin-treated tumor cells. However, HPF's fluorescence was significant dim in cells incubated with Fer-1, suggesting that Fer-1 could suppress •OH production. The experiments were repeated independently three times with similar results.

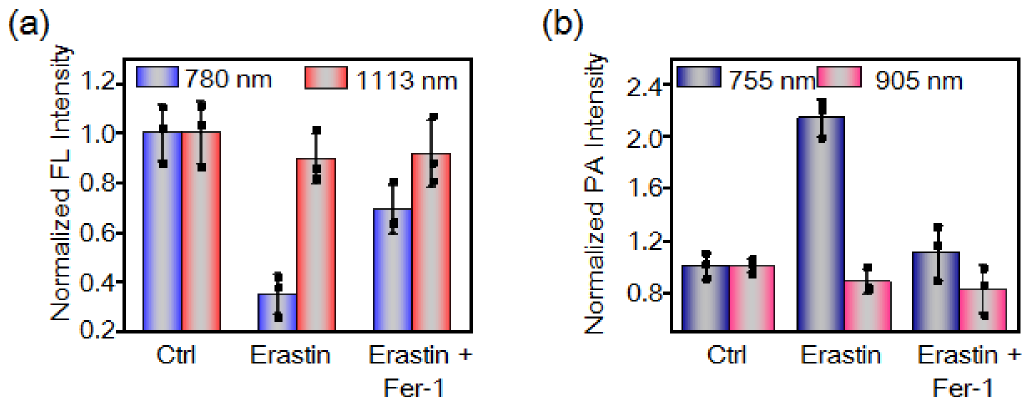

Supplementary Fig. 34 Fluorescence (FL) and photoacoustic (PA) intensities of 4T1 cells following incubation with 1-NP-FA (Ctrl), 1-NP-FA together with erastin or erastin + Fer-1. (a) Normalized FL intensities and (b) normalized PA intensities of 4T1 cells following incubation with 1-NP-FA (Ctrl), 1-NP-FA together with erastin or erastin + Fer-1. Cells were pretreated with 10 μM erastin with or without 10 μM Fer-1 for 6 h, and then incubated with 1-NP-FA (56/1.65/20 μM 1-Br-Et/NIR775/IR1048) for another 2 h. The representative FL and PA images of 4T1 cell pellets were shown in Fig. 5a&5b. Data are presented as mean ± s.d. (n = 3 independent cell pellets). Source data are provided as a Source Data file.

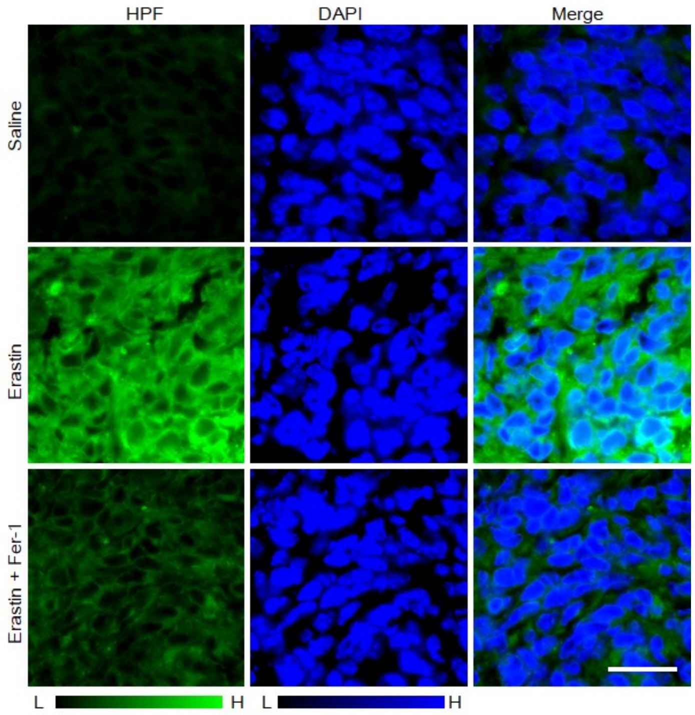

Supplementary Fig. 35 Fluorescence (FL) staining of 4T1 tumor tissue slices using hydroxyphenyl fluorescein (HPF). Mice bearing 4T1 tumors were i.p. injected with saline, erastin (20 mg/kg), or erastin (20 mg/kg) plus Fer-1 (20 mg/kg). Scale bar: 20 μm. The results showed a much brighter HPF fluorescence in erastin-treated ferroptotic mice compared to that of saline-treated control mice, which could be suppressed by Fer-1, a ferroptosis inhibitor, revealing that the •OH level was upregulated in erastin-treated ferroptotic tumor tissues. The experiments were repeated independently three times with similar results.

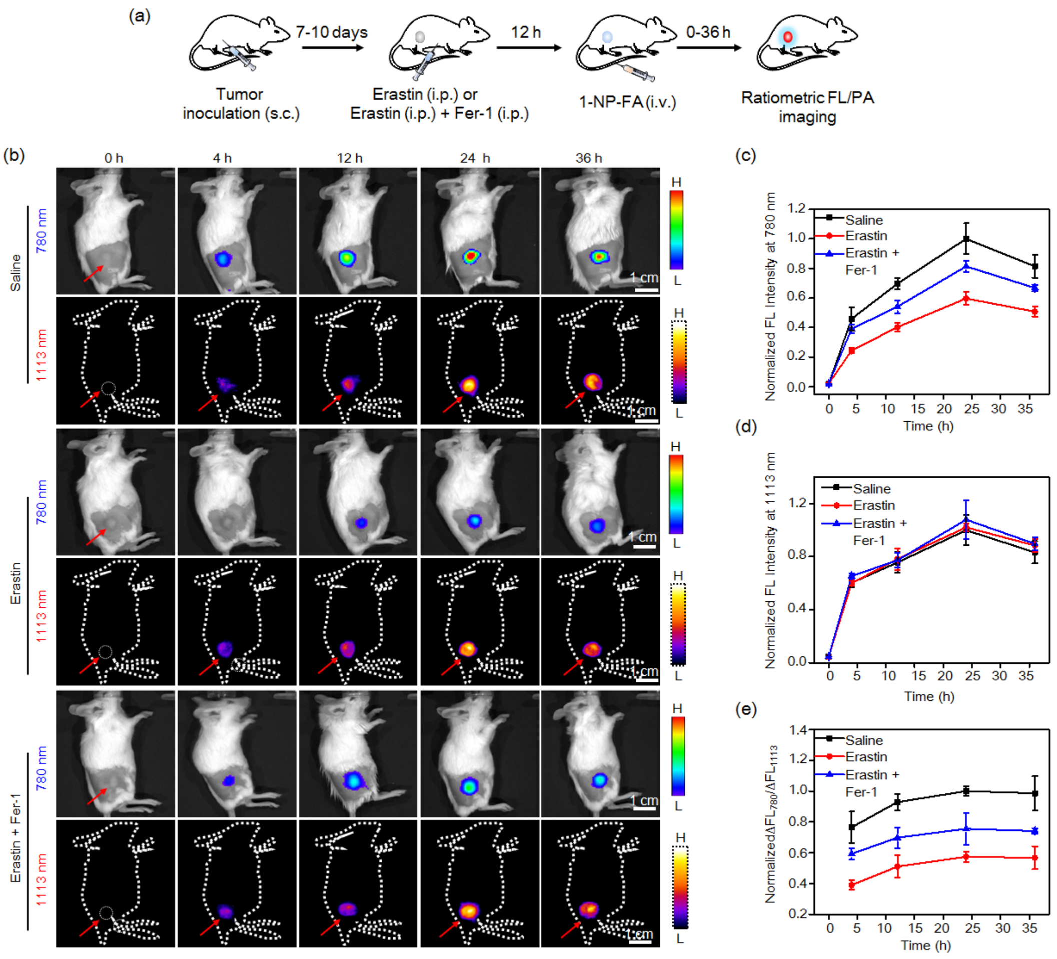

Supplementary Fig. 36 Longitudinal fluorescence (FL) imaging of •OH generation in 4T1 tumor bearing mice. (a) Schematic for longitudinal ratiometric fluorescence/photoacoustic (FL/PA) bimodality imaging of •OH in 4T1 tumor-bearing mice upon induction of ferroptosis by erastin. (b) Longitudinal FL images of 4T1 tumors in mice at different time post injection of 1-NP-FA. 4T1 tumor bearing mice were i.p. injected with saline, erastin (20 mg/kg) or erastin (20 mg/kg) plus Fer-1 (20 mg/kg), and 12 h later, 1-NP-FA (1.68/0.05/0.6 mM 1-Br-Et/NIR775/IR1048, 200 μL) were i.v. injected. The FL images at both 780 and 1113 nm were acquired at 0. 4, 12, 24 and 36 h. Normalized tumor FL intensities at 780 nm (c) and 1113 nm (d) at indicated time point. (e) Normalized $\Delta FL_{780}/\Delta FL_{1113}$ ratio in tumors at indicated time point. Data are presented as mean ± s.d. (n = 3 independent mice). Source data are provided as a Source Data file.

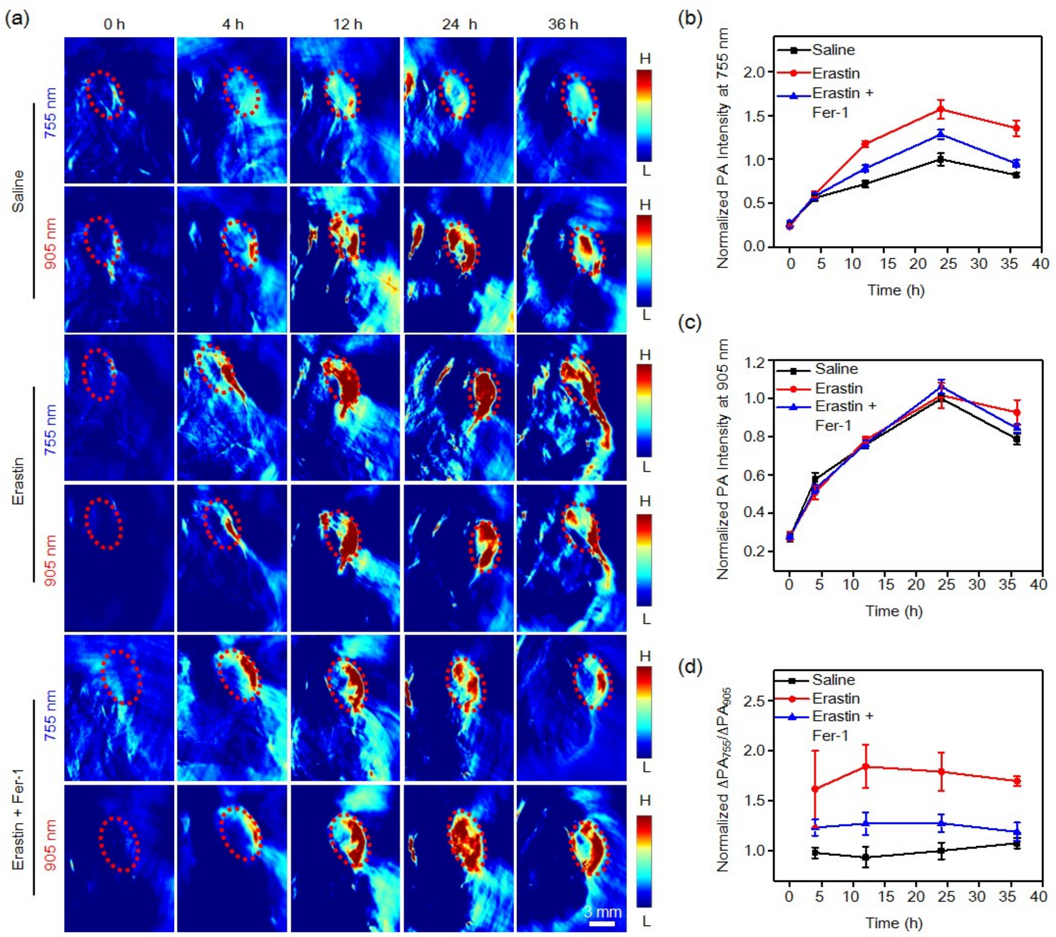

Supplementary Fig. 37 Longitudinal photoacoustic (PA) imaging of •OH generation in 4T1 tumor bearing mice. (a) Longitudinal PA images of 4T1 tumors in mice at different time post injection of 1-NP-FA. Normalized tumor PA intensities at 755 nm (b) and 905 nm (c) at indicated time point. (d) Normalized $\Delta PA_{755}/\Delta PA_{905}$ ratio in 4T1 tumors at indicated time point. Data are presented as mean ± s.d. (n = 3 independent mice). Source data are provided as a Source Data file.

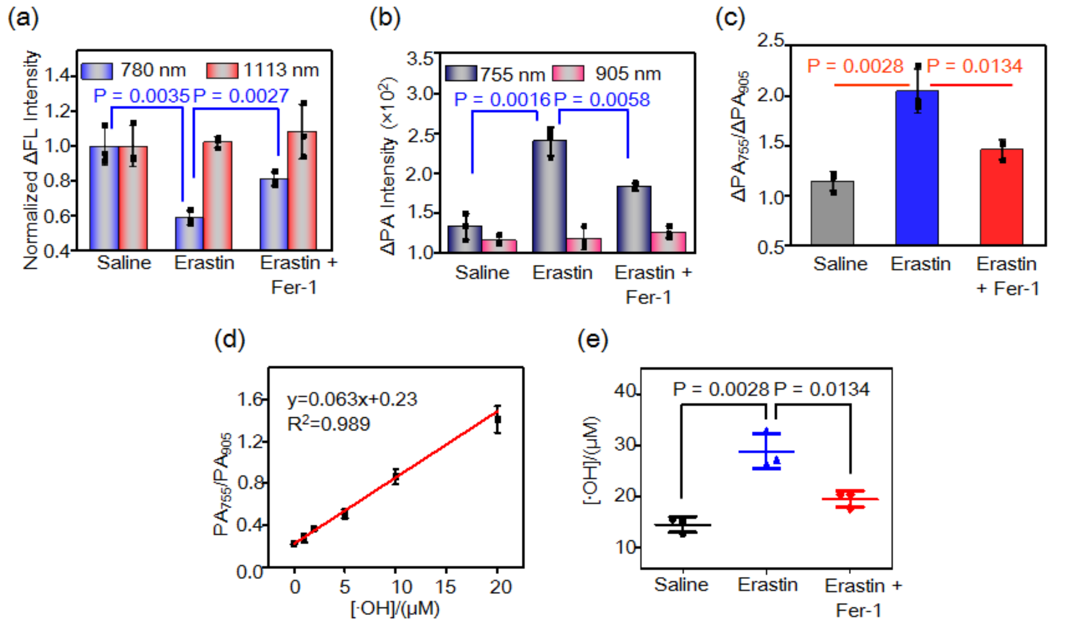

Supplementary Fig. 38 Ratiometric photoacoustic (PA) imaging of •OH in 4T1 tumors undergoing ferroptosis. Fluorescence (FL) intensity increment ΔFL (a), PA intensity increment ΔPA (b) intensities and $\Delta PA_{755}/\Delta PA_{905}$ ratio (c) of saline-, erastin- or erastin plus Fer-1-treated 4T1 tumors at 24 h following i.v. injection of 1-NP-FA (56/1.65/20 µM 1-Br-Et/NIR775/IR1048, 200 µL). The corresponding FL and PA images were shown in Fig. 5e&5f. (d) Plot of the PA_{755}/PA_{905} versus the concentration of •OH shows a linear relationship between of the resulting PA_{755}/PA_{905} ratios of 1-NP-FA (56/1.65/20 µM 1-Br-Et/NIR775/IR1048) and •OH concentration from 1-20 µM. (e) Calculated •OH concentration in saline-, erastin- or erastin plus Fer-1-treated 4T1 tumors based on the standard curve in (d). Data are presented as mean ± s.d. (n = 3 independent mice). Statistical differences were analyzed by Student's two-sided t-test. Source data are provided as a Source Data file.

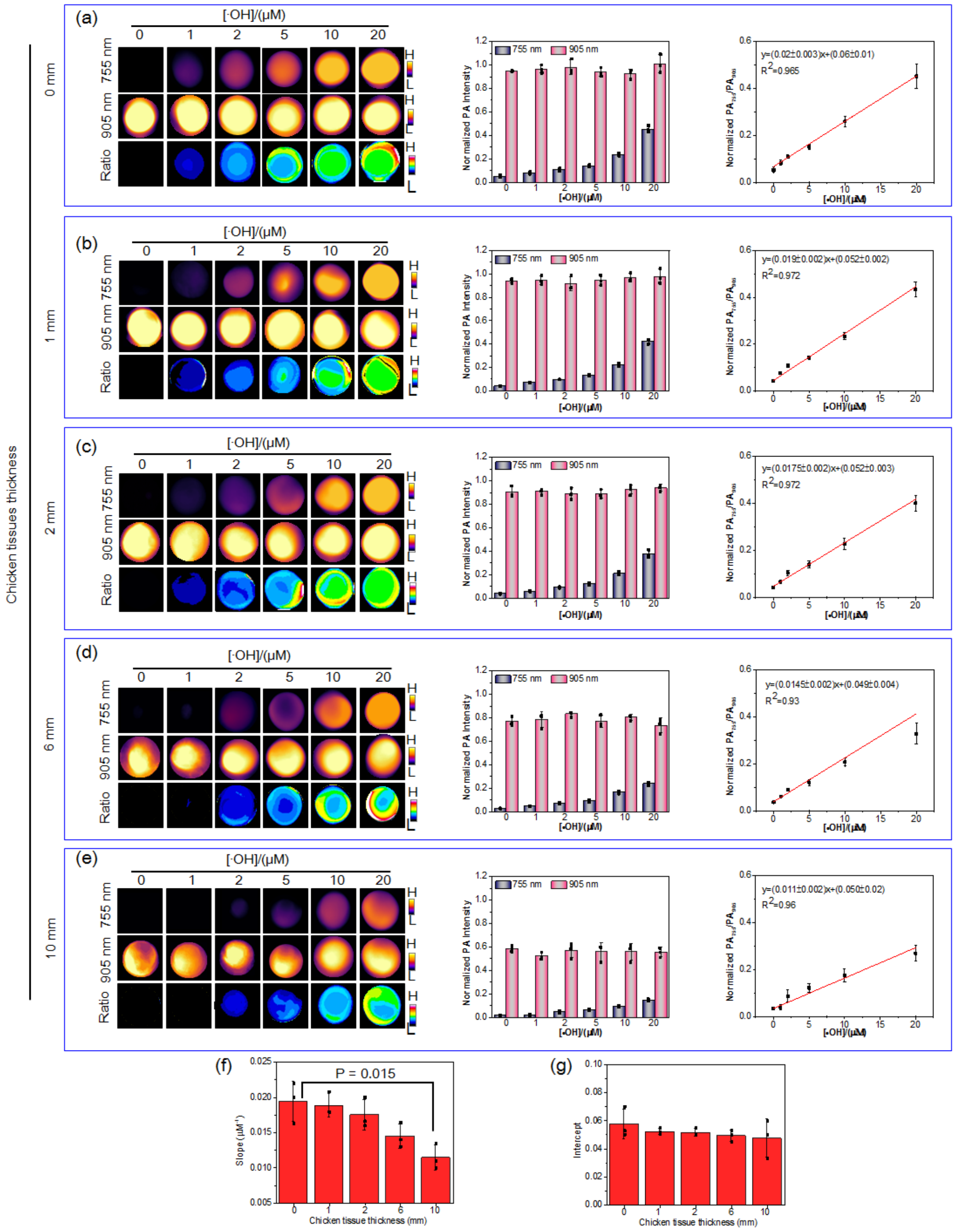

Supplementary Fig. 39 Measurement of •OH in eppendorf tube covered with chicken tissues of different thickness (0, 1, 2, 6 and 10 mm). Photoacoustic (PA) images at 755 and 905 nm, and ratiometric PA_{755}/PA_{905} images (Left), normalized PA intensities at 755 and 905 nm (Middle) and standard curve (Right) of 1-NP-FA (56/1.65/20 μM 1-Br-Et/NIR775/IR1048) upon incubation with indicated concentration of •OH at r.t. for 3 min in tube covered with

chicken tissues of different thickness (0 (a), 1 (b), 2 (c), 6 (d) and 10 mm (e)). Comparison of the slopes (f) and intercepts (g) of standard curves with different thickness of chicken tissues. Data are presented as mean ± s.d. (n = 3 independent samples). Statistical differences were analyzed by Student's two-sided t-test. It was found that the PA intensities at 755 and 905 nm decreased as the thickness of chicken tissue increased from 0 to 10 mm for each incubation solution, presumably caused by the intrinsic scattering and absorption of light by the tissues. By dividing the PA intensity at 755 nm to 905 nm, the PA_{755}/PA_{905} ratios of each solution slightly decreased as the tissue thickness increased, presumably owing to the reduced tissue penetration ability of 755 nm photons compared to that of the 905 nm photons. However, the PA_{755}/PA_{905} ratios all increased linearly versus •OH concentration from 1 to 20 μM under each thickness of chicken tissue. Moreover, though the slopes and intercepts at y-axis of the working curves decreased with the blocking tissue thickness, there were no significant difference when the thickness of blocking tissue was from 1 to 6 mm compared to that of 0 mm; when the blocking tissue thickness was at 10 mm, the slope of the working curve was found to be only 0.011 ± 0.002, significantly lower than that of 0.019 ± 0.003. These results suggest that the working curves could be suitable for the quantification of •OH concentration when the blocking tissue thickness was less than 6 mm, but not accurate for 10 mm thickness of blocking tissues. Source data are provided as a Source Data file.

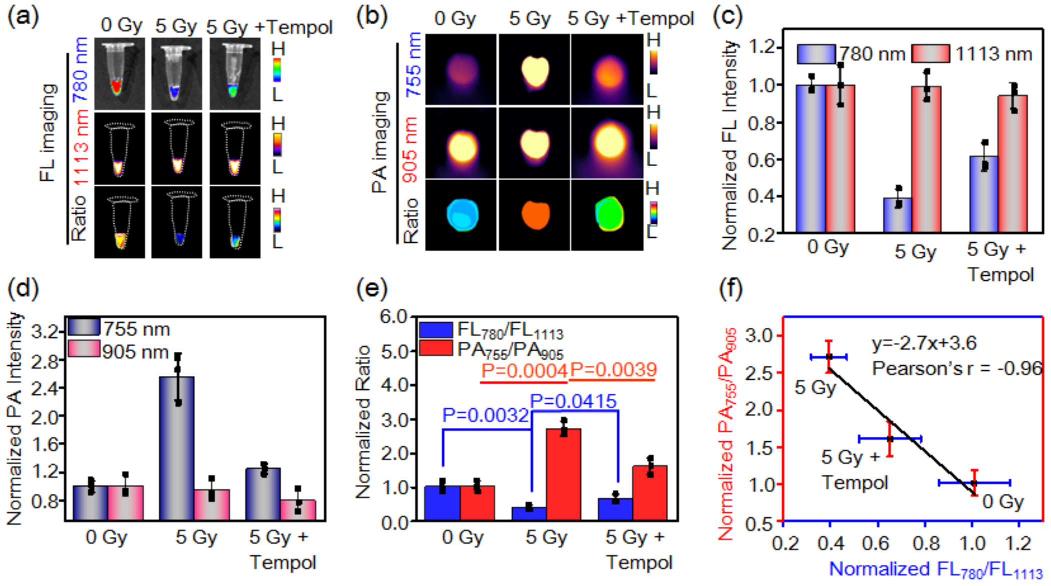

Supplementary Fig. 40 Ratiometric fluorescence/photoacoustic (FL/PA) imaging of •OH generation in 4T1 cells upon radiotherapy (RT) with X-ray. (a) FL and (b) PA images of 1-NP-FA-loading 4T1 cells upon irradiation with X-ray (0 or 5 Gy) or X-ray (5 Gy) plus tempol**.** 4T1 cells were incubated with 1-NP-FA (56/1.65/20 μM 1-Br-Et/NIR775/IR1048) for 2 h, washed, and then irradiated without (0 Gy) or with X-ray (5 Gy, 1.0 Gy/min for 5 min). To scavenge •OH, 1-NP-FA-treated 4T1 cells were incubated with tempol (200 μM) and then irradiated with X-ray (5 Gy). (c) Normalized FL and (d) PA intensities of 4T1 cells following indicated treatment. (e) FL_{780}/FL_{1113} and PA_{755}/PA_{905} ratios of 1-NP-FA-loading cells upon indicated treatment**.** (f) Plot of the PA_{755}/PA_{905} ratios versus the FL_{780}/FL_{1113} ratios shows strong correlation between them (r = -0.96). Data are presented as mean ± s.d. (n = 3 independent cell pellets). Statistical differences were analyzed by Student's two-sided t-test. Source data are provided as a Source Data file.

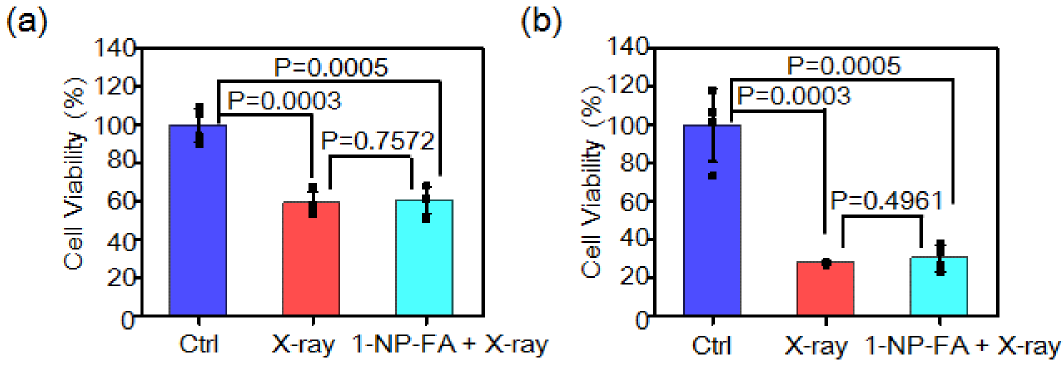

Supplementary Fig. 41 Examination of the radiosensitivity of 4T1 cells using the MTT method. Cell viability of 4T1 cells upon treatment with X-ray (0 or 5 Gy) irradiation in the presence or absence of 1-NP-FA (56/1.65/20 μM 1-Br-Et/NIR775/IR1048, 2 h). The viability was acquired at (a) 48 h and (b) 72 h post X-ray irradiation. Data are presented as mean ± s.d. (n = 4 independent cell experiment). Statistical differences were analyzed by Student's two-sided t-test. Source data are provided as a Source Data file.

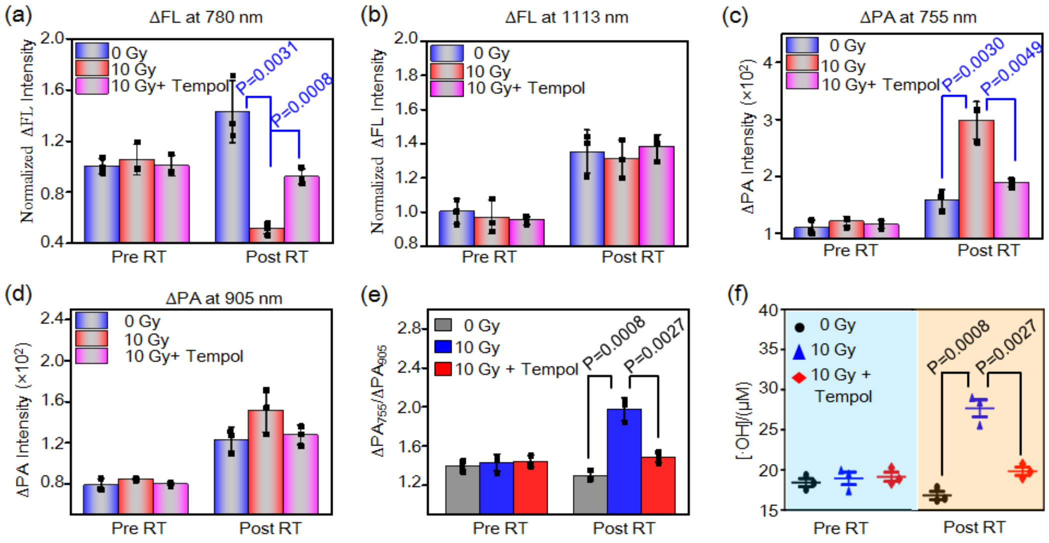

Supplementary Fig. 42 Tumor fluorescence (FL) intensities increment at 780 nm (ΔFL at 780 nm) (a) and 1113 nm (ΔFL at 1113 nm) (b) in mice upon indicated treatment. Tumor photoacoustic (PA) intensities of **1**-NP-FA at 755 nm (ΔPA at 755 nm) (c), 905 nm (ΔPA at 905 nm) (d) and $\Delta PA_{755}/\Delta PA_{905}$ ratios (e) in mice following indicated treatment. (f) Quantification of the •OH concentration of 4T1 tumors following indicated treatment based on plot of the PA_{755}/PA_{905} values versus the concentration of •OH in Supplementary Fig. 38d. 4T1 tumor-bearing mice were i.v. injected with 1-NP-FA (1.68/0.05/0.6 mM 1-Br-Et/NIR775/IR1048, 200 μL). After 12 h, the FL and PA images were both acquired (Pre RT). The tumors were then unirradiated (0 Gy) or irradiated with X-ray (10 Gy, 1.0 Gy/min for 10 min); to inhibit tumor •OH, the mice were i.t. injected with tempol (50 mg/kg), and then irradiated with X-ray (10 Gy). After another 12 h, the FL and PA images were acquired (Post RT). The FL and PA images were shown in Fig. 6b. Data are presented as mean ± s.d. (n = 3 independent mice). Statistical differences were analyzed by Student's two-sided t-test. Source data are provided as a Source Data file.

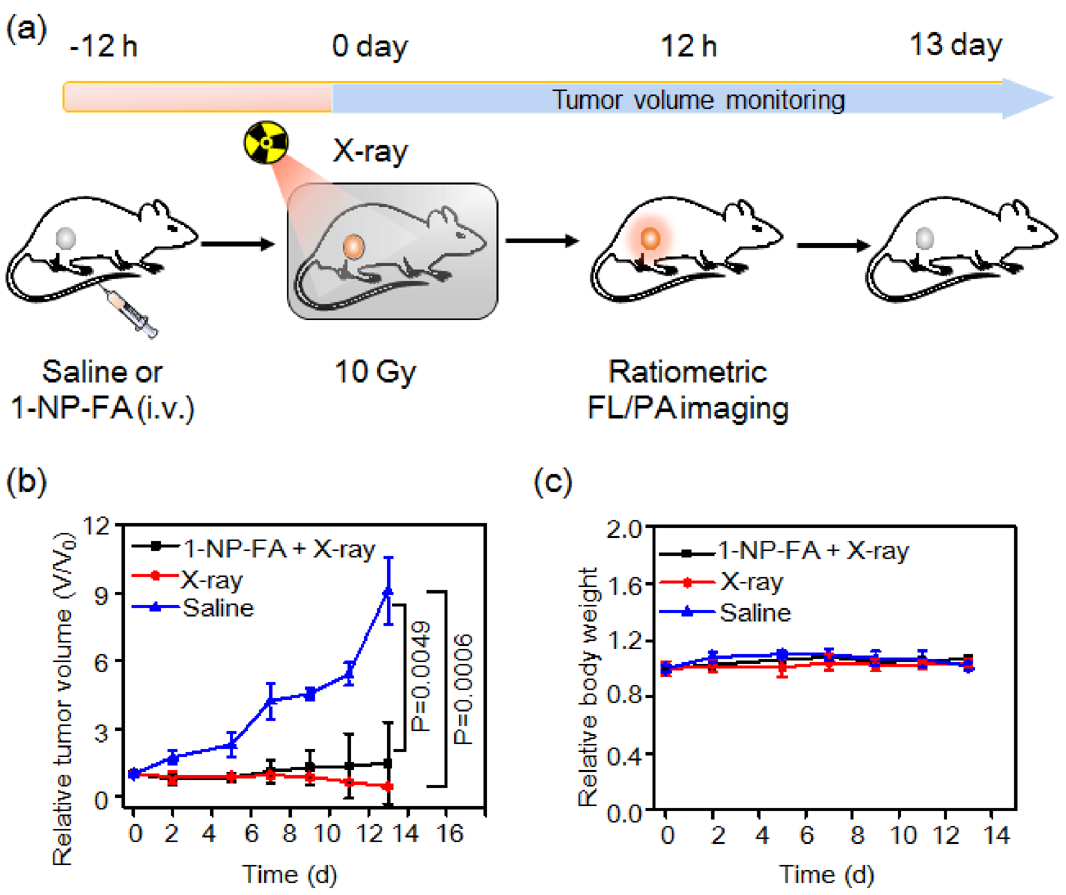

Supplementary Fig. 43 Monitoring of radiotherapy (RT) efficacy against 4T1 tumor in living mice. (a) Cartoon illustrates the general protocol for tumor RT and ratiometric fluorescence/photoacoustic (FL/PA) imaging of tumors with 1-NP-FA. (b) Relative tumor growth curves and (c) relative body weight of mice following indicated treatments. 4T1 tumor-bearing mice were i.v. injected with 1-NP-FA (1.68/0.05/0.6 mM 1-Br-Et/NIR775/IR1048, 200 µL) or saline (1×, pH 7.4, 200 µL). After 12 h, the tumors were then irradiated with X-ray (10 Gy, 1.0 Gy/min for 10 min)**.** The tumors treated with saline only were used as the control (Ctrl). Data are presented as mean ± s.d. (n = 3 independent mice). Statistical differences were analyzed by Student's two-sided t-test. Source data are provided as a Source Data file.

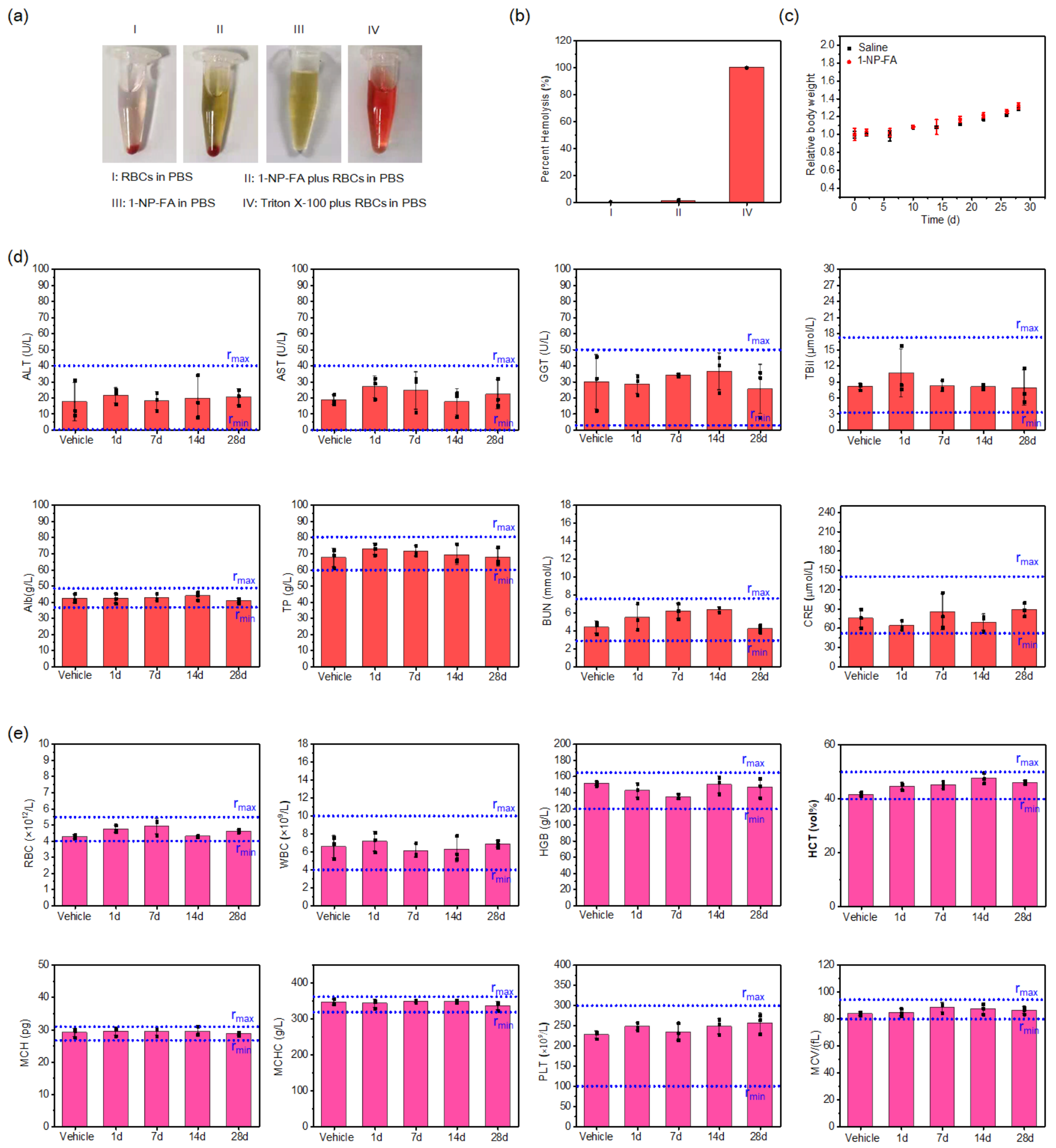

Supplementary Fig. 44 Biosafety assessment of 1-NP-FA in healthy BALB/c mice. (a) Photographs of PBS buffer containing mouse red blood cells (RBCs) (I), RBCs plus 1-NP-FA (II), 1-NP-FA (III), and RBCs plus 0.2% Triton X-100 (IV). (b) Quantification of percent hemolysis (%) in groups I, II and IV in (a). (c) Relative body weights of mice after i.v. injection of 1-NP-FA (1.68/0.05/0.6 mM 1-Br-Et/NIR775/IR1048, 200 μL) or saline. (d) Biochemistry and (e) blood count analysis of mice at 1, 7, 14 and 28 days post i.v. injection of 1-NP-FA (1.68/0.05/0.6 mM 1-Br-Et/NIR775/IR1048, 200 μL). Abbreviations: ALT, alanine aminotransferase; AST, glutamic oxaloacetic transaminase; GGT, γ-glutamyl transpeptidase; TBil, total bilirubin; Alb, albumin; TP, total protein; BUN, blood urea nitrogen; CRE, creatinine;

RBC, red blood cells; WBC, white blood cells; HGB, hemoglobin; HCT, hematocrit; MCH, mean corpuscular hemoglobin; MCHC, mean corpuscular hemoglobin concentration; PLT, platelet count; MCV, mean corpuscular volume Blue short dotted lines indicate the normal reference values ($r_{min} \rightarrow r_{max}$) for BALB/c mice. Data are presented as mean ± s.d. (n = 3 independent mice). Source data are provided as a Source Data file.

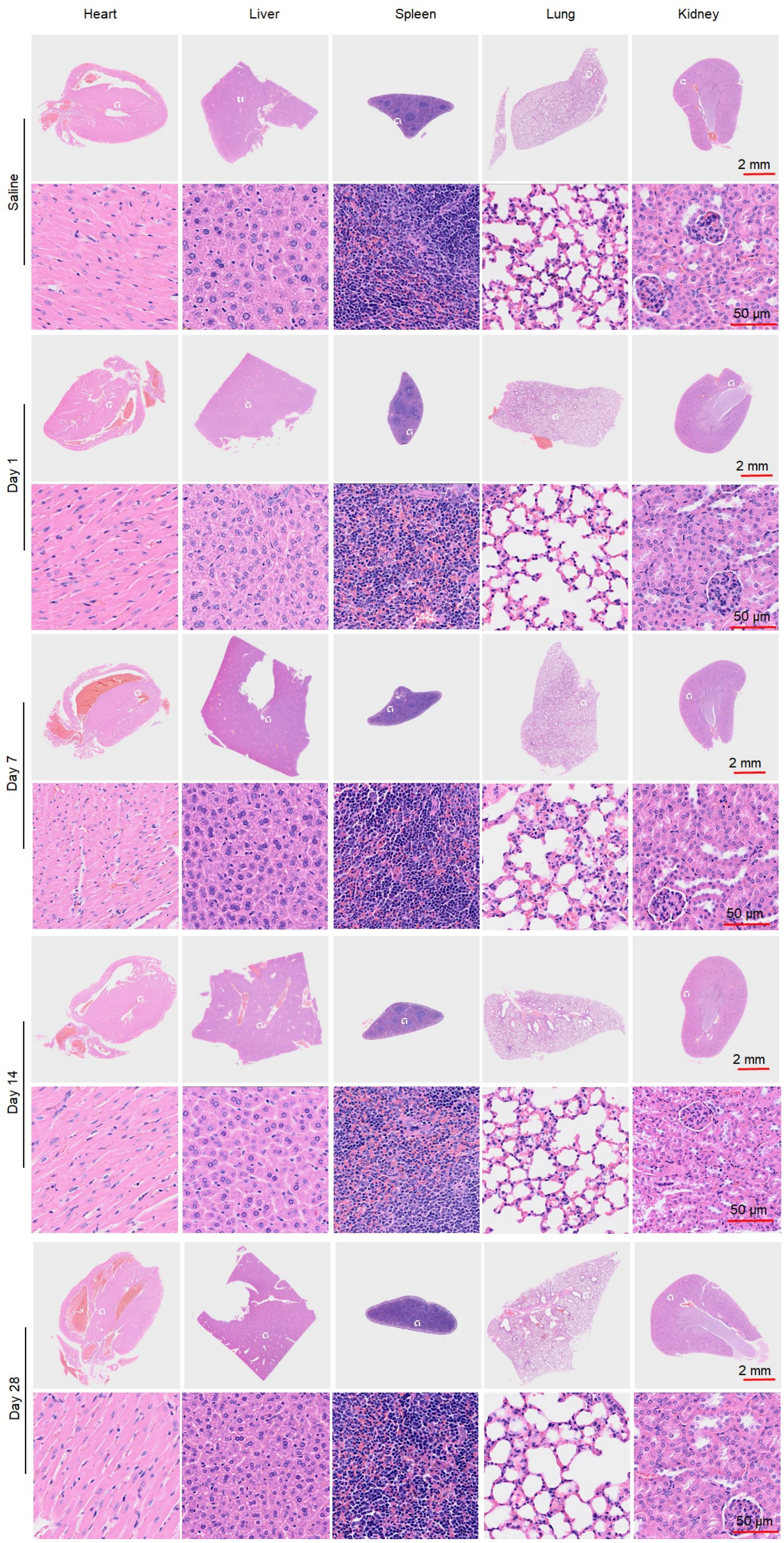
Heart
Liver
Spleen
Lung
Kidney
Saline
Day 1
Day 7
Day 14
Day 28
2 mm
50 μm

Supplementary Fig. 45 Representative hematoxylin-eosin (H&E) staining of dissected main organs from the BALB/c mice. Mice were received i.v. injection of 1-NP-FA (1.68/0.05/0.6 mM 1-Br-Et/NIR775/IR1048, 200 μL), and sacrificed on day 1, 7, 14 and 28. Main organs, including liver, lung, heart, spleen and kidneys were resected, cut into 4-μm slices, and then staining with H&E. The experiments were repeated independently three times with similar results.

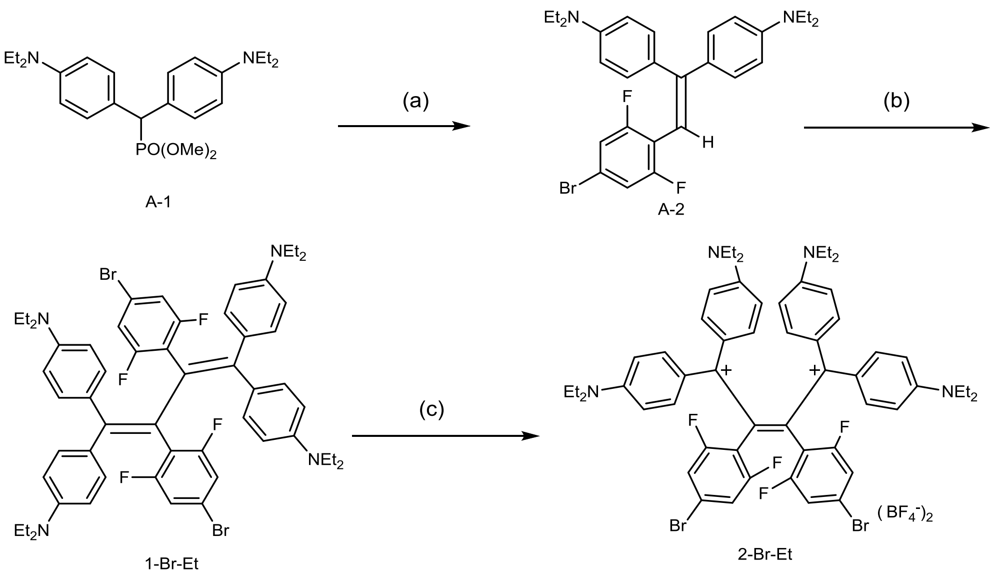

Supplementary Fig. 46 Synthesis of 1-Br-Et. (a) $^{n-}$BuLi, 4-bromo-2,6-difluorobenzaldehyde, 51%; (b) (1) Tris(4-bromophenyl)aminium hexacholoroantimonate, CH_2Cl_2, 0 °C, 1.5 h; (2) Bu_4NF trihydrate, Zn powder, 95%; (c) tris(4-bromophenyl)aminium tetrafluoroborate, CH_2Cl_2, 72%.

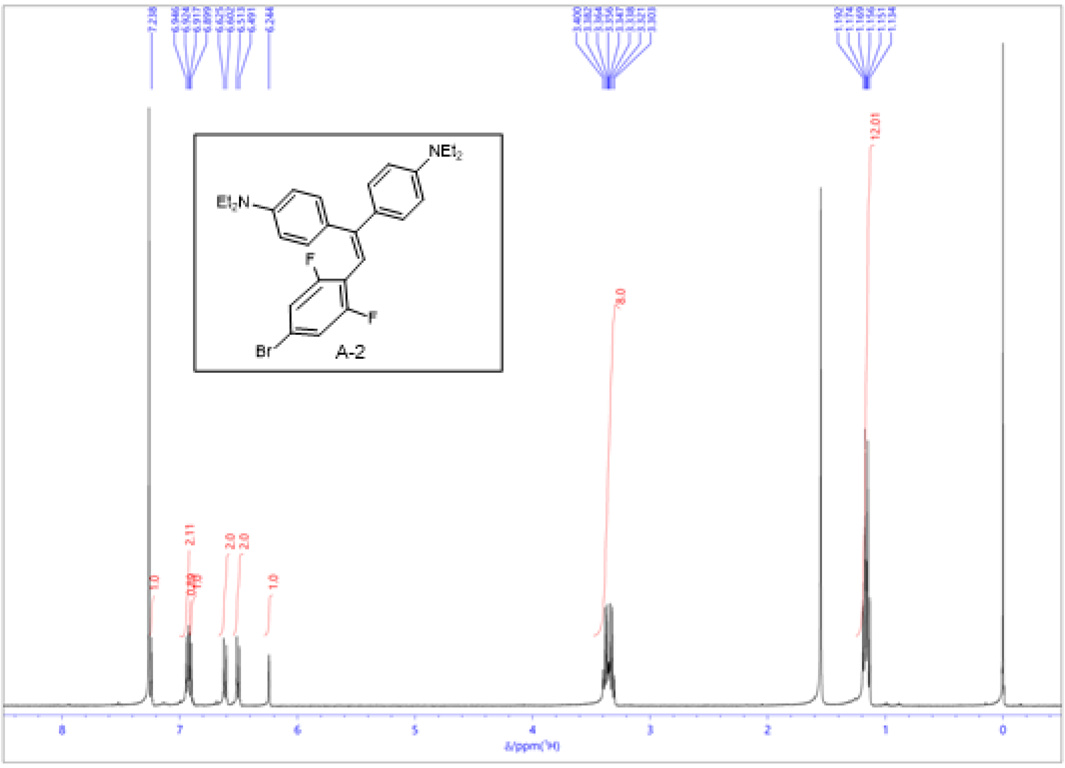

Supplementary Fig. 47 ^{1}H NMR spectra of 1-Br-Et-precursor (A-2) in $CDCl_3$.

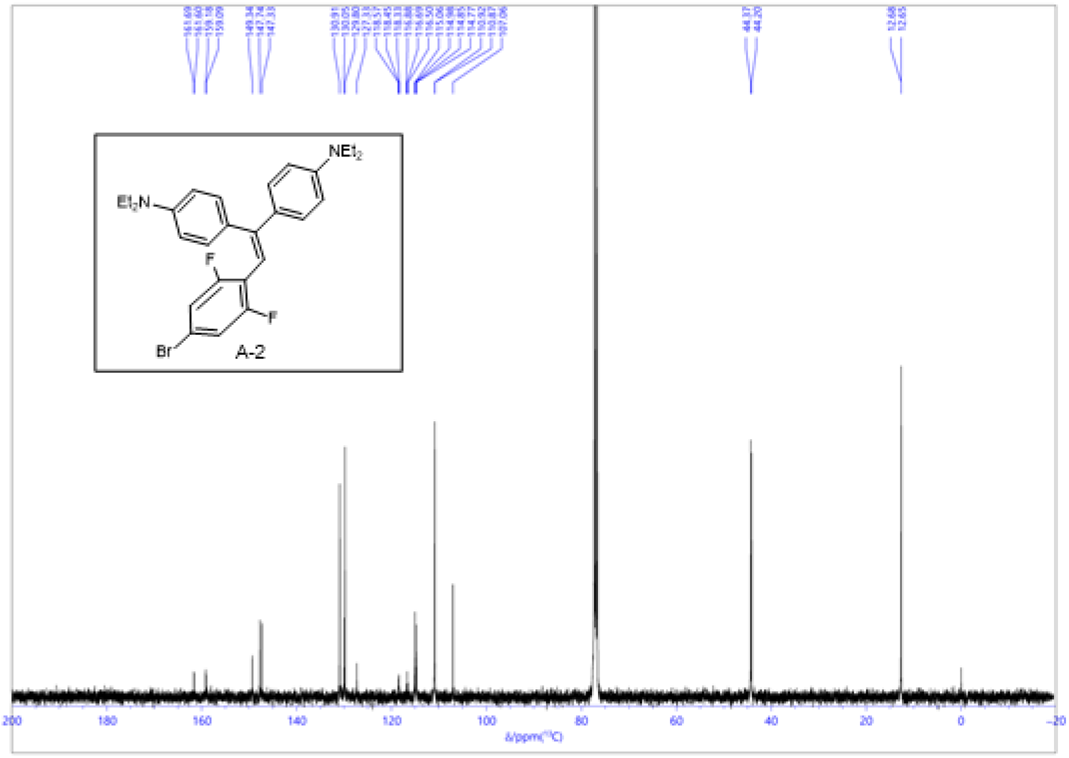

Supplementary Fig. 48 ^{13}C NMR spectra of 1-Br-Et-precursor (A-2) in $CDCl_3$.

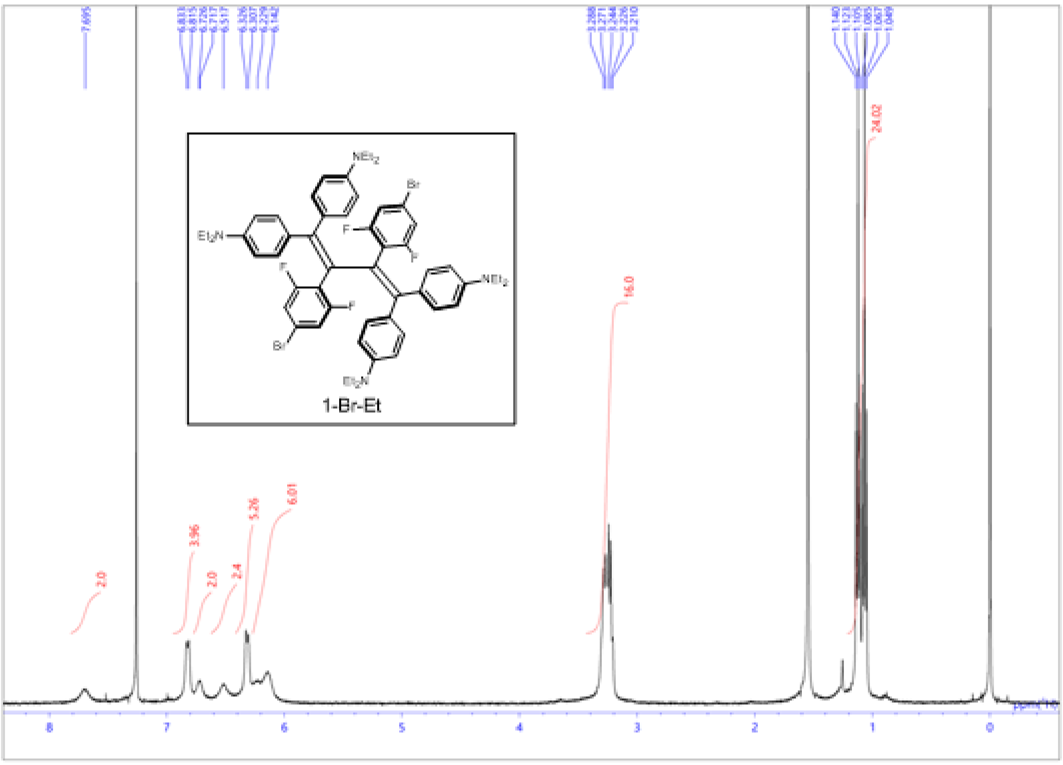

Supplementary Fig. 49 ^{1}H NMR spectra of 1-Br-Et in $CDCl_3$.

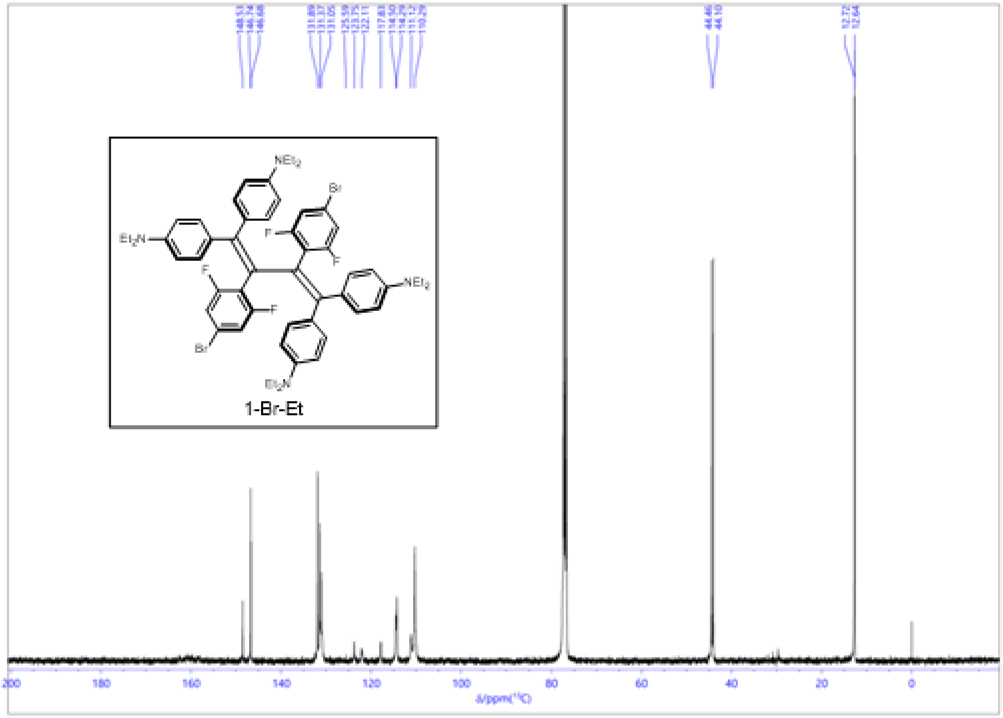

Supplementary Fig. 50 ^{13}C NMR spectra of 1-Br-Et in $CDCl_3$.

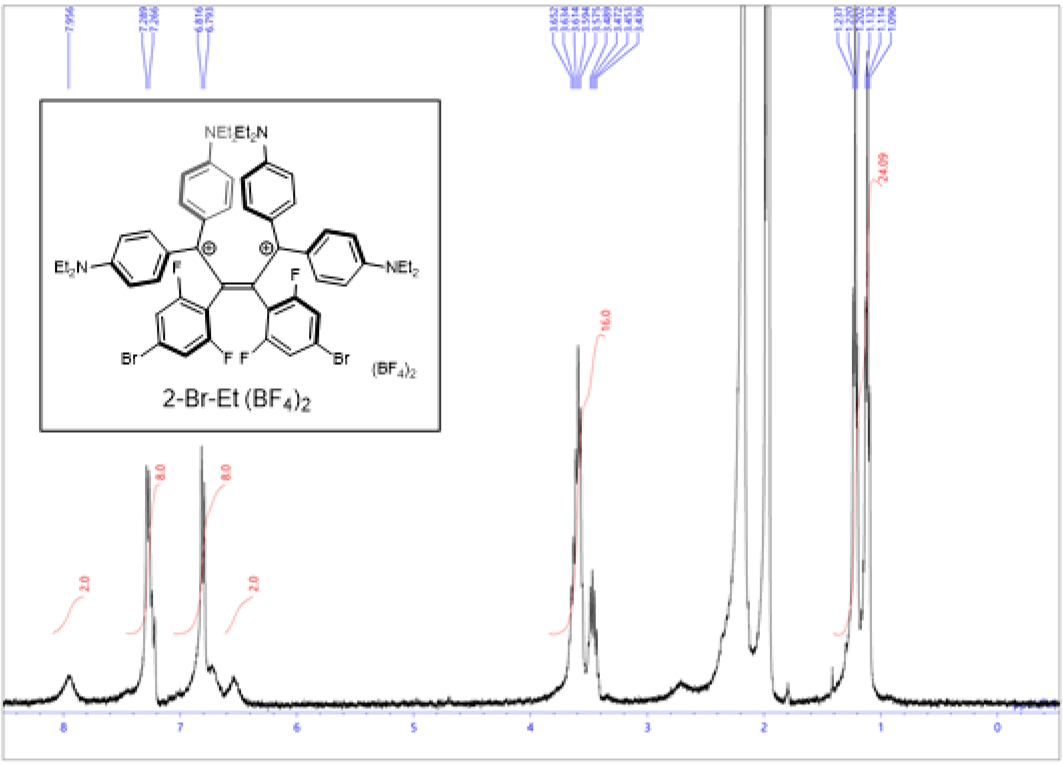

Supplementary Fig. 51 ^{1}H NMR spectra of 2-Br-Et $(BF_4)_2$ in CD_3CN.

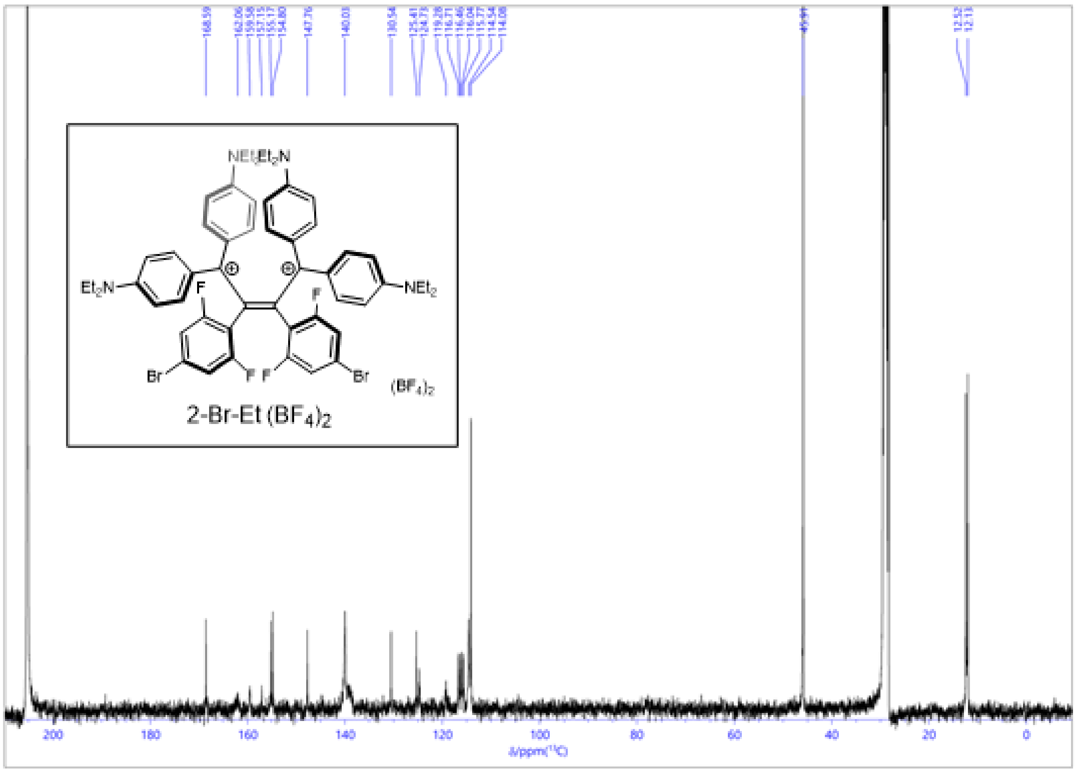

Supplementary Fig. 52 ^{13}C NMR spectra of 2-Br-Et $(BF_4)_2$ in acetone-d_6.

Crystal structures

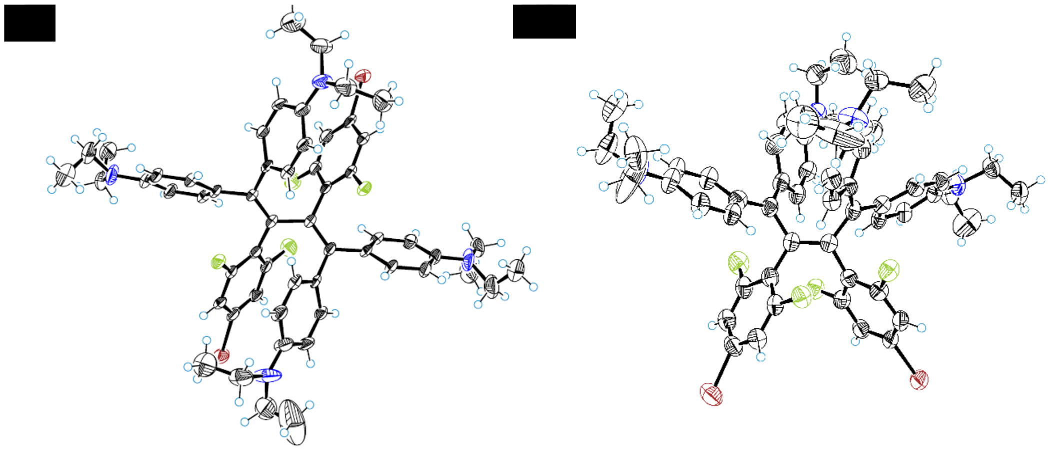

Supplementary Fig. 53 Oak Ridge Thermal Ellipsoid Plot Program (ORTEP) drawings of (a) 1-Br-Et and (b) 2-Br-Et $(BF_4)_2$. The counterions are omitted for clarity. Thermal ellipsoids are shown at the 50% probability level.

3. Supplementary Tables

Supplementary Table 1. The redox potentials of •OH, HClO, H_2O_2, $^1O_2$, $O_2^{\bullet -}$, $ONOO^-$ and HOO^-.

ROS	•OH	HClO	H_2O_2	$^1O_2$	$O_2^{\bullet -}$	$ONOO^-$	HOO^-
Redox potentials (eV)	2.73	1.2	1.76	2.2	0.9	1.2	0.86
References	2, 3	4	5	6	4, 7	8, 9	10

Supplementary Table 2. Summary of the physicochemical properties of NPs.

NPs	Size (nm)[a]	PDI [a]	ξ (mV)[a]	LE (%)[b]	LC (%)[c]
				1-Br-Et/NIR775/IR1048	
1-NP	57.5 ± 3.5	0.229 ± 0.03	-27.8 ± 2.8	100/100/100	5.6/0.14/1.24
1-NP-FA	56.0 ± 2.3	0.252 ± 0.02	-39.9 ± 5.3	100/100/100	5.6/0.14/0.24

[a] Size, polydispersity index (PDI) and Zeta potential (ξ) were determined by 90 Plus/ BI-MAS equipment (Brookhaven, USA). Error bars represent the standard deviation of three separate measurements (n = 3). [b] Loading efficiency (LE). [c] Loading capacity (LC).

Supplementary Table 3. Summary of reported optical imaging probes for •OH detection in biological systems.

Probe [a]	Signal type	Emission [nm]	Turn-on ratio (fold)	LOD [b]	Reaction Kinetics [c]	Biological applications	Refs.
H-V	Turn on	652	450	8.6 nM	30 min (5 equiv. of •OH)	Cells	11
CMS-UCNPs@Azo	Turn on	545	N.D.[d]	0.10 fM	N.D.[d]	Cells; Liver and tumor slices	12
**P2**	Turn on	550	200	0.04 μM	20 min (1 equiv. of •OH)	Cells	13
MD-B	Turn on	500	N.D.[d]	2.4 nM	N.D.[d]	Cells; Brain tissue slices	14
TPA@GQDs	Turn on	430	N.D.[d]	12 nM	N.D.[d]	Cells	15
DNA-Ag NCs	Turn off	550	N.D.[d]	N.D.[d]	N.D.[d]	Cells	16
Nanoprobe	Turn on	520	N.D.[d]	N.D.[d]	15 min	Cells	17
EROH	Turn on	446; 525	N.D.[d]	7 μM	90 min (500 equiv. of •OH)	Cells	18
RThy	Turn on	550	50	8 nM	15 min (20 equiv. of •OH)	Cells; Zebrafish	19
**1**-Red	Turn on	N.D.[d]	125	N.D.[d]	N.D.[d]	Cells	20

Probe **2b**	Turn on	665	N.D.[d]	10 μM	N.D.[d]	Cells	21
Probe **1**	Turn on	650	N.D.[d]	0.05 μM	N.D.[d]	Cells	22
MB-UCNP@PSS	Turn on	650	N.D.[d]	2 nM	N.D.[d]	Liver tissue slices	23
Brite/DEVD@LMWC	Turn on	663	N.D.[d]	N.D.[d]	30 min	Cells; Kidney tissue slices	24
CCy-OH	Turn on	604	60	N.D.[d]	0.5 min (100 equiv. of •OH)	Cells	25
Rhodamine nitroxide probe	Turn on	588	N.D.[d]	N.D.[d]	N.D.[d]	Cells	26
OHP	Turn off	514	N.D.[d]	11 nM	20 min	Cells	27
FHZ	Turn on	486	19	3 nM	1 min (20 equiv. of •OH)	Cells; Zebrafish	28
MPT-Cy2	Turn on	590	N.D.[d]	1.16 μM	N.D.[d]	Cells; Bacteria; Zebrafish	29
HKOH-1	Turn on	520	> 200	390 nM	10 s	Cells	30
mOG-SWUCNPs	Turn on	478	N.D.[d]	1.2 fM	N.D.[d]	Cells; Liver tissue slices	31
**1**	Turn on	500	200	0.1 μM	1 min (10 equiv. of •OH)	Cells	32
Probe **1**	Turn on	653	122	38 nM	30 min (5 equiv. of •OH)	Cells	33
CCA-MCD	Turn on	443	3.01	N.D.[d]	N.D.[d]	Cells	34
Probe **1**	Turn on	550	N.D.[d]	N.D.[d]	N.D.[d]	Cells	35
TEMPO-BDP	Turn on	601	<16	18 pM	5 min (5 equiv. of •OH)	Cells	36
**III**	Turn on	585	N.D.[d]	N.D.[d]	30 min (30equiv. of •OH)	Cells	37
Hydro-1080	Turn on	1044	N.D.[d]	0.5 nM	40 min (0.2 equiv. of •OH)	Cells; Liver injury	38
**1a**	Turn on	794	N.D.[d]	N.D.[d]	N.D.[d]	Cells	39
Hydrocyanine-NC	Turn on	693	N.D.[d]	N.D.[d]	N.D.[d]	Cells	40
GQD-HydroIR783	Ratiometric	I_{800}/I_{520}	N.D.[d]	N.D.[d]	N.D.[d]	Cells; Mice (peritoneal cavity)	41
rUCNP	Ratiometric	I_{800}/I_{540}	N.D.[d]	2 nM	N.D.[d]	Liver inflammation	42
SWIR	Ratiometric	I_{654}/I_{1550}	35.12	4.20 μM	20 min	Arthritis of mice	43
Probe **4**	Ratiometric	I_{492}/I_{652}	826	30.8 nM	70 min (1 equiv. of •OH)	Cells	44
**1**	Ratiometric	F_{418}/F_{552}	53.2	0.2 μM	2 min (50 equiv. of •OH)	Cells	45

CCA@TPP@CDs	Ratiometric	$I_{420-500}/I_{530-610}$	N.D.[d]	70 nM	30 min (60 mg mL^{-1},100 μM •OH)	Cells	46
Nanohybrid probe	Ratiometric	I_{455}/I_{620}	2.3	1.65 μM	N.D.[d]	Cells	47
Eu/DPA-TA	Ratiometric	F_{445}/F_{615}	133	0.5 μM	10 min	Serum	48
RCA-AgNCs	Ratiometric	$I_{500-560}/I_{610-700}$	10	58 nM	N.D.[d]	Cells	49
Rho-Bob	Ratiometric	I_{590}/I_{630}	N.D.[d]	0.68 μM	N.D.[d]	Cells; Zebrafishes	50
CONER	Ratiometric	I_{450}/I_{528}	7	0.73 μM	N.D.[d]	Cells;	51
nanoGUMBOS	Ratiometric	I_{662}/I_{589}	<4	769 nM	N.D.[d]	Cells	52
AuNC@HPF	Ratiometric	I_{515}/I_{637}	N.D.[d]	0.68 μM	15 min	Cells	53
dLys-AgNCs	Ratiometric	F_{450}/F_{640}	N.D.[d]	0.2 μM	10 min	Cells	54
Prrobe **1**	Ratiometric	I_{651}/I_{495}	211	N.D.[d]	120 min (25 equiv. of •OH)	Cells	55
Si QDs-Ce6	Ratiometric	I_{660}/I_{490}	3	0.97 μM	N.D.[d]	Cells	56
**1**-NP	Ratiometric FL&PA	FL_{780}/FL_{1113} PA_{755}/PA_{905}	1026; 22	3.69 nM 0.24 μM	3 min (3.5 equiv. •OH); $k_2 = 748.8\ M^{-1}\ s^{-1}$	Mouse tumor •OH	This work

[a] The name of each probe is the one shown in each ref. [b] LOD: limitation of detection. [c] Second-order reaction rate. If there is no value reported, we just list the time to achieve the maximum activation. [d] N.D.: Not Determined.

Supplementary Table 4. Summary of ratiometric fluorescent/photoacoustic (FL/PA) imaging probes.

Probes	Signals	Probe	Wavelength	Analyte	In vivo Application	refs
HS-CyBz	Ratiometric FL Ratiometric PA	Small molecule probe	FL_{630}/FL_{805} PA_{825}/PA_{775}	Hydrogen sulfide	Detection of H_2S in liver	57
AS-Cy-NO_2	Ratiometric FL Ratiometric PA	Small molecule probe	FL_{773}/FL_{733} PA_{730}/PA_{670}	Nitroreductase	Valuation of hypoxia in tumor	58
SPNP	Ratiometric FL Ratiometric PA	Nanoprobe	FL_{740}/FL_{820} PA_{700}/PA_{760}	Granzyme B	Imaging of immune activation	59
ATP-pH	Ratiometric FL Ratiometric PA	Small molecule probe	FL_{760}/FL_{690} PA_{740}/PA_{680}	ATP/pH	Imaging tumor	60
C-HAS-BPOx-IR825	Ratiometric FL Ratiometric PA	Nanoprobe	FL_{710}/FL_{640} PA_{680}/PA_{825}	pH	Imaging tumor	61
**1**-NP-FA	Ratiometric FL Ratiometric PA	Nanoprobe	FL_{780}/FL_{1113} PA_{755}/PA_{905}	•OH	Detection of •OH in tumor to predict tumor radiotherapy efficacy	Our work

4. Supplementary Datasets

Supplementary Dataset 1: Summary of the coordinates and energies of stationary points regarding the DFT calculations. See independent Excel file (Supplementary Dataset 1) for descriptions.

References:

1. Ishigaki, Y. et al. Hexaarylbutadiene: A versatile scaffold with tunable redox properties towards organic near-infrared electrochromic material. *Chem. Asian J.* **15**, 1147 (2020).

2. Wang, H. et al. Hydroxyl radicals and reactive chlorine species generation via E^+-ozonation process and their contribution for concentrated leachate disposal. *Chem. Eng. J.* **360**,721-727 (2019).

3. Pignatello, J. J., Oliveros, E. & MacKay, A. Advanced oxidation processes for organic contaminant destruction based on the Fenton reaction and related chemistry. *Crit. Rev. Environ. Sci. Technol.* **36**, 1-84 (2006).

4. Oushiki, D. et al. Development and application of a near-infrared fluorescence probe for oxidative stress based on differential reactivity of linked cyanine dyes. *J. Am. Chem. Soc.*, **132**, 2795-2801 (2010).

5. Sitta, E., Gómez-MarínA, A. M., Aldaz, A., Feliu, A. M. Electrocatalysis of H_2O_2 reduction/oxidation at model platinum surfaces. *Electrochem. commun.* **33**, 39-42 (2013).

6. Yi, Q. et al Singlet oxygen triggered by superoxide radicals in a molybdenum cocatalytic Fenton reaction with enhanced REDOX activity in the environment. *Environ. Sci. Technol.*, **53**, 9725-9733 (2019).

7. Winterbourn, C. C. Biological chemistry of superoxide radicals. ChemTexts, 6, 7 (2020).

8. Koppenol, W. H., Moreno, J. J., Pryor, W. A., Ischiropoulos, H. and Beckman,J. S. Peroxynitrite, a cloaked oxidant formed by nitric oxide and superoxide. *Chem. Res. Toxicol.* **5**, 834-842 (1992).

9. Radi, R. Peroxynitrite, a stealthy biological oxidant. *J. Biol. Chem.* **288**, 26464-26472 (2013).

10. Zhang, J. PEM Fuel Cell Electrocatalysts and Catalyst Layers Springer, London, UK 89-134 (2008).

11. Li, H. *et al.* Ferroptosis accompanied by •OH generation and cytoplasmic viscosity increase revealed via dual-functional fluorescence probe. *J. Am. Chem. Soc.* **141**, 18301-18307 (2019).

12. Song, X. *et al.* Dual-activator codoped upconversion nanoprobe with core–multishell structure for in vitro and in vivo detection of hydroxyl radical. *Anal. Chem.* **89**, 11021-11026 (2017).

13. Zeng, L. *et al.* Visualizing the regulation of hydroxyl radical level by superoxide dismutase via a specific molecular probe. *Anal. Chem.* **90**, 1317-1324 (2018).

14. Wang, X. *et al.* Illuminating the function of the hydroxyl radical in the brains of mice with depression phenotypes by two - photon fluorescence imaging. *Angew. Chem. Int. Ed.* **58**, 4674-4678 (2019).

15. Hai, X., Guo, Z., Lin, X., Chen, X. & Wang, J. Fluorescent TPA@ GQDs probe for sensitive assay and quantitative imaging of hydroxyl radicals in living cells. *ACS Appl. Mater. Inter.* **10**, 5853-5861 (2018).

16. Zhang, L. *et al.* DNA-templated Ag nanoclusters as fluorescent probes for sensing and intracellular imaging of hydroxyl radicals. *Talanta* **118**, 339-347 (2014).

17. Ma, B., Lu, M., Yu, B.-Y. & Tian, J. A galactose-mediated targeting nanoprobe for intracellular hydroxyl radical imaging to predict drug-induced liver injury. *RSC adv.* **8**, 22062-22068 (2018).

18. Zhao, Y. *et al.* An endoplasmic reticulum-targeting fluorescent probe for imaging •OH in living cells. *Chem. Commun.* **56**, 6344-6347 (2020).

19. Yuan, G., Ding, H., Sun, H., Zhou, L. & Lin, Q. A mitochondrion-targeting turn-on fluorescent probe detection of endogenous hydroxyl radicals in living cells and zebrafish. *Sensor. Actuator., B* **296**, 126706 (2019).

20. Benitez-Martin, C. *et al.* A turn-on two-photon fluorescent probe for detecting lysosomal hydroxyl radicals in living cells. *Sensor. Actuator., B* **284**, 744-750 (2019).

21. Qu, X. & Shen, Z. A highly selective NIR fluorescent turn-on probe for hydroxyl radical and its application in living cell images. *Front. Chem.* **7**, 598 (2019).

22. Qu, X., Bian, Y., Chen, Y. & Wei, X. A sensitive BODIPY-based fluorescent probe for detecting endogenous hydroxyl radicals in living cells. *RSC adv.* **10**, 28705-28710 (2020).

23. Yu, G. *et al.* A highly selective and sensitive upconversion nanoprobe for monitoring hydroxyl radicals in living cells and the liver. *Sci. China Life Sci.* (2020).

24. Tong, Y., Huang, X., Lu, M., Yu, B.-Y. & Tian, J. Prediction of drug-induced nephrotoxicity with a

hydroxyl radical and caspase light-up dual-signal nanoprobe. *Anal. Chem.* **90**, 3556-3562 (2018).

25. Wang, J.-Y. *et al.* A fast-responsive turn on fluorescent probe for detecting endogenous hydroxyl radicals based on a hybrid carbazole-cyanine platform. *Sensor. Actuator., B* **236**, 60-66 (2016).

26. Cao, L., Wu, Q., Li, Q., Shao, S. & Guo, Y. Fluorescence and HPLC detection of hydroxyl radical by a rhodamine-nitroxide probe and its application in cell imaging. *J. Fluoresc.* **24**, 313-318 (2014).

27. Lei, K. *et al.* Sensitive determination of endogenous hydroxyl radical in live cell by a BODIPY based fluorescent probe. *Talanta* **170**, 314-321 (2017).

28. Zhang, R. *et al.* Real-time discrimination and versatile profiling of spontaneous reactive oxygen species in living organisms with a single fluorescent probe. *J. Am. Chem. Soc.* **138**, 3769-3778 (2016).

29. Liu, F., Du, J., Song, D., Xu, M. & Sun, G. A sensitive fluorescent sensor for the detection of endogenous hydroxyl radicals in living cells and bacteria and direct imaging with respect to its ecotoxicity in living zebra fish. *Chem. Commun.* **52**, 4636-4639 (2016).

30. Bai, X., Huang, Y., Lu, M. & Yang, D. HKOH-1: A highly sensitive and selective fluorescent probe for detecting endogenous hydroxyl radicals in living cells. *Angew. Chem. Int. Ed.* **56**, 12873-12877 (2017).

31. Li, Z., Liang, T., Lv, S., Zhuang, Q. & Liu, Z. A rationally designed upconversion nanoprobe for in vivo detection of hydroxyl radical. *J. Am. Chem. Soc.* **137**, 11179-11185 (2015).

32. Lim, N. Y. *et al.* Novel cyanostilbene-based cluorescent chemoprobe for hydroxyl radicals and its two-photon bioimaging in living cells. *ACS Appl. Bio. Mater.* **2**, 936-942 (2019).

33. Li, H., Li, X., Shi, W., Xu, Y. & Ma, H. Rationally designed fluorescence •OH probe with high sensitivity and selectivity for monitoring the generation of •OH in iron autoxidation without addition of H_2O_2. *Angew. Chem. Int. Ed.* **57**, 12830-12834 (2018).

34. Yu, M., Zhang, G., Wang, W., Niu, J. & Zhang, N. Fluorescence sensing and intracellular imaging for hydroxyl radical using coumarin-modified cyclodextrin derivatives. *Supramol. Chem.* **24**, 799-802 (2012).

35. Kim, M., Ko, S.-K., Kim, H., Shin, I. & Tae, J. Rhodamine cyclic hydrazide as a fluorescent probe for the detection of hydroxyl radicals. *Chem. Commun.* **49**, 7959-7961 (2013).

36. Li, P. *et al.* A new highly selective and sensitive assay for fluorescence imaging of •OH in living cells: effectively avoiding the interference of peroxynitrite. *Chem. Eur. J* **16**, 1834-1840 (2010).

37. Yapici, N. B. *et al.* New rhodamine nitroxide based fluorescent probes for intracellular hydroxyl radical identification in living cells. *Org. Lett.* **14**, 50-53 (2012).

38. Feng, W. *et al.* Lighting Up NIR-II Fluorescence in Vivo: An activable probe for noninvasive hydroxyl radical imaging. *Anal. Chem.* **91**, 15757-15762 (2019).

39. Al-Karmi, S. *et al.* Preparation of an ^{18}F-labeled hydrocyanine dye as a multimodal probe for reactive oxygen species. *Chem. Eur. J* **23**, 254-258 (2017).

40. Kim, J.-Y., Choi, W. I., Kim, Y. H. & Tae, G. Highly selective in-vivo imaging of tumor as an inflammation site by ROS detection using hydrocyanine-conjugated, functional nano-carriers. *J. Control. Release* **156**, 398-405 (2011).

41. Liu, R. *et al.* Design of a new near-infrared ratiometric fluorescent nanoprobe for real-time imaging of superoxide anions and hydroxyl radicals in live cells and in situ tracing of the inflammation process in vivo. *Anal. Chem.* **90**, 4452-4460 (2018).

42. Liu, Y., Jia, Q., Guo, Q., Jiang, A. & Zhou, J. In vivo oxidative stress monitoring through intracellular hydroxyl radicals detection by recyclable upconversion nanoprobes. *Anal. Chem.* **89**, 12299-12305 (2017).

43. Jia, Q., Liu, Y., Duan, Y. & Zhou, J. Interference-free detection of hydroxyl radical and arthritis diagnosis by rare earth-based nanoprobe utilizing SWIR emission as reference. *Anal. Chem.* **91**, 11433-11439 (2019).

44. Wu, B. *et al.* A ratiometric fluorescent probe for the detection of endogenous hydroxyl radicals in living cells. *Talanta* **196**, 317-324 (2019).

45. Meng, L., Wu, Y. & Yi, T. A ratiometric fluorescent probe for the detection of hydroxyl radicals in living cells. *Chem. Commun.* **50**, 4843-4845 (2014).

46. Zhou, D. *et al.* A yellow-emissive carbon nanodot-based ratiometric fluorescent nanosensor for visualization of exogenous and endogenous hydroxyl radicals in the mitochondria of live cells. *J. Mater. Chem., B* **7**, 3737-3744 (2019).

47. Liu, S. *et al.* Dual-emissive fluorescence measurements of hydroxyl radicals using a coumarin-activated silica nanohybrid probe. *Analyst* **141**, 2296-2302 (2016).

48. Gao, J., Li, Q., Wang, C. & Tan, H. Ratiometric detection of hydroxy radicals based on functionalized europium(III) coordination polymers. *Microchimica Acta* **185**, 9 (2017).

49. Li, J., Yu, J., Huang, Y., Zhao, H. & Tian, L. Highly stable and multiemissive silver nanoclusters synthesized in situ in a DNA hydrogel and their application for hydroxyl radical sensing. *ACS Appl. Mater. Interfaces.* **10**, 26075-26083 (2018).

50. Deng, T. *et al.* A new FRET probe for ratiometric fluorescence detecting mitochondria-localized drug activation and imaging endogenous hydroxyl radicals in zebrafish. *Chem. Commun.* **56**, 4432-4435 (2020).

51. Ganea, G. M., Kolic, P. E., El-Zahab, B. & Warner, I. M. Ratiometric coumarin-neutral red (CONER) nanoprobe for detection of hydroxyl radicals. *Anal. Chem.* **83**, 2576-2581 (2011).

52. Cong, M. *et al.* Ratiometric fluorescence detection of hydroxyl radical using cyanine-based binary nanoGUMBOS. *Sensor. Actuator., B* **257**, 993-1000 (2018).

53. Zhuang, M., Ding, C., Zhu, A. & Tian, Y. Ratiometric fluorescence probe for monitoring hydroxyl radical in live cells based on gold nanoclusters. *Anal. Chem.* **86**, 1829-1836 (2014).

54. Liu, F., Bing, T., Shangguan, D., Zhao, M. & Shao, N. Ratiometric fluorescent biosensing of

hydrogen peroxide and hydroxyl radical in living cells with lysozyme-silver nanoclusters: lysozyme as stabilizing ligand and fluorescence signal unit. *Anal. Chem.* **88**, 10631-10638 (2016).

55. Yuan, L., Lin, W. & Song, J. Ratiometric fluorescent detection of intracellular hydroxyl radicals based on a hybrid coumarin-cyanine platform. *Chem. Commun.* **46**, 7930-7932 (2010).

56. Zhao, Q. *et al.* Ratiometric fluorescent silicon quantum dots–Ce6 complex probe for the live cell imaging of highly reactive oxygen species. *ACS Appl. Mater. Interfaces.* **9**, 2052-2058 (2017).

57. Chen, Z. et al. An optical/photoacoustic dual-modality probe: ratiometric in/ex vivo imaging for stimulated H_2S upregulation in mice. *J. Am. Chem. Soc.* **141**, 17973-17977 (2019).

58. Zhang, S. et al A general approach to design dual ratiometric fluorescent and photoacoustic probe for quantitatively visualizing tumor hypoxia levels in vivo. *Angew. Chem. Int. Ed.* (2021). doi: 10.1002/anie.202107076.

59. Zhang, Y. et al. Activatable polymeric nanoprobe for near-infrared fluorescence and photoacoustic imaging of T lymphocytes. *Angew. Chem. Int. Ed.* **60**, 5921-5927 (2021).

60. Liu, X. et al. Dual-stimulus responsive near-infrared reversible ratiometric fluorescent and photoacoustic probe for in vivo tumor imaging. *Anal. Chem.* **93**, 5420-5429 (2021).

61. Chen, Q. et al. A self-assembled albumin-based nanoprobe for in vivo ratiometric photoacoustic pH imaging. *Adv. Mater.* **27**, 6820-6827 (2015).
