# Supplementary material for: Generation of hydroxyl radical-activatable ratiometric near-infrared bimodal probes for early monitoring of tumor response to therapy
Source: Nat Commun. 2021 Oct 22;12:6145. doi: 10.1038/s41467-021-26380-y (PMC8536768; doi:10.1038/s41467-021-26380-y)
Supplement: Supplementary file 2 — Description of Additional Supplementary Files [file 41467_2021_26380_MOESM2_ESM.pdf]

Description of Additional Supplementary Files

File Name: Supplementary Data 1
Description: Summary of the coordinates and energies of stationary points regarding the DFT calculations.
